# Supplementary material for: Evaluation of different infant vaccination schedules incorporating pneumococcal vaccination (The Vietnam Pneumococcal Project): protocol of a randomised controlled trial
Source: BMJ Open. 2018 Jun 8;8(6):e019795. doi: 10.1136/bmjopen-2017-019795 (PMC6009474; doi:10.1136/bmjopen-2017-019795)
Supplement: Supplementary file 1 [file bmjopen-2017-019795supp001.pdf]

APPENDIX 1

Biological Specimens

Specimens include NP swabs, bacterial isolates cultured from NP swabs, serum from whole blood, plasma from whole blood and peripheral blood mononuclear cells (PBMCs). Long-term storage of specimens is at the Pneumococcal Laboratory at MCRI or at the Pasteur Institute of Ho Chi Minh City at -80°C. No genetic or HIV testing will be performed on stored samples and they will not be used to establish a tissue bank. Consent for the long-term storage of samples and their use in potential future studies is recorded on the ICF.

Sample Collection

Blood samples are collected using a butterfly needle into gel vacutainer tubes or sodium heparin vacutainer tubes. The volume of blood collected at different ages is as follows: 2.0ml at 2 months of age; 3.5ml from 3-10 months and 19 months of age; and 3.5ml or 7.5ml at 18 months and 24 months of age, depending on the assays to be conducted. Blood samples collected into gel vacutainer tubes are kept chilled in a cooler box and transported to the Pasteur Institute laboratory the same day. On arrival at the laboratory the samples are centrifuged and the sera divided into up to three aliquots, stored in micro-tubes and frozen at -80°C prior to analysis. For blood samples where plasma cell and memory B cell responses are assessed, samples are collected into sodium heparin vacutainer tubes and transported to the Pasteur Institute laboratory at room temperature the same day. On arrival at the laboratory plasma and PBMCs are separated from each heparinized blood sample by density gradient centrifugation. Plasma are divided into up to four aliquots and stored at -80°C prior to analysis.

NP samples are collected and transported according to standard guidelines.[1] In brief, NP samples are collected using sterile swabs and placed immediately into 1000µL Skim Milk Tryptone Glucose Glycerol (STGG) transport medium. The samples are kept chilled in a cooler box and transported to the Pasteur Institute laboratory the same day. On arrival at the laboratory two aliquots are removed and the aliquots and original sample are frozen at -80°C prior to analysis.

Serotype-specific IgG

Serotype-specific anti-pneumococcal IgG levels to each of the 13 serotypes in 13v-PCV are measured using a modified 3rd generation standardized ELISA at the Pasteur Institute laboratory.[2] Briefly, microtiter wells are coated with 2.5-10 mg/mL pneumococcal polysaccharide, depending on the serotype. This is diluted in phosphate buffered saline by incubating at 22° C overnight. To neutralize unspecified cell wall polysaccharide antibodies, 1/100 diluted serum samples are incubated overnight with 10 mg/mL of cell wall polysaccharide and 30mg/mL of serotype 22F, before further dilutions. A reference serum (89-SF, Food and Drug Administration, Bethesda MD) is used and incubated overnight with 10 mg/mL of cell wall polysaccharide. Horse radish peroxidase conjugated anti-human IgG and the TMB Peroxidase Substrate system is used for detection. Results are expressed as µg/mL of serotype-specific IgG. Three control sera will be used on each plate to assess inter-assay variation.

Opsonophagocytic Assay (OPA)

OPAs are conducted at the Pneumococcal Laboratory at MCRI.[3] Serial dilutions of a heat-inactivated sera, in Hanks balanced salt solution with Mg^{++}, Ca^{++} and gelatine, are made in a 96-well sterile microtitre plate. Frozen stock of pneumococci are thawed, washed and diluted to 5×10^4 CFU/serotype/mL. Standard bacterial dilutions are added to all wells and the plate incubated at RT for 30 min. At 30 min, baby rabbit complement, thawed just prior to use, followed by HL-60 cells (2×10^7 cells/ml) is added to all test wells. A bacterial control (heat inactivated foetal calf serum in place of human sera and no complement) and complement control (no sera) are included on all plates. Plates are placed on a horizontal shaker and incubated for 45 min at 37°C in 5% CO_2. The reaction is stopped at 45 min by placing the plate on ice. A 10µL aliquot of this mixture is then spotted onto Todd-Hewitt broth–yeast extract (0.5%) agar plates. After application of an overlay THYE agar containing selective antibiotic (Optochin, Spectinomycin, Streptomycin or Trimethoprim) and 2,3,5-Triphenyltetrazolium chloride (TTC), the plates are incubated overnight at 37°C in 5% CO_2. After overnight incubation, plates are counted and the results expressed as opsonisation indices (OI) where the OI is defined as the interpolated dilution of serum that kills 50% of bacteria.

Memory B cells

Analysis of the memory B cell response is undertaken at the Pasteur Institute laboratory, by ELISPOT assay.[3] PBMCs are re-suspended in RPMI Foetal Calf Serum (FCS) at a concentration of $2x10^6$ cells/mL and 100µL added to each well of the culture plate containing an antigen cocktail (Staphylococcus aureus Cowan strain – Pansorbin cells (SAC; 1:5000), 2.5µg/mL CpG and 83ng/mL pokeweed mitogen). Plates are incubated at 37^oC with 5% CO_2 and 95% humidity for 5 days. At day 5, cells are harvested and washed and the cell pellet re-suspended in 1mL RPMI-FCS and counted by trypan blue. Cells are then made up to a final concentration of $2x10^6$ cells/mL for seeding onto antigen-coated ELISPOT plates. Multiscreen hydrophobic polyvinyldene difluoride (PVDF) membrane ELISPOT plates coated with anti-IgG (10µg/mL), tetanus toxoid (5µg/mL), diphtheria toxoid (10µg/mL) or pneumococcal polysaccharides conjugated to methylated human serum albumin at concentrations in the range 10-20µg/mL are sealed and incubated overnight at 4^oC. ELISPOT plates are then washed and blocked with RPMI-FCS for 30 minutes at 37^oC with 5% CO2 and 95% humidity. Cultured cells or *ex vivo* PBMCs are washed and seeded at 200 to $2x10^5$ cells/well of the antigen-coated ELISPOT plates in RPMI-FCS and incubated overnight at 37^oC with 5% CO_2 and 95% humidity. Cells are then washed with PBS-T and bound IgG detected with an alkaline phosphatase-conjugated IgG for 4 hours at RT. ELISPOT plates are washed again before addition of an alkaline phosphatase substrate solution (nitroblue tetrazolium plus 5-bromo-4-chloro-3-indoylphosphate in dimethyl formamide). The reaction is stopped with two washes in distilled water. Cells are visualized and counted using an automated ELISPOT reader and software. The total frequency of IgG-secreting antibody-forming cells (AFCs) is used as the positive control and 1,000 IgG AFCs/10^6 cultured PBMCs is the lower cut-off for inclusion in the analysis. Up to $15x10^6$ cells/mL are used for the memory B cell assay at the Pasteur Institute and the remainder of the PBMCs are cryopreserved in liquid nitrogen in aliquots of $8\text{-}10x10^6$ cells/mL for planned T cell assays.

S. pneumoniae identification and serotyping

Identification of *S. pneumoniae* is conducted in line with WHO guidelines.[1] In brief, 50µl swab is plated onto Columbia colistin-nalidixic acid blood agar plates, and identification is primarily based on colonial morphology (flat, with a dimple, 1-3mm in size), α-haemolysis and optochin sensitivity. One colony, plus any additional colonies if morphologically distinct, is sub-cultured onto horse blood agar with an optochin disc. Any colonies that are optochin resistant or intermediately resistant but

otherwise appear to be *S. pneumoniae* are subject to *lytA* PCR,[1] following DNA preparation using the InstaGene matrix (BioRad). All presumptive pneumococci are serotyped, primarily by latex agglutination using reagents produced in-house using antisera from the Statens SerumInstitut, as previously described.[4 5] In summary, pneumococcal culture is made to a 4-5 McFarland density standard and then 10µL of the suspension mixed with 10µL of latex reagent on clear glass slides and rotated for 1 minute. A positive test is indicated by aggregation of latex particles and clearing of the suspension. Isolates that do not react with antisera are subject to *lytA* PCR.

H. influenzae identification

Identification of *H. influenzae* is made from 50µl swab plated onto bacitracin-vancomycin-clindamycin-chocolate-agar. One presumptive *H. influenzae* colony, plus any additional colonies if morphologically distinct, is selected. Colonies are identified as grayish, semi-opaque, smooth, flat or convex, 1-3mm in size. Confirmation is initially demonstrated by X and V growth factor dependence. Capsular and NTHi strains are discriminated using the Phadebact® Haemophilus coagglutination test. All NTHi isolates are tested for beta-lactamase production using nitrocefin.[6] Following identification of presumptive NTHi, DNA is extracted using the InstaGene matrix (BioRad)[7] and tested by *siaT* and *hypD* PCR for discrimination between NTHi and *H. haemolyticus*.[8]

Quantification of *H. influenzae* and pneumococcus

DNA is extracted from 100µl of STGG medium using high-throughput systems (MagNA Pure LC, Roche) using the DNA Isolation Kit II (Bacteria, Fungi) (Roche) incorporating enzymatic digestion. Quantification of *H. influenzae* and pneumococci is then performed using real-time quantitative PCR (qPCR).[9] qPCR targeting the *hpd3* and/or *siaT* gene (*H. influenzae*) or *lytA* gene (pneumococcus) is conducted in 25µl reactions containing 2µl of template DNA on a Stratagene Mx3005 machine using Brilliant III Ultra-Fast qPCR Master Mix (Agilent Technologies) according to the manufacturer's instructions. The density of each bacterial species is assessed in comparison to a set of approximately five reference standards run with each assay to give the density of carriage.

Microarray serotyping

Samples that contain pneumococci are tested by DNA microarray as described previously with minor modifications.[4] Following a culture amplification step (on selective agar such as horse blood agar with 5 µg/ml gentamicin), DNA is extracted

using the Qiacube HT platform (Qiagen). When only a single α-haemolytic colony grows, it is sub-cultured before DNA extraction for microarray. DNA is labelled and then hybridised to the Senti-SP microarray (formally BUGS microarray), scanned on an Agilent scanner, and uploaded to Senti-Net (a cloud based software platform). Serotype-specific density is calculated by multiplying pneumococcal density (measured by *lytA* qPCR) by the relative abundance of each serotype (determined by microarray).

Immunogenicity of *Infanrix-hexa*

The specific IgG to *Haemophilus influenzae* type b (Hib) will be measured by ELISA. High binding ELISA plates are coated with Hib polysaccharide (HBO-HA, the PRP capsular linked to human albumin) antigen and incubated at 37°C for 2 hours and then overnight at 4°C. The plates are washed and blocked with 1% Gelatin in PBS, then loaded with dilutions of standards and patient samples. Following two hours incubation at 37°C, the plates are washed and peroxidase-labelled anti-human IgG is added to each well. Bound specific antibody is detected using the substrate TMB. After the substrate reaction, the intensity of the colour developed is proportional to the amount of IgG-specific antibodies detected in the sample. Results for the samples are determined directly using a standard curve and expressed as µg/mL. Three control sera will be used on each plate to assess inter-assay variation.

The specific IgG to tetanus and diphtheria will be measured using a commercial solid phase ELISA (Genzyme Virotech). The wells are coated with antigen. Specific antibodies of the sample bind to the antigen coated wells and are detected by a secondary enzyme conjugated antibody specific for human IgG. After the substrate reaction, the intensity of the colour developed is proportional to the amount of IgG-specific antibodies detected in the sample. Results for the samples are determined directly using a standard curve and expressed as IU/mL. Two control sera will be used on each plate to assess inter-assay variation.

The Hepatitis B surface antibodies will be measured using AxSym analyzer system. Patient serum is incubated with Micro-particles coated with recombinant HbsAg. Antibody present in the serum binds with antigen on the particles. When this reaction mixture is transferred to the matrix cell, the micro-particles bind irreversibly to the glass fibre matrix. Biotinylated rHBsAg is then added forming an antigen-antibody-antigen complex. Anti-Biotin: Alkaline phosphatase conjugate is dispensed onto the matrix cell and binds with any microparticle-bound antigen-antibody-antigen complex.

The matrix cell is washed to remove any unbound antibody and the substrate 4-Methylumbelliferyl Phosphate is added. The alkaline phosphatase-labelled conjugate catalyses the removal of a phosphate group from the substrate, yielding a fluorescent product, 4-Methylumbelliferone. This fluorescent product is measured and the concentration of anti-HBs in the sample is determined from a calibration curve and will be reported in IU/mL. A positive and negative control will be included in each assay.

The specific IgG to *B. pertussis* (PT) will be measured using a commercial solid phase ELISA (Genzyme Virotech). The wells are coated with antigen. Specific antibodies of the sample bind to the antigen coated wells and are detected by a secondary enzyme conjugated antibody specific for human IgG. After the substrate reaction, the intensity of the colour developed is proportional to the amount of IgG-specific antibodies detected in the sample. Results for the samples are derived using the optical density ratio of the cut-off control and the patient sample and expressed in VE or Virotech Units which have been calibrated with the reference standard IgG anti-Pertussis toxin (Lot 3, 200 U/ml) of the Centre for Biologic Evaluation and Research (CBER), FDA. Three control sera will be used on each plate to assess inter-assay variation.

Appendix 1 - References

1. Satzke C, Turner P, Virolainen-Julkunen A, et al. Standard method for detecting upper respiratory carriage of Streptococcus pneumoniae: updated recommendations from the World Health Organization Pneumococcal Carriage Working Group. *Vaccine* 2013;32:165-79. doi:10.1016/j.vaccine.2013.08.062.
2. Balloch A, Licciardi PV, Leach A, et al. Results from an inter-laboratory comparison of pneumococcal serotype-specific IgG measurement and critical parameters that affect assay performance. *Vaccine* 2010;28:1333-40. doi:10.1016/j.vaccine.2009.11.011.
3. Licciardi PV, Toh ZQ, Clutterbuck EA, et al. No long-term evidence of hyporesponsiveness after use of pneumococcal conjugate vaccine in children previously immunized with pneumococcal polysaccharide vaccine. *The Journal of allergy and clinical immunology* 2016;137:1772-79 e11. doi:10.1016/j.jaci.2015.12.1303.
4. Satzke C, Dunne EM, Porter BD, et al. The PneuCarriage Project: A Multi-Centre Comparative Study to Identify the Best Serotyping Methods for Examining Pneumococcal Carriage in Vaccine Evaluation Studies. *PLoS medicine* 2015;12:e1001903; discussion e03. doi:10.1371/journal.pmed.1001903.
5. Porter BD, Ortika BD, Satzke C. Capsular Serotyping of Streptococcus pneumoniae by latex agglutination. *Journal of visualized experiments : JoVE* 2014:51747. doi:10.3791/51747.
6. Tenover FC, Huang MB, Rasheed JK, et al. Development of PCR assays to detect ampicillin resistance genes in cerebrospinal fluid samples containing Haemophilus influenzae. *J Clin Microbiol* 1994;32:2729-37.
7. Carter J, Hutton S, Sriprakash KS, et al. Culture of the causative organism of donovanosis (Calymmatobacterium granulomatis) in HEp-2 cells. *J Clin Microbiol* 1997;35:2915-7.
8. Price EP, Harris TM, Spargo J, et al. Simultaneous identification of Haemophilus influenzae and Haemophilus haemolyticus using real-time PCR. *Future microbiology* 2017;12:585-93. doi:10.2217/fmb-2016-0215.
9. Smith-Vaughan H, Byun R, Nadkarni M, et al. Measuring nasal bacterial load and its association with otitis media. *BMC Ear Nose Throat Disord* 2006;6:10. doi:10.1186/1472-6815-6-10.
