# Supplementary material for: Evaluation of different infant vaccination schedules incorporating pneumococcal vaccination (The Vietnam Pneumococcal Project): protocol of a randomised controlled trial
Source: BMJ Open. 2018 Jun 8;8(6):e019795. doi: 10.1136/bmjopen-2017-019795 (PMC6009474; doi:10.1136/bmjopen-2017-019795)
Supplement: Supplementary file 2 [file bmjopen-2017-019795supp002.pdf]

APPENDIX 2

Plain Language Statements and Informed Consent Form

These materials were translated into Vietnamese, and back-translated into English, by FHI360. This trial uses two Plain Language Statements, one for participants enrolled at 2 months of age and randomised into Arms A-F, and one for participants enrolled at 18 months of age into Arm G. The same Informed Consent Form is used for participants in all Arms.

menzies
school of health research

PO Box 41096, Casuarina NT 0811, Australia
John Mathews Building (Bldg 58),
Royal Darwin Hospital Campus, Rocklands Dve, Casuarina NT 0810
Ph: 08 8922 8196 | Fax: 08 8927 5187 | Web: www.menzies.edu.au
ABN: 70 413 542 847

discovery for a healthy tomorrow

INFORMATION SHEET: Vietnam Pneumococcal Vaccine Study

This is for you to keep.

Principal Investigators:
Assoc. Prof. Tran Ngoc Huu
Prof. Edward Kim Mulholland

Research Partners:
Pasteur Institute, Ho Chi Minh City
Menzies School of Health Research
Murdoch Childrens Research Institute

Introduction

Health research helps us to understand diseases and find ways to prevent them. Vaccines (like the routine baby injections) are an important way to prevent diseases. Pneumonia is a common problem in Vietnam and throughout the developing world. In the developing world it is the leading cause of death amongst under 5 year olds. A number of germs cause pneumonia but the most common germ is a bacteria called pneumococcus. Pneumococcus can also cause ear infections as well as other, more severe diseases like meningitis (infection around the brain). This germ normally lives in the nose of humans and is spread from person to person by touching or sneezing. There are more than 90 types of this germ but only some types cause serious infections in young children.

Why are we doing the study?

There are vaccines available to protect against infection with pneumococcus. These are called pneumococcal vaccines. Many countries around the world give all their babies a pneumococcal vaccine that protects against 7 types of the pneumococcal disease (7v-PCV). There are two new vaccines which have been developed. Both new vaccines give more protection against pneumococcal disease than the 7v-PCV. Both vaccines have completed all their tests and are licensed and being used by many countries in Europe and the United States. The clinical trials have shown that these vaccines are safe; therefore there is little danger to any child participating in this study. The vaccines are likely to provide some protection from ear infections and pneumonia. Unfortunately the costs of these vaccines are very high, so not all countries in the world can afford them. We are doing this study to find the best ways to protect babies from this germ and also to make it cheaper for countries, like Vietnam, to afford to buy the vaccine.

Benefits of the study

By joining the study your baby can be protected from the commonest pneumococcal germs. Both these vaccines are very expensive and are not presently available to other babies in Vietnam. They have been especially made for use in babies and young children and will protect the babies from the common diseases caused by the pneumococcus. We hope to find a schedule that works and which countries like Vietnam can afford. In addition children will receive 4 doses of *Infanrix-Hexa*: 3 doses during early infancy and a booster dose at either 18 or 19 months of age.

What does the study involve?

The study will include 1400 babies and we will be looking at 7 different vaccine schedules in this study. 1200 babies will be enrolled at 2 months old and will be randomly allocated to 1 of 6 groups. An additional 200 babies will be enrolled at 18 months old to act as controls.

Consent: A study doctor or nurse will discuss the study with each child's parent or legal guardian. They will explain what is involved and ask some questions about the baby's health. If you agree to join the study she will ask you to sign a consent form which says that you agree for your baby to join. If you consent to taking part in the study, she will perform a health check of your baby to make sure your baby is healthy to take part.

Vaccinations & health checks: If you agree to your baby to take part in the study you will need to come to the clinic between 9 and 11 times over a period of 22 months. The study nurse will remind you when you need to come. Like rolling a dice your baby will be allocated to 1 of 6 groups. Your baby will get between one and four doses of one of the two types of Pneumococcal vaccine, either the Prevnar-13 (13v-PCV which covers 13 types of the pneumococcal germ) or the 10v-Synflorix vaccine (which covers 10 types of the pneumococcal germ and may be better at protecting against pneumonia). Depending on which group your baby is randomly placed in will depend on when, how many doses and what type of Pneumococcal vaccine your baby will receive. Your baby will also get an infant vaccine (Infanrix-hexa 6-1) that covers all the diseases (diphtheria, tetanus, pertussis, hepatitis B, polio virus and *Haemophilus influenzae* type B) that are covered by the standard vaccines used in VIetnam. Vaccines will be given by staff from Pasteur Institute Ho Chi Minh City. Your baby will also have regular health checks during the study.

Questionnaire: At the start of the study you will be asked some general questions about your family and your baby's health. These are simply to help us understand how the vaccines work best. The results will be kept confidential (see below).

Blood tests: Up to four blood tests will be taken during the study, by staff from Children's Hospital Number 2. The blood tests are to check the response to the vaccines. If you would prefer, we can put local anesthetic cream on your baby's skin before taking the blood test so that it doesn't hurt as much. The amount of blood taken will vary depending on the age of the child: 2.0mls at 2 months of age; 3.5mls from 3 to 10 and 19 months of age; and 3.5mls or 7.5mls at 18 and 24 months of age.

Nose swabs: Six nose swabs will be taken during the study, at 2, 6, 9, 12, 18 and 24 months of age. The nose swabs are to see if the vaccine will help stop the spread of the pneumococcus from child to child. This will involve putting a cotton wool swab (like a cotton bud) into the baby's nose for a couple of seconds. This may make the baby sneeze and possibly cry briefly – it tickles quite a lot, but doesn't really hurt.

Summary of changes: Additional procedures and vaccines

Groups A-E	*18 months*	*Measles and Rubella given*
	19 months	*Infanrix Hexa given*
	24 months	*Nose swab taken*
Group F	*18 months*	*Infanrix Hexa given*
	19 months	*Measles and Rubella given*<br>*Blood taken*
	24 months	*Nose swab taken*<br>*Blood taken*<br>*Synflorix given*

Hospital record review: If your baby becomes unwell during the study, the staff may need to look at your child's medical records.

Are there any risks?

The vaccines we are using are licensed many countries. As with all vaccines there is likely to be some pain felt, and there is a small risk of soreness and redness where the vaccine was given. Babies in the study will get up to 4 extra injections than they would routinely get. We will check the babies to make sure they don't have any unexpected reactions. We also have a study doctor who will be keeping a record of any serious illnesses that are unlikely to occur during the study.

Confidentiality

All information collected in this study will remain confidential and will be used for research purposes only. All information will be kept secure. Your baby will be given an identification number at the start of the study. Any information collected will use this number and will not include your baby's name. The samples we collect will be sent to overseas laboratories to have further tests. These laboratories will not be given your child's name. We will ask your permission if it is alright for your baby's blood and nose swab samples to be stored indefinitely for other similar tests in the future. This would help us to perform any new pneumococcal test that may be developed in the future. The results of the study will be published in scientific journals and presented at conferences. There will never be details published that would identify your baby.

Voluntary Participation and Withdrawal from the Study

Your baby does not have to take part in the study. Your baby will get the best treatment available and the full attention of the health staff even if they do not participate. You are free to withdraw your baby from the study at any point. This will not affect any of your baby's further health care treatment and there will be no harmful consequences for your baby. If your baby has not had all their pneumococcal vaccines they may not be fully protected against the pneumococcal germs which most commonly affect infants. However, they will still gain some protection from the doses of vaccine received.

Compensation

We will pay 200,000VND towards the transport cost for coming to the clinic for each study visit. If your baby becomes ill or injured as a result of taking part in this clinical study, medical treatment will be provided.

Ethical Approval

This study has been approved by the People's Committee of Ho Chi Minh City. This study has also been approved by the Vietnam Ministry of Health Ethics Committee and the Menzies School of Health Research Ethics Committee, Australia. The ethics committees make sure that the study is being done in the best and safest way. If you have any concerns or complaints regarding the conduct of the research project you are invited to contact:

Vietnam Ministry of Health
Ethics Committee
Phone: 04 62732156

OR

Human Research Ethics Committee of the NT
Department of Health and Menzies School of Health Research
PO Box 41096, Casuarina, NT 0811, Australia
Phone: 61 8 8922 7922
Email: ethics@menzies.edu.au

How is the study funded?

The funding to perform the study is from the National Health and Medical Research Council, Australia and the Bill & Melinda Gates Foundation.

Your Right to Ask Questions

Please feel free to contact us if you have any questions or concerns.

If you have any questions regarding the study activities, please phone:

If you have any questions regarding adverse events, please phone:

Commune Health Centre Number:

menzies
school of health research

PO Box 41096, Casuarina NT 0811, Australia
John Mathews Building (Bldg 58),
Royal Darwin Hospital Campus, Rocklands Dve, Casuarina NT 0810
Ph: 08 8922 8196 | Fax: 08 8927 5187 | Web: www.menzies.edu.au
ABN: 70 413 542 847

discovery for a healthy tomorrow

INFORMATION SHEET: Vietnam Pneumococcal Vaccine Study (Control group)

This is for you to keep.

Principal Investigators:
Assoc. Prof. Tran Ngoc Huu
Prof. Edward Kim Mulholland

Research Partners:
Pasteur Institute, Ho Chi Minh City
Menzies School of Health Research
Murdoch Children's Research Institute

Introduction

Health research helps us to understand diseases and find ways to prevent them. Vaccines (like the routine baby injections) are an important way to prevent diseases. Pneumonia is a common problem in Vietnam and throughout the developing world. In the developing world it is the leading cause of death amongst under 5 year olds. A number of germs cause pneumonia but the most common germ is a bacteria called pneumococcus. Pneumococcus can also cause ear infections as well as other, more severe diseases like meningitis (infection around the brain). This germ normally lives in the nose of humans and is spread from person to person by touching or sneezing. There are more than 90 types of this germ but only some types cause serious infections in young children.

Why are we doing the study?

There are vaccines available to protect against infection with pneumococcus. These are called pneumococcal vaccines. Many countries around the world give all their babies a pneumococcal vaccine that protects against 7 types of the pneumococcal disease (7v-PCV). There are two new vaccines which have been developed. Both new vaccines give more protection against pneumococcal disease than the 7v-PCV.Both vaccines have completed all their tests and are licensed and being used by many countries in Europe and the United States. The clinical trials have shown that these vaccines are safe; therefore there is little danger to any child participating in this study. The vaccines are likely to provide some protection from ear infections and pneumonia. Unfortunately the costs of these vaccines are very high, so not all countries in the world can afford them. We are doing this study to find the best ways to protect babies from this germ and also to make it cheaper for countries, like Vietnam, to afford to buy the vaccine.

Benefits of the study

By joining the study your baby can be protected from the commonest pneumococcal germs. Both these vaccines are very expensive and are not presently available to other babies in Vietnam. They have been especially made for use in babies and young children and will protect the babies from the common diseases caused by the pneumococcus. We hope to find a schedule that works and which countries like Vietnam can afford. In addition your baby will receive a dose of Infanrix-hexa at 18 months of age.

What does the study involve?

The study will include 200 babies to act as comparisons to participants in an existing study of six different vaccine schedules.

Consent: A study doctor or nurse will discuss the study with each child's parent or legal guardian. They will explain what is involved and ask some questions about the baby's health. If you agree to join the study she will ask you to sign a consent form which says that you agree for your baby to join. If you consent to taking part in the study, she will perform a health check of your baby to make sure your baby is healthy to take part.

Vaccinations & health checks: If you agree to your baby to take part in the study you will need to come to the clinic 3 times over a period of 6 months. The study nurse will remind you when you need to come. Your baby will get a single dose of (Infanrix-hexa 6-1) that covers six diseases (diphtheria, tetanus, pertussis, hepatitis B, polio virus and *Haemophilus influenzae* type B) at 18 months of age, a single dose of Measles and Rubella (MR) at 19 months of age and a single dose of Pneumococcal vaccine (10v-Synflorix vaccine, which covers 10 types of the pneumococcal germ) at 24 months of age. Vaccines will be given by staff from Pasteur Institute Ho Chi Minh City. Your baby will also have a doctor's health check at each study visit.

Questionnaire: At the start of the study you will be asked some general questions about your family and your baby's health. These are simply to help us understand how the vaccines work best. The results will be kept confidential (see below).

Blood tests: Three blood tests will be taken over the six months, by staff from Children's Hospital Number 2. The blood tests are to check the response to the vaccines. If you would prefer, we can put local anesthetic cream on your baby's skin before taking the blood test so that it doesn't hurt as much. The amount of blood taken will be 3.5 or 7.5mls at 18 and 24 months of age; and 3.5mls at 19 months of age.

Nose swabs: Two nose swabs will be taken during the study, at 18 and 24 months of age. The nose swabs are to see if the vaccine will help stop the spread of the pneumococcus from child to child. This will involve putting a cotton wool swab (like a cotton bud) into the baby's nose for a couple of seconds. This may make the baby sneeze and possibly cry briefly – it tickles quite a lot, but doesn't really hurt.

Hospital record review: If your baby becomes unwell during the study, the staff may need to look at your child's medical records.

Are there any risks?

The vaccines we are using are licensed many countries. As with all vaccines there is likely to be some pain felt, and there is a small risk of soreness and redness where the vaccine was given. We will check the babies to make sure they don't have any unexpected reactions. We also have a study doctor who will be keeping a record of any serious illnesses that are unlikely to occur during the study.

Confidentiality

All information collected in this study will remain confidential and will be used for research purposes only. All information will be kept secure. Your baby will be given an identification number at the start of the study. Any information collected will use this number and will not include your baby's name. The samples we collect will be sent to overseas laboratories to have further tests. These laboratories will not be given your child's name. We will ask your permission if it is alright for your baby's blood and nose swab samples to be stored indefinitely for other similar tests in the future. This would help us to perform any new pneumococcal test that may be developed in the

future. The results of the study will be published in scientific journals and presented at conferences. There will never be details published that would identify your baby.

Voluntary Participation and Withdrawal from the Study

Your baby does not have to take part in the study. Your baby will get the best treatment available and the full attention of the health staff even if they do not participate. You are free to withdraw your baby from the study at any point. This will not affect any of your baby's further health care treatment and there will be no harmful consequences for your baby. If your baby has not had all their pneumococcal vaccines they may not be fully protected against the pneumococcal germs which most commonly affect infants. However, they will still gain some protection from the doses of vaccine received.

Compensation

We will pay 200,000VND towards the transport cost for coming to the clinic for each study visit. If your baby becomes ill or injured as a result of taking part in this clinical study, medical treatment will be provided.

Ethical Approval

This study has been approved by the People's Committee of Ho Chi Minh City. This study has also been approved by the Vietnam Ministry of Health Ethics Committee and the Menzies School of Health Research Ethics Committee, Australia. The ethics committees make sure that the study is being done in the best and safest way. If you have any concerns or complaints regarding the conduct of the research project you are invited to contact:

Vietnam Ministry of Health
Ethics Committee
Phone: 04 62732156

OR

Human Research Ethics Committee of the NT
Department of Health and Menzies School of Health Research
PO Box 41096, Casuarina, NT 0811, Australia
Phone: 61 8 8922 7922
Email: ethics@menzies.edu.au

How is the study funded?

The funding to perform the study is from the National Health and Medical Research Council, Australia and the Bill & Melinda Gates Foundation.

Your Right to Ask Questions

Please feel free to contact us if you have any questions or concerns.

If you have any questions regarding the study activities, please phone:

If you have any questions regarding adverse events, please phone:

Commune Health Centre Number:

menzies
school of health research

PO Box 41096, Casuarina NT 0811, Australia
John Mathews Building (Bldg 58),
Royal Darwin Hospital Campus, Rocklands Dve, Casuarina NT 0810
Ph: 08 8922 8196 | Fax: 08 8927 5187 | Web: www.menzies.edu.au
ABN: 70 413 542 847

discovery for a healthy tomorrow

CONSENT FORM

This means you can say NO

Screening Number: |___|___|___|___|

Participant ID: |___|___|___|___|

Date: __ __ /__ __ /__ __
dd / mm / yy

Principal Investigators:

Assoc. Prof. Tran Ngoc Huu
Prof. Edward Kim Mulholland

Research Partners:

Pasteur Institute, Ho Chi Minh City
Menzies School of Health Research

This form is to record if you agree for your infant to take part in the **“Evaluation of Different Infant Vaccination Schedules Incorporating Pneumococcal Vaccination”**. You should only sign this form if you are happy that the information about the study has been clearly explained to you, you have received enough information about the study and you have had all your questions answered satisfactorily.

Please record the name of the person you have spoken to about the study:

____

By agreeing for your infant to take part in the study, you understand that:

- You are free to withdraw your child from the study at any time without having to give a reason;
- Your child will be vaccinated against all the diseases that are covered by the standard vaccines used in Vietnam, although these vaccines may be given at different times;
- If your child becomes sick, their hospital records will be reviewed by the study doctor or other designated study staff; and
- The samples taken in this study will be sent to overseas laboratories to test vaccine responses and carriage of bacteria

menzies
school of health research

PO Box 41096, Casuarina NT 0811, Australia
John Mathews Building (Bldg 58),
Royal Darwin Hospital Campus, Rocklands Dve, Casuarina NT 0810
Ph: 08 8922 8196 | Fax: 08 8927 5187 | Web: www.menzies.edu.au
ABN: 70 413 542 847

discovery for a healthy tomorrow

Screening Number: |___|___|___|___|

Participant ID: |___|___|___|___|

Date: __ __ /__ __ /__ __

Consent:

❑ YES, I agree for my infant to take part in this study.

❑ NO, I do not agree for my infant to take part in this study.

Use of samples:

❑ YES, you may indefinitely store my unused identified samples for future work in the same general area of research that has obtained ethics committee approval.

❑ NO, you may **NOT USE** my samples for future research. Destroy my unused samples at the close of the study.

Signed (parent/legal guardian): ____________________________ Date: __ __ /__ __ /__ __
dd / mm / yy

Name of parent/legal guardian: ____________________________ Time: __ __ :__ __
hh : mm

Relationship to infant: ____________________________

Name of infant or baby of: ____________________________

Infant Sex: male / female Infant DOB: __ __ /__ __ /__ __
dd / mm / yy

Signed (study nurse): ____________________________ Date: __ __ /__ __ /__ __
dd / mm / yy

If illiterate: *A literate witness must sign (if possible, this person should be selected by the participant and should have no connection to the research team).*

I have witnessed the accurate reading of the consent form to the parent of the potential participant, and the individual has had the opportunity to ask questions. I confirm that the individual has given consent freely.

Signed (witness): ____________________________ Date: __ __ /__ __ /__ __
dd / mm / yy

Name of witness: ____________________________
