# Supplementary material for: Evaluation of different infant vaccination schedules incorporating pneumococcal vaccination (The Vietnam Pneumococcal Project): protocol of a randomised controlled trial
Source: BMJ Open. 2018 Jun 8;8(6):e019795. doi: 10.1136/bmjopen-2017-019795 (PMC6009474; doi:10.1136/bmjopen-2017-019795)
Supplement: Author's manuscript [file bmjopen-2017-019795.draft_revisions.pdf]

BMJ Open

BMJ Open is committed to open peer review. As part of this commitment we make the peer review history of every article we publish publicly available.

When an article is published we post the peer reviewers' comments and the authors' responses online. We also post the versions of the paper that were used during peer review. These are the versions that the peer review comments apply to.

The versions of the paper that follow are the versions that were submitted during the peer review process. They are not the versions of record or the final published versions. They should not be cited or distributed as the published version of this manuscript.

BMJ Open is an open access journal and the full, final, typeset and author-corrected version of record of the manuscript is available on our site with no access controls, subscription charges or pay-per-view fees (http://bmjopen.bmj.com).

If you have any questions on BMJ Open's open peer review process please email info.bmjopen@bmj.com

Evaluation of different infant vaccination schedules incorporating pneumococcal vaccination (the Vietnam Pneumococcal Project): protocol of a randomised controlled trial

Journal:	*BMJ Open*
Manuscript ID	bmjopen-2017-019795
Article Type:	Protocol
Date Submitted by the Author:	28-Sep-2017

Complete List of Authors:	Temple, Beth; Menzies School of Health Research, Global Health Division; London School of Hygiene and Tropical Medicine, Epidemiology and Population Health<br>Toan, Nguyen; Pasteur Institute of Ho Chi Minh City, Department of Disease Control and Prevention<br>Uyen, Doan ; Pasteur Institute of Ho Chi Minh City, Department of Disease Control and Prevention<br>Balloch, Anne; Murdoch Childrens Research Institute, Pneumococcal Research<br>Bright, Kathryn; Murdoch Childrens Research Institute, Pneumococcal Research<br>Cheung, Yin Bun; Duke-NUS Medical School, Centre for Quantitative Medicine; University of Tampere and Tampere University Hospital, Centre for Child Health Research<br>Licciardi, Paul; Murdoch Childrens Research Institute, Pneumococcal Research; University of Melbourne, Department of Paediatrics<br>Nguyen, Cattram; Murdoch Childrens Research Institute, Pneumococcal Research; University of Melbourne, Department of Paediatrics<br>Phuong, Nguyen; Pasteur Institute of Ho Chi Minh City, Department of Disease Control and Prevention<br>Satzke, Catherine; Murdoch Childrens Research Institute, Pneumococcal Research; University of Melbourne, Department of Paediatrics<br>Smith-Vaughan, Heidi; Menzies School of Health Research, Child Health Division<br>Vu, Thi Que Huong; Pasteur Institute of Ho Chi Minh City, Microbiology and Immunology<br>Huu, Tran; Pasteur Institute of Ho Chi Minh City, Department of Disease Control and Prevention<br>Muholland , Kim; London School of Hygiene and Tropical Medicine, Epidemiology and Population Health; Murdoch Childrens Research Institute, Pneumococcal Research

Keywords:	Clinical trials < THERAPEUTICS, Paediatric infectious disease & immunisation < PAEDIATRICS, MICROBIOLOGY, Epidemiology < INFECTIOUS DISEASES

For peer review only - http://bmjopen.bmj.com/site/about/guidelines.xhtml

For peer review only

Title
Evaluation of different infant vaccination schedules incorporating pneumococcal vaccination (The Vietnam Pneumococcal Project): protocol of a randomised controlled trial

Corresponding author
Beth Temple
Menzies School of Health Research
PO Box 41096
Casuarina NT 0811
Australia
+61 8 8946 8600
beth.temple@menzies.edu.au

For peer review only

Co-authors

TEMPLE, Beth[1, 2]
TOAN, Nguyen Trong[4]
UYEN, Doan Y[4]
BALLOCH, Anne[3]
BRIGHT, Kathryn[3]
CHEUNG, Yin Bun[5, 6]
LICCIARDI, Paul[3, 7]
NGUYEN, Cattram Duong[3, 7]
PHUONG, Nguyen Thi Minh[4]
SATZKE, Catherine[3, 7]
SMITH-VAUGHAN, Heidi[8]
VU, Thi Que Huong[9]
HUU, Tran Ngoc[4]
MULHOLLAND, Edward Kim[2, 3]

[1]Global Health, Menzies School of Health Research, Darwin, Australia
[2]Epidemiology and Population Health, London School of Hygiene & Tropical Medicine, London, UK
[3]Pneumococcal Research, Murdoch Childrens Research Institute, Melbourne, Australia
[4]Department of Disease Control and Prevention, Pasteur Institute of Ho Chi Minh City, Ho Chi Minh City, Vietnam
[5]Centre for Quantitative Medicine, Duke-NUS Medical School, Singapore, Singapore
[6]Centre for Child Health Research, University of Tampere and Tampere University Hospital, Tampere, Finland
[7]Department of Paediatrics, University of Melbourne, Melbourne, Australia
[8]Child Health, Menzies School of Health Research, Darwin, Australia
[9]Microbiology and Immunology, Pasteur Institute of Ho Chi Minh City, Ho Chi Minh City, Vietnam

Word count: 6,081 words

ABSTRACT

Introduction: The World Health Organization (WHO) recommends the use of pneumococcal conjugate vaccine (PCV) as a priority. However there are many countries yet to introduce PCV, especially in Asia. This trial aims to evaluate different PCV schedules and to provide a head-to-head comparison of PCV10 and PCV13, in order to generate evidence to assist with decisions regarding PCV introduction.

Methods and analysis: This randomised, single-blind controlled trial will recruit 1200 infants aged between 60 and 74 days to one of six PCV schedules (PCV10 at 2, 3, 4 and 9 months, 2, 3 and 4 months, 2, 4 and 9 months, or 2 and 6 months; PCV13 at 2, 4 and 9 months; and unvaccinated controls that receive PCV10 and 18 and 24 months), along with an additional control group of 200 children aged 18 months (that receive PCV10 at 24 months), and follow them up until 24 months of age. The primary outcome is the post-primary series immunogenicity, expressed as the proportions of participants with serotype-specific antibody levels ≥0.35μg/mLfor each serotype in PCV10. Secondary outcome measures are additional immunogenicity measures (geometric mean concentrations of antibody, opsonophagocytic assays, and memory B cell assays) and nasopharyngeal carriage of *Streptococcus pneumoniae* and *Haemophilus influenzae*.

Ethics and dissemination: Ethical approval has been obtained from the Human Research Ethics Committee of the Northern Territory Department of Health and Menzies School of Health Research (EC00153) and the Vietnam Ministry of Health Ethics Committee. The results, interpretation and conclusions will be presented to parents and guardians, at national and international conferences, and published in peer-reviewed open access journals.

Trial registration details: NCT01953510

Strengths and limitations of this study

- This study is specifically designed to address two independent questions within a single study: which schedule to use for the provision of PCV, and which PCV to use.
- This study includes a head-to-head comparison of the two licensed PCVs, allowing a direct assessment of their relative immunogenicity and impact on nasopharyngeal carriage.
- The primary outcome is the criteria used for the licensing and varying of PCV schedules.
- Limitations of this study include vulnerability of the power of the secondary nasopharyngeal carriage outcomes to variations in carriage rates.

For peer review only

ADMINISTRATIVE INFORMATON

1 Title
Evaluation of different infant vaccination schedules incorporating pneumococcal vaccination (the Vietnam Pneumococcal Project).

2a Trial registration
ClinicalTrials.gov: NCT01953510

2b Trial registration - data set

Data category	Information
Primary registry and trial identifying number	ClinicalTrials.gov NCT01953510
Date of registration in primary registry	25 September 2013
Secondary identifying numbers	09/19, 10PN-PD-DIT-079
Source(s) of monetary or material support	National Health and Medical Research Council, Australia<br>Bill & Melinda Gates Foundation<br>GlaxoSmithKline Biologicals SA
Primary sponsor	Murdoch Childrens Research Institute, Australia
Contact for public queries	kim.mulholland@lshtm.ac.uk
Contact for scientific queries	kim.mulholland@lshtm.ac.uk
Public title	Trial of pneumococcal vaccine schedules in Ho Chi Minh City, Vietnam
Scientific title	Evaluation of different infant vaccination schedules incorporating pneumococcal vaccination (Vietnam Pneumococcal Project)
Countries of recruitment	Vietnam
Health condition(s) or problem(s) studied	Pneumococcal vaccination
Intervention(s)	Active Comparator A: PCV10 administered at 2, 3, 4 and 9 months of age (3+1)
	Experimental B: PCV10 administered at 2, 3 and 4 months of age (3+0)
	Experimental C: PCV10 administered at 2, 4 and 9 months of age (2+1)
	Experimental D: PCV10 administered at 2 and 6 months of age (2 dose)
	Experimental E: PCV13 administered at 2, 4 and 9 months of age (2+1 PCV13)
	No intervention F: No infant PCV vaccination; PCV10 administered at 18 and 24 months of age (Control F)
	No intervention G: Recruited at 18 months of age, non-randomised; PCV10 administered at 24 months of age (Control G)
Key inclusion and exclusion criteria	Inclusion:<br>• Aged between 2 months and 2 months plus 2 weeks

For peer review only

	(Arms A-F) or aged between 18m and 18m plus 4 weeks (Arm G)<br>• No significant maternal or perinatal history<br>• Born at or after 36 weeks gestation<br>• Written and signed informed consent from parent/legal guardian<br>• Lives within approximately 30 minutes of the commune health centre<br>• Family anticipates living in the study area for the next 22 months (Arms A-F) or 6 months (Arm G)<br>• Has received three doses of either Quinvaxem or Infanrix-hexa in infancy (Arm G)<br>•
	Exclusion:<br>• Known allergy to any component of the vaccine<br>• Allergic reaction or anaphylactic reaction to any previous vaccine<br>• Known immunodeficiency disorder<br>• Known HIV-infected mother<br>• Known thrombocytopenia or coagulation disorder<br>• On immunosuppressive medication<br>• Administration or planned administration of any immunoglobulin or blood product since birth<br>• Severe birth defect requiring ongoing medical care<br>• Chronic or progressive disease<br>• Seizure disorder<br>• History of invasive pneumococcal, meningococcal or *Haemophilus influenzae* type b diseases, or tetanus, measles, pertussis or diphtheria infections<br>• Receipt of any 2 month vaccines through the EPI program (Arms A-F), or receipt of PCV (Arm G);<br>Family plans on giving the infant the Quinvaxem (DTP-Hib-HBV) or OPV vaccines (Arms A-F)
Study type	Interventional, randomised, parallel group, open label phase II/III trial (Arms A-F). Non-randomised (Arm G). Outcomes assessors (laboratory) blinded. Purpose: prevention.
Date of first enrolment	30 September 2013
Target sample size	1400
Recruitment status	Active, not recruiting
Primary outcome	Proportion of children with IgG antibody concentration ≥0.35μg/mL for individual pneumococcal serotypes, four weeks post-primary series, measured by ELISA
Key secondary outcomes	Geometric mean concentration (GMC) of serotype-specific IgG, four weeks post-primary series, measured by ELISA
	Proportion of children with IgG antibody concentration ≥0.35μg/mL and GMCs, four weeks post-booster, measured by ELISA
	Proportion of children with serotype-specific opsonisation indices ≥8, four weeks post-primary series and four weeks post-booster, measured by opsonophagocytic assay
	Median number of serotype-specific antibody secreting memory B cells, four weeks post-booster and at 18 months of

For peer review only

	age, measured by ELISPOT
	Proportion of children carrying pneumococcus (any pneumococci, capsular pneumococci, or vaccine-type pneumococci) in the nasopharynx at 12 months of age, measured by culture and latex agglutination serotyping
	Proportion of children carrying NTHi in the nasopharynx at 12 months of age, measured by culture and PCR

3 Protocol version
Protocol version 10.0 dated 3 June 2015 with Letter of Amendment Number 1 dated 1 September 2016

Revision chronology
Original: Version 3.1, 5 June 2013
First amendment: Version 5.0, 21 April 2014. Main reason for amendment: the Vietnam Ministry of Health (MOH) does not permit the co-administration of measles vaccine and *Infanrix-hexa* vaccine, which was scheduled at 9 months of age in Arms C and E. An additional visit at 9.5 months of age was added for these groups, for receipt of PCV and *Infanrix-hexa*.
Second amendment: Version 7.0, 8 December 2014. Main reason for amendment was that additional funding was secured to: extend the follow up of all participants from 18 to 24 months of age; evaluate a single dose of PCV10 at 18 months of age; and recruit an additional control group at 18 months of age (Arm G) to provide a comparator for the original control group (Arm F). Version 7.0 was never implemented (see below).
Third amendment: Version 9.0, 4 March 2015. Main reason for amendment: to incorporate minor clarifications to version 7.0 requested during review by MOH. These changes did not affect participant recruitment or follow-up and the version number was only changed at the request of MOH.
Fourth amendment: Version 10.0, 3 June 2015. Main reason for amendment: to incorporate additional minor clarifications to version 9.0 requested during review by the Vietnam Ministry of Health. These changes did not affect participant recruitment or follow-up and the version number was only changed at the request of MOH.

4 Funding
The trial is funded by the National Health and Medical Research Council of Australia (grant number 566792) and the Bill & Melinda Gates Foundation (grant number OPP1116833). The doses of PCV10 and funding for the opsonophagocytic assays are provided by GlaxoSmithKline Biologicals SA (GSK).

5 Roles and responsibilities
5a Protocol contributors
TEMPLE, Beth[1, 2] was involved with the study design, led the funding and ethics applications, and has been involved in the day-to-day management of the trial and data analysis.
TOAN, Nguyen Trong[4] advised on the study design and location, was involved in the approval processes in Vietnam, and has been involved in the day-to-day management and implementation of the trial.
UYEN, Doan Y[4] advised on the study design and location and has been involved in the day-to-day implementation of the trial.
BALLOCH, Anne[3] advised on the study design, assisted with the funding applications, and advised on and provided oversight of the immunology laboratory procedures.

For peer review only - http://bmjopen.bmj.com/site/about/guidelines.xhtml

BRIGHT, Kathryn[3] advised on the study design and location and has been responsible for the day-to-day management and implementation of the trial.
CHEUNG, Yin Bun[5, 6] advised on the study design and funding applications, especially the statistical aspects of the trial.
LICCIARDI, Paul[3, 7] advised on the study design, assisted with the funding applications, and advised on and provided oversight of the immunology laboratory procedures.
NGUYEN, Cattram Duong[3, 7] advised on the study design and statistical analysis plan.
PHUONG, Nguyen Thi Minh[4] advised on the study design and location, was involved in the approval processes in Vietnam, and has been involved in the day-to-day management of the trial.
SATZKE, Catherine[3, 7] advised on the study design, assisted with the funding applications, and advised on and provided oversight of the microbiology laboratory procedures.
SMITH-VAUGHAN, Heidi[8] advised on the study design, assisted with the funding applications, and advised on and provided oversight of the microbiology laboratory procedures.
VU, Thi Que Huong[9] advised on the study design and advised on and provided oversight of the laboratory procedures at Pasteur.
HUU, Tran Ngoc[4] advised on the study design and location, undertook consultations, was involved in the approval processes in Vietnam, and has had overall responsibility for the conduct of the trial in Vietnam as Site Principal Investigator.
MULHOLLAND, Edward Kim[2, 3] conceived the study, undertook consultations, provided oversight for the funding and ethics applications, provided oversight for the conduct of the trial and data analysis, and has had overall responsibility for all aspects of the trial as the Principal Investigator.

[1]Global Health, Menzies School of Health Research, Darwin, Australia
[2]Epidemiology and Population Health, London School of Hygiene & Tropical Medicine, London, UK
[3]Pneumococcal Research, Murdoch Childrens Research Institute, Melbourne, Australia
[4]Department of Disease Control and Prevention, Pasteur Institute of Ho Chi Minh City, Ho Chi Minh City, Vietnam
[5]Centre for Quantitative Medicine, Duke-NUS Medical School, Singapore, Singapore
[6]Centre for Child Health Research, University of Tampere and Tampere University Hospital, Tampere, Finland
[7]Department of Paediatrics, University of Melbourne, Melbourne, Australia
[8]Child Health, Menzies School of Health Research, Darwin, Australia
[9]Microbiology and Immunology, Pasteur Institute of Ho Chi Minh City, Ho Chi Minh City, Vietnam

5b Sponsor contact information
Trial Sponsor: Murdoch Childrens Research Institute, Royal Children's Hospital, Flemington Road, Parkville, Victoria 3052, Australia
Telephone: +61 3 8341 6200
Contact name: Professor Kim Mulholland

5c Sponsor and funder
GSK was consulted during the design of the trial. None of the funders have any role in the trial conduct, trial management, laboratory tests, or data analyses.

For peer review only

INTRODUCTION

6a Background and rationale

Streptococcus pneumoniae (pneumococcus) remains a leading vaccine preventable cause of serious infection in young children, despite the availability of effective vaccines. The first infant pneumococcal vaccine, the 7-valent pneumococcal conjugate vaccine (PCV7), was licensed in the United States in the year 2000. Introduction of PCV7 has been associated with dramatic reductions in pneumococcal disease.[1-3] However, geographical variation in serotype distribution[4-7] and an increase in invasive pneumococcal disease (IPD) caused by non-PCV7 serotypes following vaccine introduction[8] necessitated the development of higher valency PCVs.

There are currently two licensed PCVs: PCV10, a 10-valent pneumococcal vaccine that uses non-typeable *Haemophilus influenzae* (NTHi) protein D as a carrier protein for eight of the ten serotypes (*Synflorix*™, PHiD-CV, GSK); and PCV13, a 13-valent pneumococcal CRM_{197} conjugate vaccine (*Prevnar-13*™*/Prevenar-13*™, Pfizer). Both have been shown to be non-inferior to PCV7.[9-11] Despite the availability of both PCV10 and PCV13 for several years, there have been no published studies to date directly comparing their post-primary series immunogenicity or impact on nasopharyngeal (NP) carriage.

The cost of PCVs is a major barrier to vaccine introduction in low to middle-income countries; therefore investigation of alternative schedules with a reduced number of doses is of great importance. The uptake of PCV introduction in Asia has been particularly slow. Three PCV schedules are currently in routine use around the world: a 3+1 schedule (a three-dose primary series followed by a booster dose in the second year of life); a 3+0 schedule (a three-dose primary series without a booster dose); and a 2+1 schedule (a two-dose primary series followed by a booster dose in the second year of life). Data from periods of PCV7 shortage in the United States show high vaccine effectiveness of a two-dose primary series against invasive pneumococcal disease (IPD),[12 13] and trial data of CRM_{197}-conjugated PCVs show comparable immunogenicity following a two- or three-dose primary series, although antibody levels to serotypes 6B and 23F tend to be lower after two doses.[14 15] Trials of PCV10 and PCV13 also support the use of a two-dose primary series. A trial of PCV10 in Europe directly comparing the immunogenicity of a two- and three-dose

For peer review only - http://bmjopen.bmj.com/site/about/guidelines.xhtml

primary series showed a similar proportion of participants achieving protective antibody levels (≥0.2μg/mL) for all ten serotypes.[16] In a trial of PCV13 in Mexico, over 93% of participants achieved protective antibody levels (≥0.35μg/mL) for most of the 13 serotypes following two doses, with the exception of serotypes 6B and 23F.[17] Four trials in Europe directly comparing PCV13 and PCV7 responses showed comparable immune responses between the vaccines following two doses.[18]

In developing countries, a 2+1 schedule with an earlier booster dose may be advantageous. This modified schedule would likely increase compliance, would provide full immunisation closer to the peak incidence of pneumococcal disease, and could enable the booster dose to coincide with measles vaccination. Alternatively, a further reduced PCV schedule with only two doses may be optimal for pneumococcal vaccination. Our previous trial in Fiji showed that protective antibody levels were reached for five of the seven serotypes following a single dose of PCV7 at 14 weeks of age.[15] Furthermore, a booster dose of the 23-valent pneumococcal polysaccharide vaccine at 12 months of age was more immunogenic following a single dose primary series of PCV7 compared with a two or three dose primary series for four serotypes, and comparable for the other three serotypes.[19] A trial of PCV9 from South Africa also showed that one dose at six weeks of age elicited a significant response for seven serotypes,[20] and modelling data from the US suggest that a single dose of PCV could prevent up to 62% of IPD.[21]

Carriage of pneumococci in the nasopharynx is commonly a prerequisite for IPD, and is the usual means of transmission of the bacteria. The herd effect of pneumococcal vaccination is mediated by the impact on NP carriage.[22] Vaccination with PCVs generally results in a decrease in vaccine type (VT) pneumococcal carriage, which is most commonly observed after a booster dose and often accompanied by a compensatory increase in non-VT carriage.[22-26] Little is known about the effect of different PCV schedules on carriage. A trial from the Netherlands showed that a two-dose primary series with or without a booster reduced VT carriage at 12 months of age compared with controls.[27] VT carriage was further reduced at 18 months in the group that received the booster dose, compared with the group that did not receive the booster, although this difference did not persist at 24 months of age. Similarly, our trial in Fiji showed that a two or three dose primary series with or without a booster reduced VT carriage at 12 months of age compared with controls, but no difference was seen at 17 months of age (F Russell, personal communication).

For peer review only

It has been hypothesised that the Protein D carrier in PCV10 may result in an impact on *H. influenzae* carriage. A recent review of the impact of Protein D-containing PCVs on NTHi carriage concludes that any such impact is likely to be small and transient, although changes in the density of carriage are yet to be evaluated.[28] Two large phase III trials (POET trial of an 11-valent PCV and COMPAS trial of PCV10) showed trends towards a reduction in NTHi carriage following a booster dose of PCV, along with a trial of PCV10 in toddlers in Kenya; but other trials conducted in Finland, the Netherlands and the Czech Republic showed no impact of PCV10 on NTHi carriage.

This trial includes six infant vaccination schedules: four different PCV10 schedules (Arm A, a 3+1 schedule at 2, 3, 4 and 9 months of age; Arm B, a 3+0 schedule at 2, 3 and 4 months; Arm C, a 2+1 schedule at 2, 4 and 9 months; and Arm D, a 2-dose schedule at 2 and 6 months); a 2+1 PCV13 schedule at 2, 4 and 9 months (Arm E); and a control group that receives two doses of PCV10 at 18 and 24 months (Arm F). In response to more recent interest in schedules with only one or two doses of PCV, which may be sufficient to maintain herd immunity at the population level, an additional control group is recruited at 18 months of age for comparison with the initial control group (Arm G).

6b Explanation for choice of comparators

There was no PCV licensed in Vietnam at the time the protocol was finalised in 2013. The inclusion of control groups that receive no infant doses of PCV is therefore justified. Control group participants recruited in infancy receive two doses of PCV10, at 18 and 24 months of age. Control group participants recruited at 18 months of age receive a single dose of PCV10 at 24 months of age. Intervention group participants receive at least two doses of PCV in the first year of life. All participants receive pneumococcal immunisation that is likely to be effective and is not otherwise available in Vietnam. The specific regimens to be evaluated are based on likely future global recommendations and to directly compare the two licensed PCVs.

Both PCV10 and PCV13 have been shown to be non-inferior to PCV7 for the serotypes common to both vaccines, and to have the potential to provide protection against the additional serotypes included.[9-11] For both vaccines the most common

For peer review only - http://bmjopen.bmj.com/site/about/guidelines.xhtml

adverse reactions are redness at the injection site and irritability, which are common following administration of other vaccines. Other adverse reactions may include: drowsiness; temporary loss of appetite; pain, redness or swelling at the injection site; and fever. Such reactions are usually temporary.

7 Objectives

This trial has been designed to answer two independent questions concurrently, relating to the evaluation of different schedules incorporating PCV10 and the comparison of PCV10 and PCV13:

1) What is the optimal schedule for provision of EPI vaccines with the incorporation of PCV10; and
2) How do the responses to vaccination with PCV10 or PCV13 compare?

The primary endpoint for both study questions is the post-primary series immunogenicity. For this endpoint data from Arms A and B are combined, as they receive an identical three-dose primary series (see Table 1 for a detailed description of the trial arms). The primary analysis for each study question is to assess non-inferiority of the post-primary series immunogenicity (in terms of the proportion of participants achieving protective levels of serotype-specific IgG of ≥0.35μg/mL), using Arms A+B as the comparator (see below for details). Non-inferiority is assessed for each of the ten serotypes in PCV10, and an overall conclusion of non-inferiority drawn if found for at least seven of the ten serotypes.

1) What is the optimal schedule for provision of EPI vaccines with the incorporation of PCV10?

Primary objective

The primary objective is to compare a 2+1 schedule at 2, 4 and 9 months of age with a 3+1 schedule at 2, 3, 4 and 9 months of age. The primary hypothesis is that the proportion of participants with protective levels of antibody is non-inferior following a two-dose primary series (Arm C) compared with a three-dose primary series (Arms A+B). The schedules will also be compared in relation to: the IgG levels and opsonophagocytosis post-primary series; the proportion of participants with protective levels of antibody, the IgG levels and opsonophagocytosis post-booster; the memory B cell responses; the impact on nasopharyngeal (NP) carriage rates and density of bacteria of interest; and the immunogenicity of the co-administered vaccines.

For peer review only

Key secondary objectives

- To investigate an experimental two-dose schedule at 2 and 6 months of age (Arm D), compared with a 3+1 schedule (Arm A+/-B) and a 2+1 schedule (Arm C);
- To assess the impact of a booster dose on NP carriage of pneumococcus and NTHi, comparing a 3+1 schedule (Arm A) with a 3+0 schedule (Arm B) and with unvaccinated controls (Arm F); and
- To evaluate a single dose of PCV10 at 18 months of age (Arm F), compared with unvaccinated controls (Arm G).

2) How do the responses to vaccination with PCV10 or PCV13 compare?

Primary objective

The primary objective is to compare a PCV13 schedule at 2, 4 and 9 months of age with a PCV10 schedule at 2, 3, 4 and 9 months of age. The primary hypothesis is that the immunogenicity is non-inferior following a two-dose primary series of PCV13 (Arm E) compared with a three-dose primary series of PCV10 (Arms A+B). The schedules will also be compared in relation to: the IgG levels and opsonophagocytosis post-primary series; the proportion of participants with protective levels of antibody, the IgG levels and opsonophagocytosis post-booster; the memory B cell responses; the impact on nasopharyngeal (NP) carriage rates and density of bacteria of interest; and the immunogenicity of the co-administered vaccines.

Key secondary objectives

- To compare PCV10 (Arm C) and PCV13 (Arm E) in a 2+1 schedule at 2, 4 and 9 months of age; and
- To compare the responses to a single dose of PCV10 (Arm D) and PCV13 (Arm E).

8 Trial design

The Vietnam Pneumococcal Project is a single-blind, open-label, randomized controlled phase II/III non-inferiority trial to investigate simplified childhood

For peer review only

vaccination schedules that are more appropriate for developing country use. This is a seven-arm trial that includes six different infant vaccination schedules (Arm A-F) and an additional control group (Arm G) recruited at 18 months of age (Table 1).

METHODS: PARTICIPANTS, INTERVENTIONS AND OUTCOMES

9 Study setting

PCV introduction in Asia has been slow, in part due to a lack of local or regional data on the effect of PCV. We selected the Southeast Asian country of Vietnam as the location for the trial as a country with a strong health system, a track record of conducting relevant clinical trials, and a Government with strong interest both in the trial and in introducing PCV in the near future. Furthermore, trial results from Vietnam are likely to be considered as applicable to other countries in the region. This is the first trial involving infants to take place within Ho Chi Minh City, the largest city in Vietnam. The trial is conducted in two districts, District 4 and District 7. Districts are divided into communes, each of which has a health centre that provides preventive health services including EPI immunizations, along with some primary health care services. The study is conducted in one commune health centre in each district, with participants drawn from the surrounding communes within that district.

10 Eligibility criteria

Inclusion criteria

Subjects must meet all of the following inclusion criteria in order to be eligible to participate: aged between 2 months and 2 months plus 2 weeks (Arms A-F) or aged between 18 months and 18 months plus 4 weeks (Arm G); no significant maternal or perinatal history; born at or after 36 weeks gestation; written informed consent from the parent/legal guardian; lives within approximately 30 minutes of the commune health centre; anticipates living in the study area for the next 22 months (Arms A-F) or 6 months (Arm G); and received 3 doses of either *Quinvaxem* (DTP-Hib-HBV) or *Infanrix-hexa* (DTP-Hib-HBV-IPV) in infancy (Arm G only).

Exclusion criteria

Subjects meeting any of the following exclusion criteria at baseline will be excluded from study participation: known allergy to any component of the vaccine; allergic or anaphylactic reaction to any previous vaccine; known immunodeficiency disorder;

For peer review only - http://bmjopen.bmj.com/site/about/guidelines.xhtml

known HIV-infected mother; known thrombocytopenia or coagulation disorder; on immunosuppressive medication; administration or planned administration of any immunoglobulin or blood product since birth; severe birth defect requiring ongoing medical care; chronic or progressive disease; seizure disorder; history of invasive pneumococcal, meningococcal or *H. influenzae* type b diseases, or tetanus, measles, pertussis or diphtheria infections; receipt of any 2 month vaccines through the EPI program (Arms A-F), or receipt of PCV (Arm G); or family plans on giving the infant *Quinvaxem* (Arms A-F).

11 Interventions

PCV schedules

Eligible participants recruited in infancy are randomised to one of six different vaccination schedules (Table 1). Participants randomised to Arms A-D receive PCV10 in a: 3+1 schedule at 2, 3, 4 and 9 months of age; a 3+0 schedule at 2, 3 and 4 months of age; a 2+1 schedule at 2, 4 and 9 months of age; or a two-dose schedule at 2 and 6 months of age, respectively. Participants randomised to Arm E receive PCV13 in a 2+1 schedule at 2, 4 and 9 months of age. Control group participants receive PCV10 at 18 and 24 months of age if randomised to Arm F, or PCV10 at 24 months of age if recruited to Arm G at 18 months of age. PCV is administered by intramuscular injection into the anterolateral thigh in children less than 18 months old and in the deltoid muscle of the arm in children aged 18 months and over. All vaccinations are performed by nurses specifically trained in infant vaccine administration.

PCV10

PCV10 (*Synflorix*) is a 10-valent pneumococcal polysaccharide conjugate vaccine using Protein D (a highly conserved surface protein from NTHi) as the main carrier protein. PCV10 is presented as a turbid white suspension in a two-dose vial. One dose consists of 0.5mL of the liquid vaccine, containing 1µg of pneumococcal polysaccharide from serotypes 1, 5, 6B, 7F, 9V, 14 and 23F and 3µg of pneumococcal polysaccharide from serotypes 4, 18C and 19F. Serotypes 1, 4, 5, 6B, 7F, 9V, 14 and 23F are conjugated to Protein D; serotype 18C is conjugated to tetanus toxoid carrier protein; and serotype 19F is conjugated to diphtheria toxoid carrier protein.

For peer review only - http://bmjopen.bmj.com/site/about/guidelines.xhtml

PCV13

PCV13 (*Prevnar-13*) is a 13-valent pneumococcal polysaccharide conjugate vaccine using non-toxic diphtheria CRM_{197} carrier protein. PCV13 is presented as a 0.5mL suspension in a single-dose pre-filled syringe. One dose contains approximately 2.2µg of pneumococcal polysaccharide from serotypes 1, 3, 4, 5, 6A, 7F, 9V, 14, 18C, 19A, 19F and 23F and 4.4µg of pneumococcal polysaccharide from serotype 6B.

11b Criteria for discontinuing or modifying allocated interventions

There is no modification of doses for participants in this study. If a participant has an allergic or anaphylactic response to vaccination they will be withdrawn from the study. Participants may also be withdrawn voluntarily by the parent/legal guardian at any time, or by the study staff if they refuse any further study procedures or develop any of the exclusion criteria during the course of the study.

11c Strategies to improve and monitor adherence

Scheduled visit dates are noted on a health record card kept by the parent. If a participant does not attend a scheduled visit, a reminder phone call is made from the study clinic. If the participant cannot be contacted directly, their local Commune Health Centre is contacted for further follow up by phone or by home visit.

11d Relevant concomitant care

Participants receive four doses of *Infanrix-hexa*, which is only available on the private market, along with routine measles immunisation at 9 months of age and measles-rubella immunisation at 18 months of age. Participants allocated to one of the 2+1 vaccination schedules (Arms C and E) receive measles at 9 months of age and receive PCV and *Infanrix-hexa* two weeks later. Other vaccinations are permitted in this study with a two-week interval from study vaccines, with the exception of *Quinvaxem* in Arms A-F. Other medications are also permitted, with the exception of immunosuppressive medication and medications listed as contraindicated to the study vaccines.

12 Outcomes

Primary outcome measure

The primary outcome measure is the concentration of serotype-specific IgG for the ten serotypes common to both PCV10 and PCV13, assessed four weeks post-

For peer review only

primary series and measured using a modified 3rd generation standardized ELISA.[29] Primary comparisons between arms are made in terms of the proportion of children with antibody concentration ≥0.35μg/mL for individual serotypes. The cut-off of 0.35μg/mL was determined as a result of a pooled analysis of data from efficacy trials,[30] and is used as the basis for non-inferiority assessments for the approval of new PCVs.[31-33]

Secondary immunogenicity outcome measures

- Serotype-specific IgG antibody concentrations for all PCV13 serotypes are measured by ELISA from all blood samples (Table 1) and are summarised in terms of both the proportion of children with antibody concentration ≥0.35μg/mL and the Geometric Mean Concentration (GMC).
- Opsonisation indices (OI) for all PCV13 serotypes are measured by opsonophagocytic assay (OPA)[34] for 100 participants per intervention group (Arms A-E) four weeks post-primary series and four weeks post-booster, and are summarised in terms of the proportion of participants with OI ≥8 and the Geometric Mean Titre (GMT).
- Polysaccharide specific memory B cells for serotypes 1, 5, 6B, 14, 18C, 19A and 23F are enumerated by ELISPOT[34] for 50 participants per intervention group (Arms A-E) post-booster and at 18 months of age, and for 100 participants per control group (Arms F and G) at 18 and 24 months of age. The results are summarised as the median number of antibody secreting cells.

Secondary nasopharyngeal carriage outcome measures

- NP carriage of pneumococcal serotypes is measured by traditional culture (colonial morphology, α-haemolysis, the optochin test and *lytA* PCR where indicated)[35] and latex agglutination using type-specific antisera at 2, 6, 9 and 12 months of age in all groups and at 18 and 24 months of age in the control groups (Arms F and G). NP carriage and density of pneumococcal serotypes are measured by quantitative real-time PCR (qPCR) targeting *lytA* and microarray at 18 and 24 months of age.[36 37] Overall, capsular, vaccine-type and serotype-specific carriage rates are described. The antimicrobial resistance of pneumococcal isolates is determined at 12 months of age by CLSI disk diffusion, for oxacillin, erythromycin, trimethoprim/sulphamethoxazole, ofloxacin, clindamycin, vancomycin, tetracycline, and chloramphenicol. E-tests are

For peer review only - http://bmjopen.bmj.com/site/about/guidelines.xhtml

conducted for penicillin, ceftriaxone, and vancomycin where indicated, and CLSI breakpoints applied.

- NP carriage of *H. influenzae* is measured by traditional culture (colonial morphology, X and V dependence, *SiaT* PCR for discrimination from *H. haemolyticus*, and the Phadebact® Haemophilus coagglutination test) at 12 months of age in all groups, at 6 and 9 months of age in Arms A and C, and from all swabs in the control groups (Arms F and G). Overall density of *H. influenzae* carriage is measured by qPCR targeting *hpd* and *SiaT* diagnostic targets at 18 and 24 months of age.[38 39]

An overview of the procedures for collection, transportation and laboratory analyses of the blood and NP samples can be found in Appendix 2.

For peer review only

For peer review only - http://bmjopen.bmj.com/site/about/guidelines.xhtml

13 Participant timeline

Table 1: Schedule of enrolment, interventions and assessments

**Age (months)**	**2m**	**3m**	**4m**	**5m**	**6m**	**7m**	**9m**	**9.5m**	**10m**	**12m**	**18m**	**19m**	**24m**
ENROLMENT:													
Informed consent	X										X[1]		
Eligibility assessment	X										X[1]		
Allocation	X												
INTERVENTIONS:													
PCV10 - Group A	X	X	X				X						
PCV10 - Group B	X	X	X										
PCV10 - Group C	X		X					X					
PCV10 - Group D	X				X								
PCV13 - Group E	X		X					X					
PCV10 - Group F											X		X
PCV10 - Group G													X
ASSESSMENTS:													
Demographics	X										X[1]		
Household characteristics	X										X[1]		
Nasopharyngeal swab	X				X		X			X	X		X
Blood sample - Group A	X[2]			X			X		X		X[2]		
Blood sample - Group B				X	X		X[2]		X		X[2]		
Blood sample - Group C				X	X[2]		X		X		X[2]		
Blood sample - Group D		X			X	X	X[2]				X[2]		
Blood sample - Group E		X[2]		X			X		X		X[2]		
Blood sample - Group F											X	X	X
Blood sample - Group G											X	X	X
General health	X	X	X	X	X	X	X	X	X	X	X	X	X

[1] Group G only. Any events occurring before 18m do not apply to Group G.

[2] Each participant provides only one of these blood samples

For peer review only

14 Sample size

The target sample size for infant recruitment (Groups A-F) is 1200 with an allocation ratio of 3:3:5:4:5:4, resulting in target group sizes of: A=150, B=150, C=250, D=200, E=250 and F=200. An additional target of 200 children aged 18 months are recruited into Group G. Sample size calculations are based on the primary outcome of post-primary series immunogenicity (proportion of participants with serotype-specific antibody concentrations ≥0.35μg/mL) for each of the two study questions. A non-inferiority margin of 10% difference in absolute risk is deemed clinically significant, as used by regulatory authorities. Non-inferiority is assessed for each of the ten serotypes in PCV10 (comparing Groups A+B with Group C or Group E), and an overall conclusion of non-inferiority is drawn if the alternative hypotheses are accepted for at least seven of the ten serotypes. This sample size provides >99% power for the overall conclusion of non-inferiority with a 5% one-sided type I error rate, estimated by simulation using a tailor-made program written for implementation in Stata with 10,000 replications.[40] Powers for serotype-specific hypotheses range from 83% to >99%, calculated in PASS Software 2002 using the Farrington-Manning (1990) method.[41] Based on findings from our earlier work in Fiji and from data available in the literature,[42-44] the assumed probabilities of antibody concentration ≥0.35μg/mL are: 95% for serotypes 1, 4, 5, 7F, 9V, 14 and 19F; 90% for serotype 18C; 80% for serotype 23F; and 75% for serotype 6B. The within-subject correlation between the multiple binary endpoints is captured by a subject-level variation term with standard deviation 1.7 in a random-effect logistic regression model, and the loss to follow up rate is assumed to be 5% post-primary series and 10% at 12 months of age. The sample size also provides 98% power to detect a difference in post-primary series immunogenicity following two doses of PCV10 or PCV13, defined by a 10% difference in absolute risk based on a Fisher's Exact test (5% two-sided).

Carriage outcomes: The sample size provides 76% and 71% power to detect a difference in NTHi carriage rates at 12 months of age between Groups A and F and Groups A and B, respectively, and 64% and 59% power to detect a difference in vaccine-type pneumococcal carriage rates between Groups A and F and Groups A and B, respectively. Difference in carriage is defined by a relative risk of 0.6. The calculations were based on Fisher's Exact tests (5% one-sided), assuming carriage rates in Group F (controls) of 30% for NTHi and 24% for vaccine-type pneumococci, based on data from Vietnam (L Yoshida, personal communication).

For peer review only

15 Recruitment

Participants in Groups A-F are recruited from infants born in the study communes during the enrolment period. Potential participants are identified from commune health centre birth records and provided verbal and written information about the trial, in Vietnamese. Written informed consent is obtained when the infant is approximately two months old (Appendix 1), after which a study nurse/doctor examines the infant to ensure that all the eligibility criteria are met. Participants in Group G are recruited from children turning 18 months old in the study communes in parallel to the children in Groups A-F turning 18 months.

METHODS: ASSIGNMENT OF INTERVENTIONS

16 Allocation

16a Sequence generation

The allocation sequence for Groups A-F is produced using a computer-generated list of random numbers using a block randomisation scheme, stratified by district.

16b Allocation concealment mechanism

The group allocation is contained within a sealed envelope at the study clinic, with sequential ID numbers written on the outside of the envelope.

16c Implementation

The allocation sequence is generated at Menzies School of Health Research. A study doctor will enrol participants and assign them to a study group by selecting the next available envelope. The envelope is not opened until after completion of the informed consent and eligibility assessment processes.

17 Blinding (masking)

All laboratory staff are blinded to the study group allocation as the key outcome measures that address the study objectives are all laboratory based. Laboratory samples are labelled with the ID number, which does not identify the study group. Given the different timing of the vaccination schedules in the different groups, the study nurses, vaccine administrators and participants will not be blinded to the study group allocation.

For peer review only

METHODS: DATA COLLECTION, MANAGEMENT AND ANALYSIS

18 Data collection methods

Standardised carbon copy data collection forms are used and are completed by dedicated, trained study staff. The original is transported to the trial office for data entry, with the carbon copy filed at the clinic. Blood samples and NP swabs are collected by staff specifically trained in the collection of samples from infants, and the volume of blood collected and the swab quality are recorded.

Retention: Appointments are documented on a parent-held health record card and participants are given a small payment towards the transport costs of coming to the clinic for each study visit. Participants who miss a study visit will continue to be followed up for both sample collection and vaccine administration where possible, with attempts made to contact such participants until such time as they would have completed the study.

19 Data management

Data collection forms are double-entered by dedicated data entry staff into pre-coded EpiData version 3.1 files with built in range and consistency checks. Entered data are validated monthly and then uploaded to a central Microsoft Access database, stored on a secure server. Immunology results are double-entered in a Microsoft Excel spreadsheet. NP culture results are entered in a Microsoft Access database and qPCR and microarray results exported from SentiNET into a Microsoft Excel database. The data collection forms and laboratory results are linked at the time of analysis.

20 Statistical methods

20a Analysis of primary and secondary outcomes

For each of the two study questions, the primary objective is to compare a 2+1 schedule of 1) PCV10 and 2) PCV13, with a 3+1 schedule of PCV10. The primary outcome is the proportion of participants with serotype-specific antibody concentrations ≥0.35μg/mL, four weeks post-primary series (at 5 months of age). Data from Arms A and B are combined to form the three-dose post-primary series group. The primary analyses assess the non-inferiority of: 1) two doses of PCV10 at 2 and 4 months of age (Arm C) compared with three doses at 2, 3 and 4 months of age (Arms A+B); and 2) two doses of PCV13 at 2 and 4 months of age (Arm E) compared with three doses of PCV10 at 2, 3 and 4 months of age (Arms A+B). The proportion of children achieving protective levels of serotype-specific IgG

For peer review only - http://bmjopen.bmj.com/site/about/guidelines.xhtml

(≥0.35μg/ml) four weeks post-primary series is determined for each of the ten PCV10 serotypes. The non-inferiority margin is defined by a 10% difference in absolute risk. The serotype-specific risk differences (Arm A+B - Arm C) with 90% CIs are calculated using the Newcombe Score method, and the null hypothesis rejected if the upper bound of the CI is <10%. Overall non-inferiority is declared if at least seven of the ten individual null hypotheses are rejected at one-sided 5% level of significance. Secondary data analyses to address the primary objective include the ratio of GMCs post-primary series (Arm C / Arms A+B and Arm E / Arms A+B) with 95% CIs, and the booster response analysed by ANCOVA.

Analysis of key secondary objectives for study question 1:

- A single dose of PCV10 at 2 months of age (Arm D) will be assessed for non-inferiority to three doses at 2, 3 and 4 months of age (Arms A+B), as described for the primary objective
- The impact of a booster dose on pneumococcal and NTHi carriage will be assessed at 12 months of age. Overall pneumococcal, capsular pneumococcal, PCV10 type (with/without 6A and 19A) and NTHi carriage rates will be determined. Proportions will first be compared between the 3+1 group (Arm A) and the control group (Arm F), using Fisher's Exact test. Where significant differences are found, rates will then be compared between the 3+0 group (Arm B) and controls and between the 3+1 and 3+0 groups.

Analysis of key secondary objectives for study question 2:

- The immunogenicity of two doses of PCV10 or PCV13 will be compared in relation to the proportion of participants with serotype-specific antibody concentrations ≥0.35μg/mL (to the ten shared serotypes), four weeks post-primary series (at 5 months of age). A significant difference will be indicated by a 10% difference in absolute risk, comparing PCV10 (Arm C) with PCV13 (Arm E), and an overall difference will be declared if at least 7 of the 10 individual null hypotheses are rejected and the 7 differences are in the same direction.
- The immunogenicity of a single dose of PCV10 or PCV13 will be compared, as described for the immunogenicity of two doses.

20b Additional analyses

Descriptive analyses at the group level will be conducted on the OPA, ELISPOT and microarray data.

For peer review only

20c Populations of analysis

Analyses will be on an intention-to-treat population (ITT), with all participants analysed in the group they were randomised to. However, it is expected that withdrawn participants may not have blood and NP samples to contribute to the analysis after their withdrawal date. The primary non-inferiority analyses will be repeated on a per-protocol population and any differences between the ITT and per-protocol analyses reported. For each outcome, all available data will contribute to the analyses. To investigate whether data are missing completely at random, we will explore whether attrition varies across the study arms based on baseline covariates. If differential attrition is dependent on baseline variables, we will use a modelling approach to adjust for any such baseline factors and we will present the adjusted results along with the primary analysis.

Additional populations of analysis

- OPAs will be conducted on a subset of 100 participants per group. The first 100 participants per group with both post-primary series and post-booster blood samples available will contribute to the OPA analysis.
- B cell assays will be conducted on a subset of 50 participants per group for Arms A-E and 100 participants per group for Arms F and G. The last 50/100 participants enrolled per group will have blood samples collected for the B cell analysis.

Further details of the planned statistical methods can be found in the Statistical Analysis Plan.

For peer review only

METHODS: MONITORING

21 Data monitoring

Data monitoring committee: Safety oversight is under the direction of an independent Data Safety and Monitoring Board (DSMB), in accordance with a DSMB Charter kept in the trial office. The DSMB will meet approximately three times a year to review aggregate and individual participant data related to safety, data integrity and overall conduct of the trial, including a detailed review of all Serious Adverse Events (SAEs). Interim analyses and stopping guidelines: No interim analyses are planned. Stopping guidelines are based on safety. An extraordinary meeting of the DSMB will be called in the event that serious safety issues emerge, to provide recommendations regarding termination of the trial. A final decision to terminate rests with the Principal Investigators and the Sponsor.

22 Harms

Data on SAEs will be collected throughout the study, with parents asked about hospitalisations and significant signs and symptoms at each study visit and through a regular review of hospital records. Details of any SAEs will be recorded on the standard reporting form from the Vietnam Ministry of Health and reported to the Principal Investigators and the Ethics Committees. Participants will be kept under observation for 30 minutes following vaccine administration to monitor for any adverse reactions, and information on reactogenicity in the 72 hours following vaccine administration will be recorded on parent held diary cards.

23 Auditing

External site monitoring will be provided by FHI360, to independently assess protocol and GCP compliance. Monitoring visits will occur at study initiation, close-out and approximately twice a year in each study clinic. 100% of Informed Consent Forms and SAEs and a random selection of approximately 20% of participant folders will be monitored, along with the Trial Regulatory File and laboratory records.

For peer review only

ETHICS AND DISSEMINATION

24 Research ethics approval

The protocol, the Plain Language Statement (PLS) and the Informed Consent Form (ICF) have approval from the Institutional Review Board at the Pasteur Institute of Ho Chi Minh City, the Vietnam Ministry of Health Ethical Review Committee and the Human Research Ethics Committee of the Northern Territory Department of Health and the Menzies School of Health Research. Both Ethics Committees receive annual reports on the trial progress, for continuing approval of the trial.

25 Protocol amendments

Any modifications to the protocol that may impact on the conduct of the study will be documented in a formal protocol amendment and approved by both Ethics Committees prior to implementation of the changes. The modified protocol will be given a new version number and date. The Ethics Committees will also be notified of any minor corrections/clarifications or administrative changes to the protocol, which will be documented in a protocol amendment letter.

26 Consent

26a Obtaining consent: The consent process is undertaken by specifically trained study staff. The study staff will go through the PLS and ICF, translated into Vietnamese, in detail with the potential participant's parent/legal guardian. The study staff will then discuss the trial further and answer any questions that may arise. Written informed consent is required prior to enrolment of the infant into the study. Consent is obtained from the parent/legal guardian as the participants are too young to provide consent themselves. A copy of the PLS and ICF will be given to the parent/legal guardian for their records.

26b Ancillary studies: Specific consent for the indefinite storage of blood and NP samples for future research related to the trial will be obtained from the parent/legal guardian and recorded on the ICF. Any future research will undergo ethical review. Any samples for which indefinite storage is not consented to will be destroyed at the close of the trial.

For peer review only

27 Confidentiality

All study-related information will be stored securely and held in strict confidence. All documents kept at the study clinics, including the ICFs and participant folders, are stored in locked cabinets. All documents kept centrally are stored in the trial office, which is kept locked. Electronic data is stored in the trial office and on a secure password protected server. The electronic data and laboratory samples are coded by a unique participant number and do not contain the participant name. Access to participants' information will be granted to FHI360 for monitoring purposes, and to the Ethics Committees or DSMB if required.

28 Declaration of interests

All authors receive salary support from grants from the National Health and Medical Research Council of Australia and/or the Bill and Melinda Gates Foundation. Non-financial support (in the form of PCV10 vaccine doses) and funding for opsonphagocytic assays are provided by GSK Biologicals SA.

Prof. Mulholland is a member of the DSMB for a current Novavax trial, for which he receives consulting fees. He has received travel costs from the GSK group of companies for one international conference, and an honorarium from Merck for one advisory group meeting. He does not have any paid consultancies with or receive any research funds from pharmaceutical companies.

Member's of Dr Satzke's team have received awards that were funded (but not assessed) by Pfizer.

None of the authors have any other competing interests to declare.

For peer review only

29 Access to data

The final trial dataset will be under the custody of the trial sponsor, MCRI. The trial manager will have access to the full anonymised final dataset.

30 Ancillary and post-trial care

Participants are advised to come to the study clinic for ancillary care, or to Children's Hospital Number 2 in Ho Chi Minh City, where they will not be charged for treatment and services. All participants are covered by clinical trials insurance for trial related harms.

For peer review only - http://bmjopen.bmj.com/site/about/guidelines.xhtml

31 Dissemination policy

31a Plans

Participants will be informed of the overall study results by post, with a postal address collected at the final study visit. Following completion of the trial, the results will be submitted for publication in peer-reviewed journals, and presented at relevant international conferences. Agreements between MCRI and each of the Pasteur Institute of Ho Chi Minh City and GSK Biologicals SA provide that a party must obtain the prior approval of the other parties in advance of submitting a manuscript for publication, and that such approval will not be unreasonably withheld.

31b Authorship

A publication subcommittee will consider all proposed publications, with the final decision on content and authorship resting with the Principal Investigator. The role of each author will be published. Group authors may be used where appropriate. There are no plans for the use of professional writers.

31c Reproducible research

There are no plans to grant public access to the full protocol, participant-level dataset or statistical code.

For peer review only

References

1. Myint TT, Madhava H, Balmer P, et al. The impact of 7-valent pneumococcal conjugate vaccine on invasive pneumococcal disease: a literature review. *Advances in therapy* 2013;30:127-51. doi:10.1007/s12325-013-0007-6.
2. Conklin L, Loo JD, Kirk J, et al. Systematic review of the effect of pneumococcal conjugate vaccine dosing schedules on vaccine-type invasive pneumococcal disease among young children. *Pediatr Infect Dis J* 2014;33 Suppl 2:S109-18. doi:10.1097/INF.0000000000000078.
3. Loo JD, Conklin L, Fleming-Dutra KE, et al. Systematic review of the effect of pneumococcal conjugate vaccine dosing schedules on prevention of pneumonia. *Pediatr Infect Dis J* 2014;33 Suppl 2:S140-51. doi:10.1097/INF.0000000000000082.
4. Kalin M. Pneumococcal serotypes and their clinical relevance. *Thorax* 1998;53:159-62.
5. Hausdorff WP, Feikin DR, Klugman KP. Epidemiological differences among pneumococcal serotypes. *The Lancet. Infectious diseases* 2005;5:83-93. doi:10.1016/S1473-3099(05)01280-6.
6. Hausdorff WP, Bryant J, Paradiso PR, et al. Which pneumococcal serogroups cause the most invasive disease: implications for conjugate vaccine formulation and use, part I. *Clin Infect Dis* 2000;30:100-21. doi:10.1086/313608.
7. Hausdorff WP, Bryant J, Kloek C, et al. The contribution of specific pneumococcal serogroups to different disease manifestations: implications for conjugate vaccine formulation and use, part II. *Clin Infect Dis* 2000;30:122-40. doi:10.1086/313609.
8. Weinberger DM, Malley R, Lipsitch M. Serotype replacement in disease after pneumococcal vaccination. *Lancet* 2011;378:1962-73. doi:10.1016/S0140-6736(10)62225-8.
9. Vesikari T, Wysocki J, Chevallier B, et al. Immunogenicity of the 10-valent pneumococcal non-typeable Haemophilus influenzae protein D conjugate vaccine (PHiD-CV) compared to the licensed 7vCRM vaccine. *Pediatr Infect Dis J* 2009;28:S66-76. doi:10.1097/INF.0b013e318199f8ef.
10. Yeh SH, Gurtman A, Hurley DC, et al. Immunogenicity and safety of 13-valent pneumococcal conjugate vaccine in infants and toddlers. *Pediatrics* 2010;126:e493-505. doi:10.1542/peds.2009-3027.
11. Kieninger DM, Kueper K, Steul K, et al. Safety, tolerability, and immunologic noninferiority of a 13-valent pneumococcal conjugate vaccine compared to a 7-valent pneumococcal conjugate vaccine given with routine pediatric vaccinations in Germany. *Vaccine* 2010;28:4192-203. doi:10.1016/j.vaccine.2010.04.008.
12. Whitney CG, Pilishvili T, Farley MM, et al. Effectiveness of seven-valent pneumococcal conjugate vaccine against invasive pneumococcal disease: a matched case-control study. *Lancet* 2006;368:1495-502. doi:10.1016/S0140-6736(06)69637-2.
13. Mahon BE, Hsu K, Karumuri S, et al. Effectiveness of abbreviated and delayed 7-valent pneumococcal conjugate vaccine dosing regimens. *Vaccine* 2006;24:2514-20. doi:10.1016/j.vaccine.2005.12.025.
14. Goldblatt D, Southern J, Ashton L, et al. Immunogenicity and boosting after a reduced number of doses of a pneumococcal conjugate vaccine in infants and toddlers. *Pediatr Infect Dis J* 2006;25:312-9. doi:10.1097/01.inf.0000207483.60267.e7.

For peer review only - http://bmjopen.bmj.com/site/about/guidelines.xhtml

15. Russell FM, Balloch A, Tang ML, et al. Immunogenicity following one, two, or three doses of the 7-valent pneumococcal conjugate vaccine. *Vaccine* 2009;27:5685-91. doi:10.1016/j.vaccine.2009.06.098.
16. Silfverdal SA, Hogh B, Bergsaker MR, et al. Immunogenicity of a 2-dose priming and booster vaccination with the 10-valent pneumococcal nontypeable Haemophilus influenzae protein D conjugate vaccine. *Pediatr Infect Dis J* 2009;28:e276-82. doi:10.1097/INF.0b013e3181b48ca3.
17. Gutierrez Brito M, Thompson A, Girgenti D, et al. Immunogenicity and safety of 13-valent pneumococcal conjugate vaccine in Mexico. *Revista panamericana de salud publica = Pan American journal of public health* 2013;33:414-21.
18. Rodgers GL, Esposito S, Principi N, et al. Immune response to 13-valent pneumococcal conjugate vaccine with a reduced dosing schedule. *Vaccine* 2013;31:4765-74. doi:10.1016/j.vaccine.2013.08.009.
19. Russell FM, Licciardi PV, Balloch A, et al. Safety and immunogenicity of the 23-valent pneumococcal polysaccharide vaccine at 12 months of age, following one, two, or three doses of the 7-valent pneumococcal conjugate vaccine in infancy. *Vaccine* 2010;28:3086-94. doi:10.1016/j.vaccine.2010.02.065.
20. Huebner RE, Mbelle N, Forrest B, et al. Immunogenicity after one, two or three doses and impact on the antibody response to coadministered antigens of a nonavalent pneumococcal conjugate vaccine in infants of Soweto, South Africa. *Pediatr Infect Dis J* 2002;21:1004-7. doi:10.1097/01.inf.0000036012.37665.fe.
21. Barzilay EJ, O'Brien KL, Kwok YS, et al. Could a single dose of pneumococcal conjugate vaccine in children be effective? Modeling the optimal age of vaccination. *Vaccine* 2006;24:904-13. doi:10.1016/j.vaccine.2005.08.092.
22. O'Brien KL, Dagan R. The potential indirect effect of conjugate pneumococcal vaccines. *Vaccine* 2003;21:1815-25. doi:S0264410X02008071 [pii].
23. Kayhty H, Auranen K, Nohynek H, et al. Nasopharyngeal colonization: a target for pneumococcal vaccination. *Expert Rev Vaccines* 2006;5:651-67. doi:10.1586/14760584.5.5.651.
24. Moore MR, Hyde TB, Hennessy TW, et al. Impact of a conjugate vaccine on community-wide carriage of nonsusceptible Streptococcus pneumoniae in Alaska. *J Infect Dis* 2004;190:2031-8. doi:10.1086/425422.
25. Huang SS, Platt R, Rifas-Shiman SL, et al. Post-PCV7 changes in colonizing pneumococcal serotypes in 16 Massachusetts communities, 2001 and 2004. *Pediatrics* 2005;116:e408-13. doi:10.1542/peds.2004-2338.
26. Dunais B, Bruno P, Carsenti-Dellamonica H, et al. Trends in nasopharyngeal carriage of Streptococcus pneumoniae among children attending daycare centers in southeastern France from 1999 to 2006. *Pediatr Infect Dis J* 2008;27:1033-5. doi:10.1097/INF.0b013e31817bb8cf.
27. van Gils EJ, Veenhoven RH, Hak E, et al. Effect of reduced-dose schedules with 7-valent pneumococcal conjugate vaccine on nasopharyngeal pneumococcal carriage in children: a randomized controlled trial. *JAMA* 2009;302:159-67. doi:10.1001/jama.2009.975.
28. Clarke C, Bakaletz LO, Ruiz-Guinazu J, et al. Impact of protein D-containing pneumococcal conjugate vaccines on non-typeable Haemophilus influenzae acute otitis media and carriage. *Expert Rev Vaccines* 2017;16:1-14. doi:10.1080/14760584.2017.1333905.
29. Balloch A, Licciardi PV, Leach A, et al. Results from an inter-laboratory comparison of pneumococcal serotype-specific IgG measurement and critical parameters that affect assay performance. *Vaccine* 2010;28:1333-40. doi:10.1016/j.vaccine.2009.11.011.
30. WHO Expert Committee on Biological Standardization. WHO Technical Report Series 927. Geneva: World Health Organisation, 2005.

For peer review only

31. Feavers I, Knezevic I, Powell M, et al. Challenges in the evaluation and licensing of new pneumococcal vaccines, 7-8 July 2008, Ottawa, Canada. *Vaccine* 2009;27:3681-8. doi:10.1016/j.vaccine.2009.03.087.
32. World Health Organization. WHO/Health Canada Consulation on serological criteria for evaluation and licensing of new Pneumococcal vaccines, Ottawa, Canada, 7-8 July 2008. Secondary World Health Organization. WHO/Health Canada Consulation on serological criteria for evaluation and licensing of new Pneumococcal vaccines, Ottawa, Canada, 7-8 July 2008. http://www.who.int/biologicals/publications/meetings/areas/vaccines/pneumococcal/Pneumo Meeting Report FINAL IK 24 Dec 08.pdf?ua=1.
33. World Health Organization, Expert Committee on Biological Standardization. Recommendations to assure the quality, safety and efficacy of pneumococcal conjugate vaccines. Geneva, 19-23 October 2009. . Secondary World Health Organization, Expert Committee on Biological Standardization. Recommendations to assure the quality, safety and efficacy of pneumococcal conjugate vaccines. Geneva, 19-23 October 2009. . http://www.who.int/biologicals/areas/vaccines/pneumo/Pneumo_final_23APRIL_2010.pdf?ua=1.
34. Licciardi PV, Toh ZQ, Clutterbuck EA, et al. No long-term evidence of hyporesponsiveness after use of pneumococcal conjugate vaccine in children previously immunized with pneumococcal polysaccharide vaccine. *The Journal of allergy and clinical immunology* 2016;137:1772-79 e11. doi:10.1016/j.jaci.2015.12.1303.
35. Richter SS, Heilmann KP, Dohrn CL, et al. Accuracy of phenotypic methods for identification of Streptococcus pneumoniae isolates included in surveillance programs. *J Clin Microbiol* 2008;46:2184-8. doi:10.1128/JCM.00461-08.
36. Satzke C, Turner P, Virolainen-Julkunen A, et al. Standard method for detecting upper respiratory carriage of Streptococcus pneumoniae: updated recommendations from the World Health Organization Pneumococcal Carriage Working Group. *Vaccine* 2013;32:165-79. doi:10.1016/j.vaccine.2013.08.062.
37. Turner P, Hinds J, Turner C, et al. Improved detection of nasopharyngeal cocolonization by multiple pneumococcal serotypes by use of latex agglutination or molecular serotyping by microarray. *J Clin Microbiol* 2011;49:1784-9. doi:10.1128/JCM.00157-11.
38. Smith-Vaughan H, Byun R, Nadkarni M, et al. Measuring nasal bacterial load and its association with otitis media. *BMC Ear Nose Throat Disord* 2006;6:10. doi:10.1186/1472-6815-6-10.
39. Price EP, Harris TM, Spargo J, et al. Simultaneous identification of Haemophilus influenzae and Haemophilus haemolyticus using real-time PCR. *Future microbiology* 2017;12:585-93. doi:10.2217/fmb-2016-0215.
40. Feiveson A. Power by simulation. *The Stata Journal* 2002;2:107-24.
41. Farrington CP, Manning G. Test statistics and sample size formulae for comparative binomial trials with null hypothesis of non-zero risk difference or non-unity relative risk. *Stat Med* 1990;9:1447-54.
42. Dinleyici EC, Yargic ZA. Current knowledge regarding the investigational 13-valent pneumococcal conjugate vaccine. *Expert Rev Vaccines* 2009;8:977-86. doi:10.1586/erv.09.68.
43. Immunogenicity, safety, and tolerability of 13-valent pneumococcal conjugate vaccine given to infants with routine pediatric vaccinations in Mexico: Interim Report. Abstract: 7th International Symposium on Pneumococci and Pneumococcal Diseases; 2010; Tel Aviv, Israel.
44. Prymula R, Schuerman L. 10-valent pneumococcal nontypeable Haemophilus influenzae PD conjugate vaccine: Synflorix. *Expert Rev Vaccines* 2009;8:1479-500. doi:10.1586/erv.09.113.

For peer review only - http://bmjopen.bmj.com/site/about/guidelines.xhtml

SECTION 5: Appendices

32 Informed consent materials

Appendix 1 - Plain Language Statement and Informed Consent Form. These materials were translated into Vietnamese, and back-translated into English, by FHI360.

33 Biological specimens

Appendix 2 - Biological Specimens

For peer review only

APPENDIX 1

Plain Language Statements and Informed Consent Form

These materials were translated into Vietnamese, and back-translated into English, by FHI360. This trial uses two Plain Language Statements, one for participants enrolled at 2 months of age and randomised into Arms A-F, and one for participants enrolled at 18 months of age into Arm G. The same Informed Consent Form is used for participants in all Arms.

For peer review only

menzies
school of health research

PO Box 41096, Casuarina NT 0811, Australia
John Mathews Building (Bldg 58),
Royal Darwin Hospital Campus, Rocklands Dve, Casuarina NT 0810
Ph: 08 8922 8196 | Fax: 08 8927 5187 | Web: www.menzies.edu.au
ABN: 70 413 542 847

discovery for a healthy tomorrow

INFORMATION SHEET: Vietnam Pneumococcal Vaccine Study

This is for you to keep.

Principal Investigators:
Assoc. Prof. Tran Ngoc Huu
Prof. Edward Kim Mulholland

Research Partners:
Pasteur Institute, Ho Chi Minh City
Menzies School of Health Research
Murdoch Childrens Research Institute

Introduction

Health research helps us to understand diseases and find ways to prevent them. Vaccines (like the routine baby injections) are an important way to prevent diseases. Pneumonia is a common problem in Vietnam and throughout the developing world. In the developing world it is the leading cause of death amongst under 5 year olds. A number of germs cause pneumonia but the most common germ is a bacteria called pneumococcus. Pneumococcus can also cause ear infections as well as other, more severe diseases like meningitis (infection around the brain). This germ normally lives in the nose of humans and is spread from person to person by touching or sneezing. There are more than 90 types of this germ but only some types cause serious infections in young children.

Why are we doing the study?

There are vaccines available to protect against infection with pneumococcus. These are called pneumococcal vaccines. Many countries around the world give all their babies a pneumococcal vaccine that protects against 7 types of the pneumococcal disease (7v-PCV). There are two new vaccines which have been developed. Both new vaccines give more protection against pneumococcal disease than the 7v-PCV. Both vaccines have completed all their tests and are licensed and being used by many countries in Europe and the United States. The clinical trials have shown that these vaccines are safe; therefore there is little danger to any child participating in this study. The vaccines are likely to provide some protection from ear infections and pneumonia. Unfortunately the costs of these vaccines are very high, so not all countries in the world can afford them. We are doing this study to find the best ways to protect babies from this germ and also to make it cheaper for countries, like Vietnam, to afford to buy the vaccine.

Benefits of the study

By joining the study your baby can be protected from the commonest pneumococcal germs. Both these vaccines are very expensive and are not presently available to other babies in Vietnam. They have been especially made for use in babies and young children and will protect the babies from the common diseases caused by the pneumococcus. We hope to find a schedule that works and which countries like Vietnam can afford. In addition children will receive 4 doses of *Infanrix-Hexa*: 3 doses during early infancy and a booster dose at either 18 or 19 months of age.

For peer review only - http://bmjopen.bmj.com/site/about/guidelines.xhtml

What does the study involve?

The study will include 1400 babies and we will be looking at 7 different vaccine schedules in this study. 1200 babies will be enrolled at 2 months old and will be randomly allocated to 1 of 6 groups. An additional 200 babies will be enrolled at 18 months old to act as controls.

Consent: A study doctor or nurse will discuss the study with each child's parent or legal guardian. They will explain what is involved and ask some questions about the baby's health. If you agree to join the study she will ask you to sign a consent form which says that you agree for your baby to join. If you consent to taking part in the study, she will perform a health check of your baby to make sure your baby is healthy to take part.

Vaccinations & health checks: If you agree to your baby to take part in the study you will need to come to the clinic between 9 and 11 times over a period of 22 months. The study nurse will remind you when you need to come. Like rolling a dice your baby will be allocated to 1 of 6 groups. Your baby will get between one and four doses of one of the two types of Pneumococcal vaccine, either the Prevnar-13 (13v-PCV which covers 13 types of the pneumococcal germ) or the 10v-Synflorix vaccine (which covers 10 types of the pneumococcal germ and may be better at protecting against pneumonia). Depending on which group your baby is randomly placed in will depend on when, how many doses and what type of Pneumococcal vaccine your baby will receive. Your baby will also get an infant vaccine (Infanrix-hexa 6-1) that covers all the diseases (diphtheria, tetanus, pertussis, hepatitis B, polio virus and *Haemophilus influenzae* type B) that are covered by the standard vaccines used in VIetnam. Vaccines will be given by staff from Pasteur Institute Ho Chi Minh City. Your baby will also have regular health checks during the study.

Questionnaire: At the start of the study you will be asked some general questions about your family and your baby's health. These are simply to help us understand how the vaccines work best. The results will be kept confidential (see below).

Blood tests: Up to four blood tests will be taken during the study, by staff from Children's Hospital Number 2. The blood tests are to check the response to the vaccines. If you would prefer, we can put local anesthetic cream on your baby's skin before taking the blood test so that it doesn't hurt as much. The amount of blood taken will vary depending on the age of the child: 2.0mls at 2 months of age; 3.5mls from 3 to 10 and 19 months of age; and 3.5mls or 7.5mls at 18 and 24 months of age.

Nose swabs: Six nose swabs will be taken during the study, at 2, 6, 9, 12, 18 and 24 months of age. The nose swabs are to see if the vaccine will help stop the spread of the pneumococcus from child to child. This will involve putting a cotton wool swab (like a cotton bud) into the baby's nose for a couple of seconds. This may make the baby sneeze and possibly cry briefly – it tickles quite a lot, but doesn't really hurt.

Summary of changes: Additional procedures and vaccines

Groups A-E	*18 months*	*Measles and Rubella given*
	19 months	*Infanrix Hexa given*
	24 months	*Nose swab taken*
Group F	*18 months*	*Infanrix Hexa given*
	19 months	*Measles and Rubella given*<br>*Blood taken*
	24 months	*Nose swab taken*<br>*Blood taken*<br>*Synflorix given*

For peer review only - http://bmjopen.bmj.com/site/about/guidelines.xhtml

Hospital record review: If your baby becomes unwell during the study, the staff may need to look at your child's medical records.

Are there any risks?

The vaccines we are using are licensed many countries. As with all vaccines there is likely to be some pain felt, and there is a small risk of soreness and redness where the vaccine was given. Babies in the study will get up to 4 extra injections than they would routinely get. We will check the babies to make sure they don't have any unexpected reactions. We also have a study doctor who will be keeping a record of any serious illnesses that are unlikely to occur during the study.

Confidentiality

All information collected in this study will remain confidential and will be used for research purposes only. All information will be kept secure. Your baby will be given an identification number at the start of the study. Any information collected will use this number and will not include your baby's name. The samples we collect will be sent to overseas laboratories to have further tests. These laboratories will not be given your child's name. We will ask your permission if it is alright for your baby's blood and nose swab samples to be stored indefinitely for other similar tests in the future. This would help us to perform any new pneumococcal test that may be developed in the future. The results of the study will be published in scientific journals and presented at conferences. There will never be details published that would identify your baby.

Voluntary Participation and Withdrawal from the Study

Your baby does not have to take part in the study. Your baby will get the best treatment available and the full attention of the health staff even if they do not participate. You are free to withdraw your baby from the study at any point. This will not affect any of your baby's further health care treatment and there will be no harmful consequences for your baby. If your baby has not had all their pneumococcal vaccines they may not be fully protected against the pneumococcal germs which most commonly affect infants. However, they will still gain some protection from the doses of vaccine received.

Compensation

We will pay 200,000VND towards the transport cost for coming to the clinic for each study visit. If your baby becomes ill or injured as a result of taking part in this clinical study, medical treatment will be provided.

Ethical Approval

This study has been approved by the People's Committee of Ho Chi Minh City. This study has also been approved by the Vietnam Ministry of Health Ethics Committee and the Menzies School of Health Research Ethics Committee, Australia. The ethics committees make sure that the study is being done in the best and safest way. If you have any concerns or complaints regarding the conduct of the research project you are invited to contact:

Vietnam Ministry of Health Ethics Committee
Phone: 04 62732156

OR

Human Research Ethics Committee of the NT Department of Health and Menzies School of Health Research
PO Box 41096, Casuarina, NT 0811, Australia
Phone: 61 8 8922 7922
Email: ethics@menzies.edu.au

For peer review only

For peer review only - http://bmjopen.bmj.com/site/about/guidelines.xhtml

How is the study funded?

The funding to perform the study is from the National Health and Medical Research Council, Australia and the Bill & Melinda Gates Foundation.

Your Right to Ask Questions

Please feel free to contact us if you have any questions or concerns.

If you have any questions regarding the study activities, please phone:

If you have any questions regarding adverse events, please phone:

Commune Health Centre Number:

For peer review only

For peer review only - http://bmjopen.bmj.com/site/about/guidelines.xhtml

menzies school of health research
PO Box 41096, Casuarina NT 0811, Australia
John Mathews Building (Bldg 58),
Royal Darwin Hospital Campus, Rocklands Dve, Casuarina NT 0810
Ph: 08 8922 8196 | Fax: 08 8927 5187 | Web: www.menzies.edu.au
ABN: 70 413 542 847

discovery for a healthy tomorrow

INFORMATION SHEET: Vietnam Pneumococcal Vaccine Study (Control group)

This is for you to keep.

Principal Investigators:
Assoc. Prof. Tran Ngoc Huu
Prof. Edward Kim Mulholland

Research Partners:
Pasteur Institute, Ho Chi Minh City
Menzies School of Health Research
Murdoch Children's Research Institute

Introduction

Health research helps us to understand diseases and find ways to prevent them. Vaccines (like the routine baby injections) are an important way to prevent diseases. Pneumonia is a common problem in Vietnam and throughout the developing world. In the developing world it is the leading cause of death amongst under 5 year olds. A number of germs cause pneumonia but the most common germ is a bacteria called pneumococcus. Pneumococcus can also cause ear infections as well as other, more severe diseases like meningitis (infection around the brain). This germ normally lives in the nose of humans and is spread from person to person by touching or sneezing. There are more than 90 types of this germ but only some types cause serious infections in young children.

Why are we doing the study?

There are vaccines available to protect against infection with pneumococcus. These are called pneumococcal vaccines. Many countries around the world give all their babies a pneumococcal vaccine that protects against 7 types of the pneumococcal disease (7v-PCV). There are two new vaccines which have been developed. Both new vaccines give more protection against pneumococcal disease than the 7v-PCV.Both vaccines have completed all their tests and are licensed and being used by many countries in Europe and the United States. The clinical trials have shown that these vaccines are safe; therefore there is little danger to any child participating in this study. The vaccines are likely to provide some protection from ear infections and pneumonia. Unfortunately the costs of these vaccines are very high, so not all countries in the world can afford them. We are doing this study to find the best ways to protect babies from this germ and also to make it cheaper for countries, like Vietnam, to afford to buy the vaccine.

Benefits of the study

By joining the study your baby can be protected from the commonest pneumococcal germs. Both these vaccines are very expensive and are not presently available to other babies in Vietnam. They have been especially made for use in babies and young children and will protect the babies from the common diseases caused by the pneumococcus. We hope to find a schedule that works and which countries like Vietnam can afford. In addition your baby will receive a dose of Infanrix-hexa at 18 months of age.

For peer review only

For peer review only - http://bmjopen.bmj.com/site/about/guidelines.xhtml

What does the study involve?

The study will include 200 babies to act as comparisons to participants in an existing study of six different vaccine schedules.

Consent: A study doctor or nurse will discuss the study with each child's parent or legal guardian. They will explain what is involved and ask some questions about the baby's health. If you agree to join the study she will ask you to sign a consent form which says that you agree for your baby to join. If you consent to taking part in the study, she will perform a health check of your baby to make sure your baby is healthy to take part.

Vaccinations & health checks: If you agree to your baby to take part in the study you will need to come to the clinic 3 times over a period of 6 months. The study nurse will remind you when you need to come. Your baby will get a single dose of (Infanrix-hexa 6-1) that covers six diseases (diphtheria, tetanus, pertussis, hepatitis B, polio virus and *Haemophilus influenzae* type B) at 18 months of age, a single dose of Measles and Rubella (MR) at 19 months of age and a single dose of Pneumococcal vaccine (10v-Synflorix vaccine, which covers 10 types of the pneumococcal germ) at 24 months of age. Vaccines will be given by staff from Pasteur Institute Ho Chi Minh City. Your baby will also have a doctor's health check at each study visit.

Questionnaire: At the start of the study you will be asked some general questions about your family and your baby's health. These are simply to help us understand how the vaccines work best. The results will be kept confidential (see below).

Blood tests: Three blood tests will be taken over the six months, by staff from Children's Hospital Number 2. The blood tests are to check the response to the vaccines. If you would prefer, we can put local anesthetic cream on your baby's skin before taking the blood test so that it doesn't hurt as much. The amount of blood taken will be 3.5 or 7.5mls at 18 and 24 months of age; and 3.5mls at 19 months of age.

Nose swabs: Two nose swabs will be taken during the study, at 18 and 24 months of age. The nose swabs are to see if the vaccine will help stop the spread of the pneumococcus from child to child. This will involve putting a cotton wool swab (like a cotton bud) into the baby's nose for a couple of seconds. This may make the baby sneeze and possibly cry briefly – it tickles quite a lot, but doesn't really hurt.

Hospital record review: If your baby becomes unwell during the study, the staff may need to look at your child's medical records.

Are there any risks?

The vaccines we are using are licensed many countries. As with all vaccines there is likely to be some pain felt, and there is a small risk of soreness and redness where the vaccine was given. We will check the babies to make sure they don't have any unexpected reactions. We also have a study doctor who will be keeping a record of any serious illnesses that are unlikely to occur during the study.

Confidentiality

All information collected in this study will remain confidential and will be used for research purposes only. All information will be kept secure. Your baby will be given an identification number at the start of the study. Any information collected will use this number and will not include your baby's name. The samples we collect will be sent to overseas laboratories to have further tests. These laboratories will not be given your child's name. We will ask your permission if it is alright for your baby's blood and

For peer review only - http://bmjopen.bmj.com/site/about/guidelines.xhtml

nose swab samples to be stored indefinitely for other similar tests in the future. This would help us to perform any new pneumococcal test that may be developed in the future. The results of the study will be published in scientific journals and presented at conferences. There will never be details published that would identify your baby.

Voluntary Participation and Withdrawal from the Study

Your baby does not have to take part in the study. Your baby will get the best treatment available and the full attention of the health staff even if they do not participate. You are free to withdraw your baby from the study at any point. This will not affect any of your baby's further health care treatment and there will be no harmful consequences for your baby. If your baby has not had all their pneumococcal vaccines they may not be fully protected against the pneumococcal germs which most commonly affect infants. However, they will still gain some protection from the doses of vaccine received.

Compensation

We will pay 200,000VND towards the transport cost for coming to the clinic for each study visit. If your baby becomes ill or injured as a result of taking part in this clinical study, medical treatment will be provided.

Ethical Approval

This study has been approved by the People's Committee of Ho Chi Minh City. This study has also been approved by the Vietnam Ministry of Health Ethics Committee and the Menzies School of Health Research Ethics Committee, Australia. The ethics committees make sure that the study is being done in the best and safest way. If you have any concerns or complaints regarding the conduct of the research project you are invited to contact:

Vietnam Ministry of Health Ethics Committee<br>Phone: 04 62732156	OR	Human Research Ethics Committee of the NT Department of Health and Menzies School of Health Research<br>PO Box 41096, Casuarina, NT 0811, Australia<br>Phone: 61 8 8922 7922<br>Email: ethics@menzies.edu.au

How is the study funded?

The funding to perform the study is from the National Health and Medical Research Council, Australia and the Bill & Melinda Gates Foundation.

Your Right to Ask Questions

Please feel free to contact us if you have any questions or concerns.

If you have any questions regarding the study activities, please phone:

If you have any questions regarding adverse events, please phone:

Commune Health Centre Number:

For peer review only - http://bmjopen.bmj.com/site/about/guidelines.xhtml

menzies
school of health research

PO Box 41096, Casuarina NT 0811, Australia
John Mathews Building (Bldg 58),
Royal Darwin Hospital Campus, Rocklands Dve, Casuarina NT 0810
Ph: 08 8922 8196 | Fax: 08 8927 5187 | Web: www.menzies.edu.au
ABN: 70 413 542 847

discovery for a healthy tomorrow

CONSENT FORM

This means you can say NO

Screening Number: |___|___|___|___|

Participant ID: |___|___|___|___|

Date: __ __ /__ __ /__ __
dd / mm / yy

Principal Investigators:

Assoc. Prof. Tran Ngoc Huu
Prof. Edward Kim Mulholland

Research Partners:

Pasteur Institute, Ho Chi Minh City
Menzies School of Health Research

This form is to record if you agree for your infant to take part in the **"Evaluation of Different Infant Vaccination Schedules Incorporating Pneumococcal Vaccination"**. You should only sign this form if you are happy that the information about the study has been clearly explained to you, you have received enough information about the study and you have had all your questions answered satisfactorily.

Please record the name of the person you have spoken to about the study:

____

By agreeing for your infant to take part in the study, you understand that:

- You are free to withdraw your child from the study at any time without having to give a reason;
- Your child will be vaccinated against all the diseases that are covered by the standard vaccines used in Vietnam, although these vaccines may be given at different times;
- If your child becomes sick, their hospital records will be reviewed by the study doctor or other designated study staff; and
- The samples taken in this study will be sent to overseas laboratories to test vaccine responses and carriage of bacteria

For peer review only

For peer review only - http://bmjopen.bmj.com/site/about/guidelines.xhtml

menzies
school of health research

PO Box 41096, Casuarina NT 0811, Australia
John Mathews Building (Bldg 58),
Royal Darwin Hospital Campus, Rocklands Dve, Casuarina NT 0810
Ph: 08 8922 8196 | Fax: 08 8927 5187 | Web: www.menzies.edu.au
ABN: 70 413 542 847

discovery for a healthy tomorrow

Screening Number: |___|___|___|___|

Participant ID: |___|___|___|___|

Date: __ __ /__ __ /__ __

Consent:

❑ YES, I agree for my infant to take part in this study.

❑ NO, I do not agree for my infant to take part in this study.

Use of samples:

❑ YES, you may indefinitely store my unused identified samples for future work in the same general area of research that has obtained ethics committee approval.

❑ NO, you may **NOT USE** my samples for future research. Destroy my unused samples at the close of the study.

Signed (parent/legal guardian): ______________________ Date: __ __ /__ __ /__ __
dd / mm / yy

Name of parent/legal guardian: ______________________ Time: __ __ :__ __
hh : mm

Relationship to infant: ______________________

Name of infant or baby of: ______________________

Infant Sex: male / female Infant DOB: __ __ /__ __ /__ __
dd / mm / yy

Signed (study nurse): ______________________ Date: __ __ /__ __ /__ __
dd / mm / yy

If illiterate: *A literate witness must sign (if possible, this person should be selected by the participant and should have no connection to the research team).*

I have witnessed the accurate reading of the consent form to the parent of the potential participant, and the individual has had the opportunity to ask questions. I confirm that the individual has given consent freely.

Signed (witness): ______________________ Date: __ __ /__ __ /__ __
dd / mm / yy

Name of witness: ______________________

For peer review only - http://bmjopen.bmj.com/site/about/guidelines.xhtml

APPENDIX 2

Biological Specimens

Specimens include NP swabs, bacterial isolates cultured from NP swabs, serum from whole blood, plasma from whole blood and peripheral blood mononuclear cells (PBMCs). Specimens are stored at the Pneumococcal Laboratory at MCRI or at the Pasteur Institute of Ho Chi Minh City at -80°C. No genetic or HIV testing will be performed on stored samples and they will not be used to establish a tissue bank. Consent for the long-term storage of samples and their use in potential future studies is recorded on the ICF.

Sample Collection

Blood samples are collected using a butterfly needle into gel vacutainer tubes or sodium heparin vacutainer tubes. The volume of blood collected at different ages is as follows: 2.0ml at 2 months of age; 3.5ml from 3-10 months and 19 months of age; and 3.5ml or 7.5ml at 18 months and 24 months of age, depending on the assays to be conducted. Blood samples collected into gel vacutainer tubes are kept chilled in a cooler box and transported to the Pasteur Institute laboratory the same day. On arrival at the laboratory the samples are centrifuged and the sera divided into up to three aliquots, stored in micro-tubes and frozen at -80°C prior to analysis. For blood samples where plasma cell and memory B cell responses are assessed, samples are collected into sodium heparin vacutainer tubes and transported to the Pasteur Institute laboratory at room temperature the same day. On arrival at the laboratory plasma and PBMCs are separated from each heparinized blood sample by density gradient centrifugation. Plasma are divided into up to four aliquots and stored at -80°C prior to analysis.

NP samples are collected and transported according to standard guidelines.[1] In brief, NP samples are collected using sterile swabs and placed immediately into 1000µL Skim Milk Tryptone Glucose Glycerol (STGG) transport medium. The samples are kept chilled in a cooler box and transported to the Pasteur Institute laboratory the same day. On arrival at the laboratory two aliquots are removed and the aliquots and original sample are frozen at -80°C prior to analysis.

Serotype-specific IgG

For peer review only

Serotype-specific anti-pneumococcal IgG levels to each of the 13 serotypes in 13v-PCV are measured using a modified 3rd generation standardized ELISA at the Pasteur Institute laboratory.[2] Briefly, microtiter wells are coated with 2.5-10 mg/mL pneumococcal polysaccharide, depending on the serotype. This is diluted in phosphate buffered saline by incubating at 22° C overnight. To neutralize unspecified cell wall polysaccharide antibodies, 1/100 diluted serum samples are incubated overnight with 10 mg/mL of cell wall polysaccharide and 30mg/mL of serotype 22F, before further dilutions. A reference serum (89-SF, Food and Drug Administration, Bethesda MD) is used and incubated overnight with 10 mg/mL of cell wall polysaccharide. Horse radish peroxidase conjugated anti-human IgG and the TMB Peroxidase Substrate system is used for detection. Results are expressed as µg/mL of serotype-specific IgG. Three control sera will be used on each plate to assess inter-assay variation.

Opsonophagocytic Assay (OPA)

OPAs are conducted at the Pneumococcal Laboratory at MCRI.[3] Serial dilutions of a heat-inactivated sera, in Hanks balanced salt solution with Mg^{++}, Ca^{++} and gelatine, are made in a 96-well sterile microtitre plate. Frozen stock of pneumococci are thawed, washed and diluted to 5×10^4 CFU/serotype/mL. Standard bacterial dilutions are added to all wells and the plate incubated at RT for 30 min. At 30 min, baby rabbit complement, thawed just prior to use, followed by HL-60 cells (2×10^7 cells/ml) is added to all test wells. A bacterial control (heat inactivated foetal calf serum in place of human sera and no complement) and complement control (no sera) are included on all plates. Plates are placed on a horizontal shaker and incubated for 45 min at 37°C in 5% CO_2. The reaction is stopped at 45 min by placing the plate on ice. A 10µL aliquot of this mixture is then spotted onto Todd-Hewitt broth–yeast extract (0.5%) agar plates. After application of an overlay THYE agar containing selective antibiotic (Optochin, Spectinomycin, Streptomycin or Trimethoprim) and 2,3,5-Triphenyltetrazolium chloride (TTC), the plates are incubated overnight at 37°C in 5% CO_2. After overnight incubation, plates are counted and the results expressed as opsonisation indices (OI) where the OI is defined as the interpolated dilution of serum that kills 50% of bacteria.

Memory B cells

Analysis of the memory B cell response is undertaken at the Pasteur Institute laboratory, by ELISPOT assay.[3] PBMCs are re-suspended in RPMI Foetal Calf

For peer review only

Serum (FCS) at a concentration of $2x10^6$ cells/mL and 100μL added to each well of the culture plate containing an antigen cocktail (Staphylococcus aureus Cowan strain – Pansorbin cells (SAC; 1:5000), 2.5μg/mL CpG and 83ng/mL pokeweed mitogen). Plates are incubated at 37°C with 5% CO_2 and 95% humidity for 5 days. At day 5, cells are harvested and washed and the cell pellet re-suspended in 1mL RPMI-FCS and counted by trypan blue. Cells are then made up to a final concentration of $2x10^6$ cells/mL for seeding onto antigen-coated ELISPOT plates. Multiscreen hydrophobic polyvinyldene difluoride (PVDF) membrane ELISPOT plates coated with anti-IgG (10μg/mL), tetanus toxoid (5μg/mL), diphtheria toxoid (10μg/mL) or pneumococcal polysaccharides conjugated to methylated human serum albumin at concentrations in the range 10-20μg/mL are sealed and incubated overnight at 4°C. ELISPOT plates are then washed and blocked with RPMI-FCS for 30 minutes at 37°C with 5% CO2 and 95% humidity. Cultured cells or *ex vivo* PBMCs are washed and seeded at 200 to $2x10^5$ cells/well of the antigen-coated ELISPOT plates in RPMI-FCS and incubated overnight at 37°C with 5% CO_2 and 95% humidity. Cells are then washed with PBS-T and bound IgG detected with an alkaline phosphatase-conjugated IgG for 4 hours at RT. ELISPOT plates are washed again before addition of an alkaline phosphatase substrate solution (nitroblue tetrazolium plus 5-bromo-4-chloro-3-indoylphosphate in dimethyl formamide). The reaction is stopped with two washes in distilled water. Cells are visualized and counted using an automated ELISPOT reader and software. The total frequency of IgG-secreting antibody-forming cells (AFCs) is used as the positive control and 1,000 IgG AFCs/10^6 cultured PBMCs is the lower cut-off for inclusion in the analysis. Up to $15x10^6$ cells/mL are used for the memory B cell assay at the Pasteur Institute and the remainder of the PBMCs are cryopreserved in liquid nitrogen in aliquots of 8-$10x10^6$ cells/mL for planned T cell assays.

S. pneumoniae identification and serotyping

Identification of *S. pneumoniae* is conducted in line with WHO guidelines.[1] In brief, 50μl swab is plated onto Columbia colistin-nalidixic acid blood agar plates, and identification is primarily based on colonial morphology (flat, with a dimple, 1-3mm in size), α-haemolysis and optochin sensitivity. One colony, plus any additional colonies if morphologically distinct, is sub-cultured onto horse blood agar with an optochin disc. Any colonies that are optochin resistant or intermediately resistant but otherwise appear to be *S. pneumoniae* are subject to *lytA* PCR,[1] following DNA preparation using the InstaGene matrix (BioRad). All presumptive pneumococci are serotyped, primarily by latex agglutination using reagents produced in-house using

For peer review only

antisera from the Statens SerumInstitut, as previously described.[4 5] In summary, pneumococcal culture is made to a 4-5 McFarland density standard and then 10μL of the suspension mixed with 10μL of latex reagent on clear glass slides and rotated for 1 minute. A positive test is indicated by aggregation of latex particles and clearing of the suspension. Isolates that do not react with antisera are subject to *lytA* PCR.

H. influenzae identification

Identification of *H. influenzae* is made from 50μl swab plated onto bacitracin-vancomycin-clindamycin-chocolate-agar. One presumptive *H. influenzae* colony, plus any additional colonies if morphologically distinct, is selected. Colonies are identified as grayish, semi-opaque, smooth, flat or convex, 1-3mm in size. Confirmation is initially demonstrated by X and V growth factor dependence. Capsular and NTHi strains are discriminated using the Phadebact® Haemophilus coagglutination test. All NTHi isolates are tested for beta-lactamase production using nitrocefin.[6] Following identification of presumptive NTHi, DNA is extracted using the InstaGene matrix (BioRad)[7] and tested by *siaT* and *hypD* PCR for discrimination between NTHi and *H. haemolyticus*.[8]

Quantification of *H. influenzae* and pneumococcus

DNA is extracted from 100μl of STGG medium using high-throughput systems (MagNA Pure LC, Roche) using the DNA Isolation Kit II (Bacteria, Fungi) (Roche) incorporating enzymatic digestion. Quantification of *H. influenzae* and pneumococci is then performed using real-time quantitative PCR (qPCR).[9] qPCR targeting the *hpd3* and/or *siaT* gene (*H. influenzae*) or *lytA* gene (pneumococcus) is conducted in 25μl reactions containing 2μl of template DNA on a Stratagene Mx3005 machine using Brilliant III Ultra-Fast qPCR Master Mix (Agilent Technologies) according to the manufacturer's instructions. The density of each bacterial species is assessed in comparison to a set of approximately five reference standards run with each assay to give the density of carriage.

Microarray serotyping

Samples that contain pneumococci are tested by DNA microarray as described previously with minor modifications.[4] Following a culture amplification step (on selective agar such as horse blood agar with 5 μg/ml gentamicin), DNA is extracted using the Qiacube HT platform (Qiagen). When only a single α-haemolytic colony grows, it is sub-cultured before DNA extraction for microarray. DNA is labelled and then hybridised to the Senti-SP microarray (formally BUGS microarray), scanned on

For peer review only

an Agilent scanner, and uploaded to Senti-Net (a cloud based software platform). Serotype-specific density is calculated by multiplying pneumococcal density (measured by *lytA* qPCR) by the relative abundance of each serotype (determined by microarray).

Appendix 2 - References

1. Satzke C, Turner P, Virolainen-Julkunen A, et al. Standard method for detecting upper respiratory carriage of Streptococcus pneumoniae: updated recommendations from the World Health Organization Pneumococcal Carriage Working Group. *Vaccine* 2013;32:165-79. doi:10.1016/j.vaccine.2013.08.062.
2. Balloch A, Licciardi PV, Leach A, et al. Results from an inter-laboratory comparison of pneumococcal serotype-specific IgG measurement and critical parameters that affect assay performance. *Vaccine* 2010;28:1333-40. doi:10.1016/j.vaccine.2009.11.011.
3. Licciardi PV, Toh ZQ, Clutterbuck EA, et al. No long-term evidence of hyporesponsiveness after use of pneumococcal conjugate vaccine in children previously immunized with pneumococcal polysaccharide vaccine. *The Journal of allergy and clinical immunology* 2016;137:1772-79 e11. doi:10.1016/j.jaci.2015.12.1303.
4. Satzke C, Dunne EM, Porter BD, et al. The PneuCarriage Project: A Multi-Centre Comparative Study to Identify the Best Serotyping Methods for Examining Pneumococcal Carriage in Vaccine Evaluation Studies. *PLoS medicine* 2015;12:e1001903; discussion e03. doi:10.1371/journal.pmed.1001903.
5. Porter BD, Ortika BD, Satzke C. Capsular Serotyping of Streptococcus pneumoniae by latex agglutination. *Journal of visualized experiments : JoVE* 2014:51747. doi:10.3791/51747.
6. Tenover FC, Huang MB, Rasheed JK, et al. Development of PCR assays to detect ampicillin resistance genes in cerebrospinal fluid samples containing Haemophilus influenzae. *J Clin Microbiol* 1994;32:2729-37.
7. Carter J, Hutton S, Sriprakash KS, et al. Culture of the causative organism of donovanosis (Calymmatobacterium granulomatis) in HEp-2 cells. *J Clin Microbiol* 1997;35:2915-7.
8. Price EP, Harris TM, Spargo J, et al. Simultaneous identification of Haemophilus influenzae and Haemophilus haemolyticus using real-time PCR. *Future microbiology* 2017;12:585-93. doi:10.2217/fmb-2016-0215.
9. Smith-Vaughan H, Byun R, Nadkarni M, et al. Measuring nasal bacterial load and its association with otitis media. *BMC Ear Nose Throat Disord* 2006;6:10. doi:10.1186/1472-6815-6-10.

For peer review only

SPIRIT — STANDARD PROTOCOL ITEMS: RECOMMENDATIONS FOR INTERVENTIONAL TRIALS

SPIRIT 2013 Checklist: Recommended items to address in a clinical trial protocol and related documents*

Section/item	Item Number	Page Number
**Administrative information**		
Title	1	3
Trial registration	2a	3
	2b	3
Protocol version	3	5
Funding	4	5
Roles and responsibilities	5a	5
	5b	6
	5c	6
	5d	n/a
**Introduction**		
Background and rationale	6a	7
	6b	9
Objectives	7	10
Trial design	8	11
**Methods: Participants, interventions, and outcomes**		
Study setting	9	12
Eligibility criteria	10	12
Interventions	11a	13
	11b	14
	11c	14
	11d	14

For peer review only

Outcomes	12	14
Participant timeline	13	17
Sample size	14	18
Recruitment	15	19
**Methods: Assignment of interventions (for controlled trials)**		
Allocation:		
Sequence generation	16a	19
Allocation concealment mechanism	16b	19
Implementation	16c	19
Blinding (masking)	17a	19
	17b	n/a
**Methods: Data collection, management, and analysis**		
Data collection methods	18a	20
	18b	20
Data management	19	20
Statistical methods	20a	20
	20b	21
	20c	22
**Methods: Monitoring**		
Data monitoring	21a	23
	21b	23
Harms	22	23
Auditing	23	23
**Ethics and dissemination**		
Research ethics approval	24	24
Protocol amendments	25	24
Consent or assent	26a	24
	26b	24
Confidentiality	27	25
Declaration of interests	28	25

Access to data	29	25
Ancillary and post-trial care	30	25
Dissemination policy	31a	26
	31b	26
	31c	26
**Appendices**		
Informed consent materials	32	Appendices.pdf
Biological specimens	33	Appendices.pdf

*It is strongly recommended that this checklist be read in conjunction with the SPIRIT 2013 Explanation & Elaboration for important clarification on the items. Amendments to the protocol should be tracked and dated. The SPIRIT checklist is copyrighted by the SPIRIT Group under the Creative Commons "Attribution-NonCommercial-NoDerivs 3.0 Unported" license.

For peer review only

BMJ Open

Evaluation of different infant vaccination schedules incorporating pneumococcal vaccination (the Vietnam Pneumococcal Project): protocol of a randomised controlled trial

Journal:	*BMJ Open*
Manuscript ID	bmjopen-2017-019795.R1
Article Type:	Protocol
Date Submitted by the Author:	25-Jan-2018
Complete List of Authors:	Temple, Beth; Menzies School of Health Research, Global Health Division; London School of Hygiene and Tropical Medicine, Epidemiology and Population Health<br>Toan, Nguyen; Pasteur Institute of Ho Chi Minh City, Department of Disease Control and Prevention<br>Uyen, Doan ; Pasteur Institute of Ho Chi Minh City, Department of Disease Control and Prevention<br>Balloch, Anne; Murdoch Childrens Research Institute, Pneumococcal Research<br>Bright, Kathryn; Murdoch Childrens Research Institute, Pneumococcal Research<br>Cheung, Yin Bun; Duke-NUS Medical School, Centre for Quantitative Medicine; University of Tampere and Tampere University Hospital, Centre for Child Health Research<br>Licciardi, Paul; Murdoch Childrens Research Institute, Pneumococcal Research; University of Melbourne, Department of Paediatrics<br>Nguyen, Cattram; Murdoch Childrens Research Institute, Pneumococcal Research; University of Melbourne, Department of Paediatrics<br>Phuong, Nguyen; Pasteur Institute of Ho Chi Minh City, Department of Disease Control and Prevention<br>Satzke, Catherine; Murdoch Childrens Research Institute, Pneumococcal Research; University of Melbourne, Department of Paediatrics<br>Smith-Vaughan, Heidi; Menzies School of Health Research, Child Health Division<br>Vu, Thi Que Huong; Pasteur Institute of Ho Chi Minh City, Microbiology and Immunology<br>Huu, Tran; Pasteur Institute of Ho Chi Minh City, Department of Disease Control and Prevention<br>Muholland , Kim; London School of Hygiene and Tropical Medicine, Epidemiology and Population Health; Murdoch Childrens Research Institute, Pneumococcal Research
**Primary Subject Heading**:	Global health
Secondary Subject Heading:	Immunology (including allergy), Epidemiology, Infectious diseases, Paediatrics
Keywords:	Clinical trials < THERAPEUTICS, Paediatric infectious disease &

	immunisation < PAEDIATRICS, MICROBIOLOGY, Epidemiology < INFECTIOUS DISEASES

SCHOLARONE™
Manuscripts

For peer review only

Title
Evaluation of different infant vaccination schedules incorporating pneumococcal vaccination (The Vietnam Pneumococcal Project): protocol of a randomised controlled trial

Corresponding author
Beth Temple
Menzies School of Health Research
PO Box 41096
Casuarina NT 0811
Australia
+61 8 8946 8600
beth.temple@menzies.edu.au

Co-authors

TEMPLE, Beth[1, 2]
TOAN, Nguyen Trong[4]
UYEN, Doan Y[4]
BALLOCH, Anne[3]
BRIGHT, Kathryn[3]
CHEUNG, Yin Bun[5, 6]
LICCIARDI, Paul[3, 7]
NGUYEN, Cattram Duong[3, 7]
PHUONG, Nguyen Thi Minh[4]
SATZKE, Catherine[3, 7]
SMITH-VAUGHAN, Heidi[8]
VU, Thi Que Huong[9]
HUU, Tran Ngoc[4]
MULHOLLAND, Edward Kim[2, 3]

[1]Global Health, Menzies School of Health Research, Darwin, Australia
[2]Epidemiology and Population Health, London School of Hygiene & Tropical Medicine, London, UK
[3]Pneumococcal Research, Murdoch Childrens Research Institute, Melbourne, Australia
[4]Department of Disease Control and Prevention, Pasteur Institute of Ho Chi Minh City, Ho Chi Minh City, Vietnam
[5]Centre for Quantitative Medicine, Duke-NUS Medical School, Singapore, Singapore
[6]Centre for Child Health Research, University of Tampere and Tampere University Hospital, Tampere, Finland
[7]Department of Paediatrics, University of Melbourne, Melbourne, Australia
[8]Child Health, Menzies School of Health Research, Darwin, Australia
[9]Microbiology and Immunology, Pasteur Institute of Ho Chi Minh City, Ho Chi Minh City, Vietnam

Word count: 6,406 words

For peer review only

ABSTRACT
Introduction: The World Health Organization (WHO) recommends the use of pneumococcal conjugate vaccine (PCV) as a priority. However there are many countries yet to introduce PCV, especially in Asia. This trial aims to evaluate different PCV schedules and to provide a head-to-head comparison of PCV10 and PCV13, in order to generate evidence to assist with decisions regarding PCV introduction. Schedules will be compared in relation to their immunogenicity and impact on nasopharyngeal carriage of *Streptococcus pneumoniae* and *Haemophilus influenzae*.
Methods and analysis: This randomised, single-blind controlled trial involves 1200 infants recruited at 2 months of age to one of six infant PCV schedules: PCV10 in a 3+1, 3+0, 2+1 or two-dose schedule; PCV13 in a 2+1 schedule; and controls that receive two doses of PCV10 and 18 and 24 months. An additional control group of 200 children is recruited at 18 months that receive one dose of PCV10 at 24 months. All participants are followed up until 24 months of age. The primary outcome is the post-primary series immunogenicity, expressed as the proportions of participants with serotype-specific antibody levels ≥0.35μg/mL for each serotype in PCV10.
Ethics and dissemination: Ethical approval has been obtained from the Human Research Ethics Committee of the Northern Territory Department of Health and Menzies School of Health Research (EC00153) and the Vietnam Ministry of Health Ethics Committee. The results, interpretation and conclusions will be presented to parents and guardians, at national and international conferences, and published in peer-reviewed open access journals.
Trial registration details: NCT01953510

Strengths and limitations of this study

- This study is specifically designed to address two independent questions within a single study: which schedule to use for the provision of PCV, and which PCV to use.
- This study includes a head-to-head comparison of the two licensed PCVs, allowing a direct assessment of their relative immunogenicity and impact on nasopharyngeal carriage.
- The primary outcome is the criteria used for the licensing and varying of PCV schedules.
- This study has relatively low power for the secondary nasopharyngeal carriage outcomes, so the ability to draw conclusions relating to these outcomes is vulnerable in the event of lower than anticipated carriage rates.

For peer review only

ADMINISTRATIVE INFORMATON

Title
Evaluation of different infant vaccination schedules incorporating pneumococcal vaccination (the Vietnam Pneumococcal Project).

Trial registration
ClinicalTrials.gov: NCT01953510

Trial registration - data set

Data category	Information
Primary registry and trial identifying number	ClinicalTrials.gov NCT01953510
Date of registration in primary registry	25 September 2013
Secondary identifying numbers	09/19, 10PN-PD-DIT-079
Source(s) of monetary or material support	National Health and Medical Research Council, Australia<br>Bill & Melinda Gates Foundation<br>GlaxoSmithKline Biologicals SA
Primary sponsor	Murdoch Childrens Research Institute, Australia
Contact for public queries	Professor Kim Mulholland<br>kim.mulholland@lshtm.ac.uk
Contact for scientific queries	Professor Kim Mulholland<br>kim.mulholland@lshtm.ac.uk
Public title	Trial of pneumococcal vaccine schedules in Ho Chi Minh City, Vietnam
Scientific title	Evaluation of different infant vaccination schedules incorporating pneumococcal vaccination (Vietnam Pneumococcal Project)
Countries of recruitment	Vietnam
Health condition(s) or problem(s) studied	Pneumococcal vaccination
Intervention(s)	Active Comparator A: PCV10 administered at 2, 3, 4 and 9 months of age (3+1)
	Experimental B: PCV10 administered at 2, 3 and 4 months of age (3+0)
	Experimental C: PCV10 administered at 2, 4 and 9 months of age (2+1)
	Experimental D: PCV10 administered at 2 and 6 months of age (2 dose)
	Experimental E: PCV13 administered at 2, 4 and 9 months of age (2+1 PCV13)
	Control F: No infant PCV vaccination; PCV10 administered at 18 and 24 months of age
	Control G: Recruited at 18 months of age, non-randomised; PCV10 administered at 24 months of age
Key inclusion and exclusion criteria	Inclusion:<br>• Aged between 2 months and 2 months plus 2 weeks (Arms A-F) or aged between 18m and 18m plus 4 weeks

For peer review only

	(Arm G)<br>• No significant maternal or perinatal history<br>• Born at or after 36 weeks gestation<br>• Written and signed informed consent from parent/legal guardian<br>• Lives within approximately 30 minutes of the commune health centre<br>• Family anticipates living in the study area for the next 22 months (Arms A-F) or 6 months (Arm G)<br>• Has received three doses of either Quinvaxem or Infanrix-hexa in infancy (Arm G)<br>•
	Exclusion:<br>• Known allergy to any component of the vaccine<br>• Allergic reaction or anaphylactic reaction to any previous vaccine<br>• Known immunodeficiency disorder<br>• Known HIV-infected mother<br>• Known thrombocytopenia or coagulation disorder<br>• On immunosuppressive medication<br>• Administration or planned administration of any immunoglobulin or blood product since birth<br>• Severe birth defect requiring ongoing medical care<br>• Chronic or progressive disease<br>• Seizure disorder<br>• History of invasive pneumococcal, meningococcal or *Haemophilus influenzae* type b diseases, or tetanus, measles, pertussis or diphtheria infections<br>• Receipt of any 2 month vaccines through the EPI program (Arms A-F), or receipt of PCV (Arm G);<br>Family plans on giving the infant the Quinvaxem (DTP-Hib-HBV) or OPV vaccines (Arms A-F)
Study type	Interventional, randomised, parallel group, open label phase II/III trial (Arms A-F). Non-randomised (Arm G). Outcomes assessors (laboratory) blinded. Purpose: prevention.
Enrolment period	Arms A-F: 30 September 2013 - 8 January 2015<br>Arm G: 14 April 2015 - 12 May 2016
Sample size	Target: 1400<br>Number enrolled: 1400
Recruitment status	Active, not recruiting
Primary outcome	Proportion of children with IgG antibody concentration ≥0.35μg/mL for individual pneumococcal serotypes, four weeks post-primary series, measured by ELISA
Key secondary outcomes	Geometric mean concentration (GMC) of serotype-specific IgG, four weeks post-primary series, measured by ELISA
	Proportion of children with IgG antibody concentration ≥0.35μg/mL and GMCs, four weeks post-booster, measured by ELISA
	Proportion of children with serotype-specific opsonisation indices ≥8, four weeks post-primary series and four weeks post-booster, measured by opsonophagocytic assay
	Median number of serotype-specific antibody secreting memory B cells, four weeks post-booster and at 18 months of

For peer review only

	age, measured by ELISPOT
	Proportion of children carrying pneumococcus (any pneumococci, capsular pneumococci, or vaccine-type pneumococci) in the nasopharynx at 12 months of age, measured by culture and latex agglutination serotyping
	Proportion of children carrying NTHi in the nasopharynx at 12 months of age, measured by culture and PCR
Ethics Review	Approved by the Human Research Ethics Committee of the Northern Territory Department of Health and Menzies School of Health Research (EC00153) and the Vietnam Ministry of Health Ethics Committee

Protocol version

Protocol version 10.0 dated 3 June 2015 with Letter of Amendment Number 1 dated 1 September 2016

Revision chronology

Original: Version 3.1, 5 June 2013

First amendment: Version 5.0, 21 April 2014. Main reason for amendment: the Vietnam Ministry of Health (MOH) does not permit the co-administration of measles vaccine and *Infanrix-hexa* vaccine. Measles, *Infanrix-hexa* and PCV were scheduled to be given at 9 months of age in Arms C and E. An additional visit at 9.5 months of age was added for these groups, for receipt of PCV and *Infanrix-hexa*.

Second amendment: Version 7.0, 8 December 2014. Main reason for amendment was that additional funding was secured to: extend the follow up of all participants from 18 to 24 months of age; evaluate a single dose of PCV10 at 18 months of age; and recruit an additional control group at 18 months of age (Arm G) to provide a comparator for the original control group (Arm F). Version 7.0 was never implemented (see below).

Third amendment: Version 9.0, 4 March 2015. Main reason for amendment: to incorporate minor clarifications to version 7.0 requested during review by MOH. These changes did not affect participant recruitment or follow-up and the version number was only changed at the request of MOH.

Fourth amendment: Version 10.0, 3 June 2015. Main reason for amendment: to incorporate additional minor clarifications to version 9.0 requested during review by the Vietnam Ministry of Health. These changes did not affect participant recruitment or follow-up and the version number was only changed at the request of MOH.

Roles and responsibilities

Sponsor contact information

Trial Sponsor: Murdoch Childrens Research Institute, Royal Children's Hospital, Flemington Road, Parkville, Victoria 3052, Australia

Telephone: +61 3 8341 6200

Contact name: Professor Kim Mulholland

Sponsor and funder

GSK was consulted during the design of the trial. None of the funders have any role in the trial conduct, trial management, laboratory tests, or data analyses.

For peer review only

INTRODUCTION

Background and rationale

Streptococcus pneumoniae (pneumococcus) remains a leading vaccine preventable cause of serious infection in young children, despite the availability of effective vaccines. The first infant pneumococcal vaccine, the 7-valent pneumococcal conjugate vaccine (PCV7), was licensed in the United States in the year 2000. Introduction of PCV7 has been associated with dramatic reductions in pneumococcal disease.[1-3] However, geographical variation in serotype distribution[4-7] and an increase in invasive pneumococcal disease (IPD) caused by non-PCV7 serotypes following vaccine introduction[8] necessitated the development of higher valency PCVs.

There are currently two licensed PCVs: PCV10, a 10-valent pneumococcal vaccine that uses non-typeable *Haemophilus influenzae* (NTHi) protein D as a carrier protein for eight of the ten serotypes (*Synflorix*™, PHiD-CV, GSK); and PCV13, a 13-valent pneumococcal CRM_{197} conjugate vaccine (*Prevnar-13*™/*Prevenar-13*™, Pfizer). Both have been shown to be non-inferior to PCV7 in terms of post-primary series immunogenicity for the shared serotypes.[9-11] Despite the availability of both PCV10 and PCV13 for several years, there have been no published studies to date directly comparing their post-primary series immunogenicity or impact on nasopharyngeal (NP) carriage.

The cost of PCVs is a major barrier to vaccine introduction in low to middle-income countries; therefore investigation of alternative schedules with a reduced number of doses is of great importance. The uptake of PCV introduction in Asia has been particularly slow. Three schedules are currently in routine use around the world for PCV introduction: a 3+1 schedule (a three-dose primary series followed by a booster dose in the second year of life); a 3+0 schedule (a three-dose primary series without a booster dose); and a 2+1 schedule (a two-dose primary series followed by a booster dose in the second year of life). Data from periods of PCV7 shortage in the United States show high vaccine effectiveness of a two-dose primary series against invasive pneumococcal disease (IPD),[12 13] and trial data of CRM_{197}-conjugated PCVs show comparable immunogenicity following a two- or three-dose primary series, although antibody levels to serotypes 6B and 23F tend to be lower after two doses.[14 15] Trials of PCV10 and PCV13 also support the use of a two-dose

For peer review only - http://bmjopen.bmj.com/site/about/guidelines.xhtml

primary series. A trial of PCV10 in Europe directly comparing the immunogenicity of a two- and three-dose primary series showed a similar proportion of participants achieving protective antibody levels (≥0.2μg/mL) for all ten serotypes.[16] In a trial of PCV13 in Mexico, over 93% of participants achieved protective antibody levels (≥0.35μg/mL) for most of the 13 serotypes following two doses, with the exception of serotypes 6B and 23F.[17] Four trials in Europe directly comparing PCV13 and PCV7 responses showed comparable immune responses between the vaccines following two doses.[18]

In developing countries, a 2+1 schedule with a booster dose in the first year of life may be advantageous. This modified schedule would likely increase compliance, would provide full immunisation closer to the peak incidence of pneumococcal disease, and could enable the booster dose to coincide with measles vaccination. Alternatively, a further reduced PCV schedule with only two doses may be optimal for pneumococcal vaccination. Our previous trial in Fiji showed that protective antibody levels were reached for five of the seven serotypes following a single dose of PCV7 at 14 weeks of age.[15] Furthermore, a booster dose of the 23-valent pneumococcal polysaccharide vaccine at 12 months of age was more immunogenic following a single dose primary series of PCV7 compared with a two or three dose primary series for four serotypes, and comparable for the other three serotypes.[19] A trial of PCV9 from South Africa also showed that one dose at six weeks of age elicited a significant response for seven serotypes,[20] and modelling data from the US suggest that a single dose of PCV could prevent up to 62% of IPD.[21] More recently, in the UK, where routine infant PCV vaccination has been in place for over 10 years, a 1+1 schedule of PCV13 was shown to elicit equivalent or superior post-booster responses compared with a 2+1 schedule for nine serotypes.[22]

Carriage of pneumococci in the nasopharynx is commonly a prerequisite for IPD, and is the usual means of transmission of the bacteria. The herd effect of pneumococcal vaccination is mediated by the impact on NP carriage.[23] Vaccination with PCVs generally results in a decrease in vaccine type (VT) pneumococcal carriage, which is most commonly observed after a booster dose and often accompanied by a compensatory increase in non-VT carriage.[23-27] There have been few trials that evaluate the effect of different PCV schedules on carriage. A trial from the Netherlands showed that a two-dose primary series with or without a booster reduced VT carriage at 12 months of age compared with controls.[28] VT carriage was further reduced at 18 months in the group that received the booster dose,

For peer review only

compared with the group that did not receive the booster, although this difference did not persist at 24 months of age. Similarly, our trial in Fiji showed that a two or three dose primary series with or without a booster reduced VT carriage at 12 months of age compared with controls, but no difference was seen at 17 months of age (F Russell, personal communication).

It has been hypothesised that the Protein D carrier in PCV10 may result in an impact on *H. influenzae* carriage. A recent review of the impact of Protein D-containing PCVs on NTHi carriage concludes that any such impact is likely to be small and transient, although changes in the density of carriage are yet to be evaluated. Two large phase III trials (POET trial of an 11-valent PCV and COMPAS trial of PCV10) showed trends towards a reduction in NTHi carriage following a booster dose of PCV, along with a trial of PCV10 in toddlers in Kenya; but other trials conducted in Finland, the Netherlands and the Czech Republic showed no impact of PCV10 on NTHi carriage.[29]

This trial includes six infant vaccination schedules: four different PCV10 schedules (Arm A, a 3+1 schedule at 2, 3, 4 and 9 months of age; Arm B, a 3+0 schedule at 2, 3 and 4 months; Arm C, a 2+1 schedule at 2, 4 and 9 months; and Arm D, a 2-dose schedule at 2 and 6 months); a 2+1 PCV13 schedule at 2, 4 and 9 months (Arm E); and a control group that receives two doses of PCV10 at 18 and 24 months (Arm F). In response to more recent interest in schedules with only one or two doses of PCV, which may be sufficient to maintain herd immunity at the population level, an additional control group is recruited at 18 months of age for comparison with the initial control group (Arm G).

Explanation for choice of comparators

There was no PCV licensed in Vietnam at the time the protocol was finalised in 2013. The inclusion of control groups that receive no infant doses of PCV is therefore justified. Control group participants recruited in infancy receive two doses of PCV10, at 18 and 24 months of age. Control group participants recruited at 18 months of age receive a single dose of PCV10 at 24 months of age. Intervention group participants receive at least two doses of PCV in the first year of life. All participants receive pneumococcal immunisation that is likely to be effective and is not otherwise available in Vietnam. The specific regimens to be evaluated are based on likely future global recommendations and to directly compare the two licensed PCVs.

For peer review only

Both PCV10 and PCV13 have been shown to be non-inferior to PCV7 for the serotypes common to both vaccines, and to have the potential to provide protection against the additional serotypes included.[9-11] For both vaccines the most common adverse reactions are redness at the injection site and irritability, which are common following administration of other vaccines. Other adverse reactions may include: drowsiness; temporary loss of appetite; pain, redness or swelling at the injection site; and fever. Such reactions are usually temporary.

Objectives

This trial has been designed to answer two independent questions concurrently, relating to the evaluation of different schedules incorporating PCV10 and the comparison of PCV10 and PCV13:

1) What is the optimal schedule for provision of EPI vaccines with the incorporation of PCV10; and
2) How do the responses to vaccination with PCV10 or PCV13 compare?

The primary endpoint for both study questions is the post-primary series immunogenicity. For this endpoint data from Arms A and B are combined, as they receive an identical three-dose primary series (see Table 1 for a detailed description of the trial arms). The primary analysis for each study question is to assess non-inferiority of the post-primary series immunogenicity (in terms of the proportion of participants achieving protective levels of serotype-specific IgG of ≥0.35μg/mL), using Arms A+B as the comparator (see below for details). Non-inferiority is assessed for each of the ten serotypes in PCV10, and an overall conclusion of non-inferiority drawn if found for at least seven of the ten serotypes.

1) What is the optimal schedule for provision of Expanded Program of Immunisation (EPI) vaccines with the incorporation of PCV10?

Primary objective

The primary objective is to compare a 2+1 schedule at 2, 4 and 9 months of age with a 3+1 schedule at 2, 3, 4 and 9 months of age. The primary hypothesis is that the proportion of participants with protective levels of antibody is non-inferior following a two-dose primary series (Arm C) compared with a three-dose primary series (Arms A+B). The schedules will also be compared in relation to: the Geometric Mean

For peer review only - http://bmjopen.bmj.com/site/about/guidelines.xhtml

Concentrations (GMCs) of IgG and opsonophagocytosis post-primary series; the proportion of participants with protective levels of antibody, the GMCs of IgG and opsonophagocytosis post-booster; the memory B cell responses; and the impact on nasopharyngeal (NP) carriage rates and density of bacteria of interest.

Key secondary objectives

- To investigate an experimental two-dose schedule at 2 and 6 months of age (Arm D), compared with a 3+1 schedule (Arm A+/-B) and a 2+1 schedule (Arm C);
- To assess the impact of a booster dose on NP carriage of pneumococcus and NTHi, comparing a 3+1 schedule (Arm A) with a 3+0 schedule (Arm B) and with unvaccinated controls (Arm F); and
- To evaluate a single dose of PCV10 at 18 months of age (Arm F), compared with unvaccinated controls (Arm G).

2) How do the responses to vaccination with PCV10 or PCV13 compare?

Primary objective

The primary objective is to compare a PCV13 schedule at 2, 4 and 9 months of age with a PCV10 schedule at 2, 3, 4 and 9 months of age. The primary hypothesis is that the proportion of participants with protective levels of antibody is non-inferior following a two-dose primary series of PCV13 (Arm E) compared with a three-dose primary series of PCV10 (Arms A+B). The schedules will also be compared in relation to: the GMCs of IgG and opsonophagocytosis post-primary series; the proportion of participants with protective levels of antibody, the GMCs of IgG and opsonophagocytosis post-booster; the memory B cell responses; and the impact on nasopharyngeal (NP) carriage rates and density of bacteria of interest.

Key secondary objectives

- To compare PCV10 (Arm C) and PCV13 (Arm E) in a 2+1 schedule at 2, 4 and 9 months of age; and
- To compare the responses to a single dose of PCV10 (Arm D) and PCV13 (Arm E).

For peer review only - http://bmjopen.bmj.com/site/about/guidelines.xhtml

Additional objectives

Additional objectives relating to the second control group (Arm G) are:

- To evaluate a single dose of PCV10 at 18 months of age, comparing serotype-specific antibody levels in Arms F and G at 18, 19 and 24 months of age; and
- To compare the immunogenicity and reactogenicity of *Infanrix-hexa* at 18 months of age in children who have received 3 doses of *Infanrix-hexa* or *Quinvaxem* in infancy (Arm G).

Trial design

The Vietnam Pneumococcal Project is a single-blind, open-label, randomized controlled phase II/III non-inferiority trial to investigate simplified childhood vaccination schedules that are more appropriate for developing country use. This is a seven-arm trial that includes six different infant vaccination schedules (Arm A-F) and an additional control group (Arm G) recruited at 18 months of age (Table 1). Arm A receives PCV10 at 2, 3, 4 and 9 months of age (3+1); Arm B receives PCV10 at 2, 3 and 4 months (3+0); Arm C receives PCV10 at 2, 4 and 9 months (2+1); Arm D receives PCV10 at 2 and 6 months (2-dose); Arm E receives PCV13 at 2, 4 and 9 months (2+1); Arm F receives two doses of PCV10 at 18 and 24 months; and Arm G receives one dose of PCV10 at 24 months. Participants also receive *Infanrix-hexa* (DTP-Hib-HBV-IPV) instead of the routine EPI vaccine *Quinvaxem* (DTP-Hib-HBV): four doses for participants in Arms A-F and one dose for Arm G participants.

For peer review only

METHODS AND ANALYSIS

Study setting

PCV introduction in Asia has been slow, in part due to a lack of local or regional data on the effect of PCV. We selected the Southeast Asian country of Vietnam as the location for the trial as a country with a strong health system, a track record of conducting relevant clinical trials, and a Government with strong interest both in the trial and in introducing PCV in the near future. Furthermore, trial results from Vietnam are likely to be considered as applicable to other countries in the region. This is the first trial involving infants to take place within Ho Chi Minh City, the largest city in Vietnam. The trial is conducted in two districts, District 4 and District 7. Districts are divided into communes, each of which has a health centre that provides preventive health services including EPI immunizations, along with some primary health care services. The study is conducted in one commune health centre in each district, with participants drawn from the surrounding communes within that district.

Eligibility criteria

Inclusion criteria

Subjects must meet all of the following inclusion criteria in order to be eligible to participate: aged between 2 months and 2 months plus 2 weeks (Arms A-F) or aged between 18 months and 18 months plus 4 weeks (Arm G); no significant maternal or perinatal history; born at or after 36 weeks gestation; written informed consent from the parent/legal guardian; lives within approximately 30 minutes of the commune health centre; anticipates living in the study area for the next 22 months (Arms A-F) or 6 months (Arm G); and received 3 doses of either *Quinvaxem* or *Infanrix-hexa* in infancy (Arm G only).

Exclusion criteria

Subjects meeting any of the following exclusion criteria at baseline will be excluded from study participation: known allergy to any component of the vaccine; allergic or anaphylactic reaction to any previous vaccine; known immunodeficiency disorder; known HIV-infected mother; known thrombocytopenia or coagulation disorder; on immunosuppressive medication; administration or planned administration of any immunoglobulin or blood product since birth; severe birth defect requiring ongoing medical care; chronic or progressive disease; seizure disorder; history of invasive pneumococcal, meningococcal or *H. influenzae* type b diseases, or tetanus,

For peer review only - http://bmjopen.bmj.com/site/about/guidelines.xhtml

measles, pertussis or diphtheria infections; receipt of any 2 month vaccines through the EPI program (Arms A-F), or receipt of PCV (Arm G); or family plans on giving the infant *Quinvaxem* (Arms A-F).

Interventions

PCV schedules

Eligible participants recruited in infancy are randomised to one of six different vaccination schedules (Table 1). Participants randomised to Arms A-D receive PCV10 in a: 3+1 schedule at 2, 3, 4 and 9 months of age; a 3+0 schedule at 2, 3 and 4 months of age; a 2+1 schedule at 2, 4 and 9 months of age; or a two-dose schedule at 2 and 6 months of age, respectively. Participants randomised to Arm E receive PCV13 in a 2+1 schedule at 2, 4 and 9 months of age. Control group participants receive PCV10 at 18 and 24 months of age if randomised to Arm F, or PCV10 at 24 months of age if recruited to Arm G at 18 months of age. PCV is administered by intramuscular injection into the anterolateral thigh in children less than 18 months old and in the deltoid muscle of the arm in children aged 18 months and over. All vaccinations are performed by nurses specifically trained in infant vaccine administration.

PCV10

PCV10 (*Synflorix*) is a 10-valent pneumococcal polysaccharide conjugate vaccine using Protein D (a highly conserved surface protein from NTHi) as the main carrier protein. PCV10 is presented as a turbid white suspension in a two-dose vial. One dose consists of 0.5mL of the liquid vaccine, containing 1μg of pneumococcal polysaccharide from serotypes 1, 5, 6B, 7F, 9V, 14 and 23F and 3μg of pneumococcal polysaccharide from serotypes 4, 18C and 19F. Serotypes 1, 4, 5, 6B, 7F, 9V, 14 and 23F are conjugated to Protein D; serotype 18C is conjugated to tetanus toxoid carrier protein; and serotype 19F is conjugated to diphtheria toxoid carrier protein.

PCV13

PCV13 (*Prevnar-13*) is a 13-valent pneumococcal polysaccharide conjugate vaccine using non-toxic diphtheria CRM_{197} carrier protein. PCV13 is presented as a 0.5mL suspension in a single-dose pre-filled syringe. One dose contains approximately 2.2μg of pneumococcal polysaccharide from serotypes 1, 3, 4, 5, 6A, 7F, 9V, 14,

For peer review only - http://bmjopen.bmj.com/site/about/guidelines.xhtml

18C, 19A, 19F and 23F and 4.4μg of pneumococcal polysaccharide from serotype 6B.

Criteria for discontinuing or modifying allocated interventions

There is no modification of doses for participants in this study. If a participant has an allergic or anaphylactic response to vaccination they will be withdrawn from the study. Participants may also be withdrawn voluntarily by the parent/legal guardian at any time, or by the study staff if they refuse any further study procedures or develop any of the exclusion criteria during the course of the study.

Strategies to improve and monitor adherence

Scheduled visit dates are noted on a health record card kept by the parent. If a participant does not attend a scheduled visit, a reminder phone call is made from the study clinic. If the participant cannot be contacted directly, their local Commune Health Centre is contacted for further follow up by phone or by home visit.

Relevant concomitant care

Participants receive *Infanrix-hexa*, which is only available on the private market, instead of the routine EPI vaccine *Quinvaxem*. Participants in Arms A-F receive four doses in one of the following schedules: 2, 3, 4 and 19 months (Arms A and B); 2, 4, 9.5 and 19 months (Arms C and E); 2, 4, 6 and 19 months (Arm D); or 2, 3, 4 and 18 months (Arm F); and participants in Arm G receive one dose at 18 months of age. The routine EPI measles and measles-rubella immunisations are also provided during the course of the study: measles at 9 months of age and measles-rubella at 18 (Arms A-E) or 19 (Arms F-G) months of age. Participants allocated to one of the 2+1 vaccination schedules (Arms C and E) receive measles at 9 months of age and receive PCV and *Infanrix-hexa* two weeks later. For visits with two vaccinations, the vaccines are administered in different limbs. Other vaccinations are permitted in this study with a two-week interval from study vaccines, with the exception of *Quinvaxem* in Arms A-F. Other medications are also permitted, with the exception of immunosuppressive medication and medications listed as contraindicated to the study vaccines.

For peer review only - http://bmjopen.bmj.com/site/about/guidelines.xhtml

Outcomes

Primary outcome measure

The primary outcome measure is the concentration of serotype-specific IgG for the ten serotypes common to both PCV10 and PCV13, assessed four weeks post-primary series and measured using a modified 3rd generation standardized ELISA.[30] Primary comparisons between arms are made in terms of the proportion of children with antibody concentration ≥0.35µg/mL for individual serotypes. The cut-off of 0.35µg/mL was determined as a result of a pooled analysis of data from efficacy trials,[31] and is used as the basis for non-inferiority assessments for the approval of new PCVs.[32-34]

Secondary immunogenicity outcome measures

- Serotype-specific IgG antibody concentrations for all PCV13 serotypes are measured by ELISA from all blood samples (Table 1) and are summarised in terms of both the proportion of children with antibody concentration ≥0.35µg/mL and the GMC.
- Opsonisation indices (OI) for all PCV13 serotypes are measured by opsonophagocytic assay (OPA)[35] for 100 participants per intervention group (Arms A-E) four weeks post-primary series and four weeks post-booster, and are summarised in terms of the proportion of participants with OI ≥8 and the Geometric Mean Titre (GMT).
- Polysaccharide specific memory B cells for serotypes 1, 5, 6B, 14, 18C, 19A and 23F are enumerated by ELISPOT[35] for 50 participants per intervention group (Arms A-E) post-booster and at 18 months of age, and for 100 participants per control group (Arms F and G) at 18 and 24 months of age. The results are summarised as the median number of antibody secreting cells.

Nasopharyngeal carriage outcome measures

- NP carriage of pneumococcal serotypes is measured by traditional culture (colonial morphology, α-haemolysis, the optochin test and *lytA* PCR where indicated)[36] and latex agglutination using type-specific antisera at 2, 6, 9 and 12 months of age in all groups and at 18 and 24 months of age in the control groups (Arms F and G). NP carriage and density of pneumococcal serotypes are measured by quantitative real-time PCR (qPCR) targeting *lytA* and microarray at 18 and 24 months of age.[37 38] Overall, capsular, vaccine-type and serotype-specific carriage rates are described. The antimicrobial resistance of

For peer review only

pneumococcal isolates is determined at 12 months of age by CLSI disk diffusion, for oxacillin, erythromycin, trimethoprim/sulphamethoxazole, ofloxacin, clindamycin, vancomycin, tetracycline, and chloramphenicol. E-tests are conducted for penicillin, ceftriaxone, and vancomycin where indicated, and CLSI breakpoints applied.

- NP carriage of *H. influenzae* is measured by traditional culture (colonial morphology, X and V dependence, *SiaT* PCR for discrimination from *H. haemolyticus*, and the Phadebact® Haemophilus coagglutination test) at 12 months of age in all groups, at 6 and 9 months of age in Arms A and C, and from all swabs in the control groups (Arms F and G). Overall density of *H. influenzae* carriage is measured by qPCR targeting *hpd* and *SiaT* diagnostic targets at 18 and 24 months of age.[39 40]

Immunogenicity of *Infanrix-hexa*

Immunogenicity of *Infanrix-hexa* is measured in terms of IgG levels to diphtheria, tetanus, Hib PRP antigen, hepatitis B surface antigen, and *B. pertussis* (PT). IgG levels will be determined by ELISA, using commercial test kits.

An overview of the procedures for collection, transportation and laboratory analyses of the blood and NP samples can be found in Appendix 1.

For peer review only

Participant timeline

Table 1: Schedule of enrolment, interventions and assessments

**Age (months)**	**2m**	**3m**	**4m**	**5m**	**6m**	**7m**	**9m**	**9.5m**	**10m**	**12m**	**18m**	**19m**	**24m**
ENROLMENT:													
Informed consent	X										X[1]		
Eligibility assessment	X										X[1]		
Allocation	X												
INTERVENTIONS:													
PCV10 - Group A	X	X	X				X						
PCV10 - Group B	X	X	X										
PCV10 - Group C	X		X					X					
PCV10 - Group D	X				X								
PCV13 - Group E	X		X					X					
PCV10 - Group F											X		X
PCV10 - Group G													X
ASSESSMENTS:													
Demographics	X										X[1]		
Household characteristics	X										X[1]		
Nasopharyngeal swab	X				X		X			X	X		X
Blood sample - Group A	X[2]			X			X		X		X[2]		
Blood sample - Group B				X	X		X[2]		X		X[2]		
Blood sample - Group C				X	X[2]		X		X		X[2]		
Blood sample - Group D		X			X	X	X[2]				X[2]		
Blood sample - Group E		X[2]		X			X		X		X[2]		
Blood sample - Group F											X	X	X
Blood sample - Group G											X	X	X
General health	X	X	X	X	X	X	X	X	X	X	X	X	X

[1] Group G only. Any events occurring before 18m do not apply to Group G.
[2] Each participant provides only one of these blood samples (the last 50 participants per group enrolled into Groups A-E provide this blood sample at 18 months)

For peer review only - http://bmjopen.bmj.com/site/about/guidelines.xhtml

Sample size

The target sample size for infant recruitment (Groups A-F) is 1200 with an allocation ratio of 3:3:5:4:5:4, resulting in target group sizes of: A=150, B=150, C=250, D=200, E=250 and F=200. An additional target of 200 children aged 18 months are recruited into Group G. Sample size calculations are based on the primary outcome of post-primary series immunogenicity (proportion of participants with serotype-specific antibody concentrations ≥0.35μg/mL) for each of the two study questions. A non-inferiority margin of 10% difference in absolute risk is deemed clinically significant, as used by regulatory authorities. Non-inferiority is assessed for each of the ten serotypes in PCV10 (comparing Groups A+B with Group C or Group E), and an overall conclusion of non-inferiority is drawn if the alternative hypotheses are accepted for at least seven of the ten serotypes. This sample size provides >99% power for the overall conclusion of non-inferiority with a 5% one-sided type I error rate, estimated by simulation using a tailor-made program written for implementation in Stata with 10,000 replications.[41] Powers for serotype-specific hypotheses range from 83% to >99%, calculated in PASS Software 2002 using the Farrington-Manning (1990) method.[42] Based on findings from our earlier work in Fiji and from data available in the literature,[43-4543-45] the assumed probabilities of antibody concentration ≥0.35μg/mL are: 95% for serotypes 1, 4, 5, 7F, 9V, 14 and 19F; 90% for serotype 18C; 80% for serotype 23F; and 75% for serotype 6B. The within-subject correlation between the multiple binary endpoints is captured by a subject-level variation term with standard deviation 1.7 in a random-effect logistic regression model, and the loss to follow up rate is assumed to be 5% post-primary series and 10% at 12 months of age. The sample size also provides 98% power to detect a difference in post-primary series immunogenicity following two doses of PCV10 or PCV13, defined by a 10% difference in absolute risk based on a Fisher's Exact test (5% two-sided).

Carriage outcomes: The sample size provides 76% and 71% power to detect a difference in NTHi carriage rates at 12 months of age between Groups A and F and Groups A and B, respectively, and 64% and 59% power to detect a difference in vaccine-type pneumococcal carriage rates between Groups A and F and Groups A and B, respectively. Difference in carriage is defined by a relative risk of 0.6. The calculations were based on Fisher's Exact tests (5% one-sided), assuming carriage rates in Group F (controls) of 30% for NTHi and 24% for vaccine-type pneumococci, based on data from Vietnam (L Yoshida, personal communication).

For peer review only

Recruitment

Participants in Groups A-F are recruited from infants born in the study communes during the enrolment period. Commune health centre staff identify potential participants from the commune health centre birth records. Based on the expected number of births, around a quarter of infants born in the study communes need to be enrolled to complete recruitment within the target enrolment period of 12 months. Recruitment rates will be monitored on a monthly basis and meetings held with study staff and commune health centre staff to discuss any significant declines in recruitment rates. Commune health centre staff visit the home of potential participants when the infant is approximately six weeks old and provide verbal and written information about the trial, in Vietnamese. Those interested in participating are referred to the study clinic when the infant is approximately two months old. At this time, written informed consent is obtained (Appendix 2), after which a study nurse/doctor examines the infant to ensure that all the eligibility criteria are met. Participants in Group G are recruited from children turning 18 months old in the study communes in parallel to the children in Groups A-F turning 18 months.

Allocation

The allocation sequence for Groups A-F is produced using a computer-generated list of random numbers using a block randomisation scheme, stratified by district. The group allocation is contained within a sealed envelope at the study clinic, with sequential ID numbers written on the outside of the envelope. The allocation sequence is generated at Menzies School of Health Research. A study doctor will enrol participants and assign them to a study group by selecting the next available envelope. The envelope is not opened until after completion of the informed consent and eligibility assessment processes.

Blinding

All laboratory staff are blinded to the study group allocation as the key outcome measures that address the study objectives are all laboratory based. Laboratory samples are labelled with the ID number, which does not identify the study group. Given the different timing of the vaccination schedules in the different groups, the study nurses, vaccine administrators and participants will not be blinded to the study group allocation.

For peer review only

Data collection methods

Standardised carbon copy data collection forms are used and are completed by dedicated, trained study staff. The original is transported to the trial office for data entry, with the carbon copy filed at the clinic. Blood samples and NP swabs are collected by staff specifically trained in the collection of samples from infants, and the volume of blood collected and the swab quality are recorded.

Retention: Appointments are documented on a parent-held health record card and participants are given a small payment towards the transport costs of coming to the clinic for each study visit. Participants who miss a study visit will continue to be followed up for both sample collection and vaccine administration where possible, with attempts made to contact such participants until such time as they would have completed the study.

Data management

Data collection forms are double-entered by dedicated data entry staff into pre-coded EpiData version 3.1 files with built in range and consistency checks. Entered data are validated monthly and then uploaded to a central Microsoft Access database, stored on a secure server. Immunology results are double-entered in a Microsoft Excel spreadsheet. NP culture results are entered in a Microsoft Access database and qPCR and microarray results exported from SentiNET into a Microsoft Excel database. The data collection forms and laboratory results are linked at the time of analysis.

Statistical methods

Analysis of primary and secondary outcomes

For each of the two study questions, the primary objective is to compare a 2+1 schedule of 1) PCV10 and 2) PCV13, with a 3+1 schedule of PCV10. The primary outcome is the proportion of participants with serotype-specific antibody concentrations ≥0.35µg/mL, four weeks post-primary series (at 5 months of age). Data from Arms A and B are combined to form the three-dose post-primary series group. The primary analyses assess the non-inferiority of: 1) two doses of PCV10 at 2 and 4 months of age (Arm C) compared with three doses at 2, 3 and 4 months of age (Arms A+B); and 2) two doses of PCV13 at 2 and 4 months of age (Arm E) compared with three doses of PCV10 at 2, 3 and 4 months of age (Arms A+B). The proportion of children achieving protective levels of serotype-specific IgG (≥0.35µg/ml) four weeks post-primary series is determined for each of the ten PCV10

For peer review only - http://bmjopen.bmj.com/site/about/guidelines.xhtml

serotypes. The non-inferiority margin is defined by a 10% difference in absolute risk. The serotype-specific risk differences (Arm A+B - Arm C) with 90% CIs are calculated using the Newcombe Score method, and the null hypothesis rejected if the upper bound of the CI is <10%. Overall non-inferiority is declared if at least seven of the ten individual null hypotheses are rejected at one-sided 5% level of significance. Secondary data analyses to address the primary objective include the ratio of GMCs post-primary series (Arm C / Arms A+B and Arm E / Arms A+B) with 95% CIs, and the booster response analysed by ANCOVA, adjusting for pre-booster levels.

Analysis of key secondary objectives for study question 1:

- A single dose of PCV10 at 2 months of age (Arm D) will be assessed for non-inferiority to three doses at 2, 3 and 4 months of age (Arms A+B), as described for the primary objective
- The impact of a booster dose on pneumococcal and NTHi carriage will be assessed at 12 months of age. Overall pneumococcal, capsular pneumococcal, PCV10 type (with/without 6A and 19A) and NTHi carriage rates will be determined. Proportions will first be compared between the 3+1 group (Arm A) and the control group (Arm F), using Fisher's Exact test. Where significant differences are found, rates will then be compared between the 3+0 group (Arm B) and controls and between the 3+1 and 3+0 groups.

Analysis of key secondary objectives for study question 2:

- The immunogenicity of two doses of PCV10 or PCV13 will be compared in relation to the proportion of participants with serotype-specific antibody concentrations ≥0.35μg/mL (to the ten shared serotypes), four weeks post-primary series (at 5 months of age). A significant difference will be indicated by a 10% difference in absolute risk, comparing PCV10 (Arm C) with PCV13 (Arm E), and an overall difference will be declared if at least 7 of the 10 individual null hypotheses are rejected and the 7 differences are in the same direction.
- The immunogenicity of a single dose of PCV10 or PCV13 will be compared, as described for the immunogenicity of two doses.

Additional analyses

Descriptive analyses at the group level will be conducted on the OPA, ELISPOT and microarray data.

For peer review only - http://bmjopen.bmj.com/site/about/guidelines.xhtml

Populations of analysis

Analyses will be on a per-protocol population. The primary non-inferiority analyses will be repeated on an intention-to-treat population (ITT), with all participants analysed in the group they were randomised to. Any differences between the per-protocol and ITT analyses will be reported. For each outcome, all available data will contribute to the analyses. To investigate whether data are missing completely at random, we will explore whether attrition varies across the study arms based on baseline covariates. If differential attrition is dependent on baseline variables, we will use a modelling approach to adjust for any such baseline factors and we will present the adjusted results along with the primary analysis.

Additional populations of analysis

- OPAs will be conducted on a subset of 100 participants per group. The first 100 participants per group with both post-primary series and post-booster blood samples available will contribute to the OPA analysis.
- B cell assays will be conducted on a subset of 50 participants per group for Arms A-E and 100 participants per group for Arms F and G. The last 50/100 participants enrolled per group will have blood samples collected for the B cell analysis.

Further details of the planned statistical methods are found in the Statistical Analysis Plan, located on a secure server at MCRI.

Data monitoring

Data monitoring committee: Safety oversight is under the direction of an independent Data Safety and Monitoring Board (DSMB), in accordance with a DSMB Charter kept in the trial office. The DSMB will meet approximately three times a year to review aggregate and individual participant data related to safety, data integrity and overall conduct of the trial, including a detailed review of all Serious Adverse Events (SAEs). Interim analyses and stopping guidelines: No interim analyses are planned. Stopping guidelines are based on safety. An extraordinary meeting of the DSMB will be called in the event that serious safety issues emerge, to provide recommendations regarding termination of the trial. A final decision to terminate rests with the Principal Investigators and the Sponsor.

For peer review only

For peer review only - http://bmjopen.bmj.com/site/about/guidelines.xhtml

Harms

Data on SAEs will be collected throughout the study, with parents asked about hospitalisations and significant signs and symptoms at each study visit and through a regular review of hospital records. Details of any SAEs will be recorded on the standard reporting form from the Vietnam Ministry of Health and reported to the Principal Investigators and the Ethics Committees. Participants will be kept under observation for 30 minutes following vaccine administration to monitor for any adverse reactions, and information on reactogenicity in the 72 hours following vaccine administration will be recorded on parent held diary cards.

Auditing

External site monitoring will be provided by FHI360, to independently assess protocol and GCP compliance. Monitoring visits will occur at study initiation, close-out and approximately twice a year in each study clinic. 100% of Informed Consent Forms and SAEs and a random selection of approximately 20% of participant folders will be monitored, along with the Trial Regulatory File and laboratory records.

ETHICS AND DISSEMINATION

Research ethics approval

The protocol, the Plain Language Statement (PLS) and the Informed Consent Form (ICF) have approval from the Institutional Review Board at the Pasteur Institute of Ho Chi Minh City, the Vietnam Ministry of Health Ethical Review Committee and the Human Research Ethics Committee of the Northern Territory Department of Health and the Menzies School of Health Research. Both Ethics Committees receive annual reports on the trial progress, for continuing approval of the trial.

Protocol amendments

Any modifications to the protocol that may impact on the conduct of the study will be documented in a formal protocol amendment and approved by both Ethics Committees prior to implementation of the changes. The modified protocol will be given a new version number and date. The Ethics Committees will also be notified of any minor corrections/clarifications or administrative changes to the protocol, which will be documented in a protocol amendment letter. Significant protocol changes will also be updated in the ClinicalTrials.gov record.

For peer review only - http://bmjopen.bmj.com/site/about/guidelines.xhtml

Consent

Obtaining consent: The consent process is undertaken by specifically trained study staff. The study staff will go through the PLS and ICF, translated into Vietnamese, in detail with the potential participant's parent/legal guardian. The study staff will then discuss the trial further and answer any questions that may arise. Written informed consent is required prior to enrolment of the infant into the study. Consent is obtained from the parent/legal guardian as the participants are too young to provide consent themselves. A copy of the PLS and ICF will be given to the parent/legal guardian for their records.

Ancillary studies: Specific consent for the indefinite storage of blood and NP samples for future research related to the trial will be obtained from the parent/legal guardian and recorded on the ICF. Any future research will undergo ethical review. Any samples for which indefinite storage is not consented to will be destroyed at the close of the trial.

Confidentiality

All study-related information will be stored securely and held in strict confidence. All documents kept at the study clinics, including the ICFs and participant folders, are stored in locked cabinets. All documents kept centrally are stored in the trial office, which is kept locked. Electronic data is stored in the trial office and on a secure password protected server. The electronic data and laboratory samples are coded by a unique participant number and do not contain the participant name. Access to participants' information will be granted to FHI360 for monitoring purposes, and to the Ethics Committees or DSMB if required.

Access to data

The final trial dataset will be under the custody of the trial sponsor, MCRI. The Principal Investigator, trial manager and trial statistician will have access to the full anonymised final dataset.

Ancillary and post-trial care

Participants are advised to come to the study clinic for ancillary care, or to Children's Hospital Number 2 in Ho Chi Minh City, where they will not be charged for treatment and services. All participants are covered by clinical trials insurance for trial related harms.

For peer review only

Dissemination policy

Plans

Participants will be informed of the overall study results by post, with a postal address collected at the final study visit. Following completion of the trial, the results will be submitted for publication in peer-reviewed journals, and presented at relevant international conferences. Agreements between MCRI and each of the Pasteur Institute of Ho Chi Minh City and GSK Biologicals SA provide that a party must obtain the prior approval of the other parties in advance of submitting a manuscript for publication, and that such approval will not be unreasonably withheld.

Authorship

A publication subcommittee will consider all proposed publications, with the final decision on content and authorship resting with the Principal Investigator. The role of each author will be published. Group authors may be used where appropriate. There are no plans for the use of professional writers.

Reproducible research

There are no plans to grant public access to the full protocol, participant-level dataset or statistical code.

For peer review only

References

1. Myint TT, Madhava H, Balmer P, et al. The impact of 7-valent pneumococcal conjugate vaccine on invasive pneumococcal disease: a literature review. *Advances in therapy* 2013;30:127-51. doi:10.1007/s12325-013-0007-6.
2. Conklin L, Loo JD, Kirk J, et al. Systematic review of the effect of pneumococcal conjugate vaccine dosing schedules on vaccine-type invasive pneumococcal disease among young children. *Pediatr Infect Dis J* 2014;33 Suppl 2:S109-18. doi:10.1097/INF.0000000000000078.
3. Loo JD, Conklin L, Fleming-Dutra KE, et al. Systematic review of the effect of pneumococcal conjugate vaccine dosing schedules on prevention of pneumonia. *Pediatr Infect Dis J* 2014;33 Suppl 2:S140-51. doi:10.1097/INF.0000000000000082.
4. Kalin M. Pneumococcal serotypes and their clinical relevance. *Thorax* 1998;53:159-62.
5. Hausdorff WP, Feikin DR, Klugman KP. Epidemiological differences among pneumococcal serotypes. *The Lancet. Infectious diseases* 2005;5:83-93. doi:10.1016/S1473-3099(05)01280-6.
6. Hausdorff WP, Bryant J, Paradiso PR, et al. Which pneumococcal serogroups cause the most invasive disease: implications for conjugate vaccine formulation and use, part I. *Clin Infect Dis* 2000;30:100-21. doi:10.1086/313608.
7. Hausdorff WP, Bryant J, Kloek C, et al. The contribution of specific pneumococcal serogroups to different disease manifestations: implications for conjugate vaccine formulation and use, part II. *Clin Infect Dis* 2000;30:122-40. doi:10.1086/313609.
8. Weinberger DM, Malley R, Lipsitch M. Serotype replacement in disease after pneumococcal vaccination. *Lancet* 2011;378:1962-73. doi:10.1016/S0140-6736(10)62225-8.
9. Vesikari T, Wysocki J, Chevallier B, et al. Immunogenicity of the 10-valent pneumococcal non-typeable Haemophilus influenzae protein D conjugate vaccine (PHiD-CV) compared to the licensed 7vCRM vaccine. *Pediatr Infect Dis J* 2009;28:S66-76. doi:10.1097/INF.0b013e318199f8ef.
10. Yeh SH, Gurtman A, Hurley DC, et al. Immunogenicity and safety of 13-valent pneumococcal conjugate vaccine in infants and toddlers. *Pediatrics* 2010;126:e493-505. doi:10.1542/peds.2009-3027.
11. Kieninger DM, Kueper K, Steul K, et al. Safety, tolerability, and immunologic noninferiority of a 13-valent pneumococcal conjugate vaccine compared to a 7-valent pneumococcal conjugate vaccine given with routine pediatric vaccinations in Germany. *Vaccine* 2010;28:4192-203. doi:10.1016/j.vaccine.2010.04.008.
12. Whitney CG, Pilishvili T, Farley MM, et al. Effectiveness of seven-valent pneumococcal conjugate vaccine against invasive pneumococcal disease: a matched case-control study. *Lancet* 2006;368:1495-502. doi:10.1016/S0140-6736(06)69637-2.
13. Mahon BE, Hsu K, Karumuri S, et al. Effectiveness of abbreviated and delayed 7-valent pneumococcal conjugate vaccine dosing regimens. *Vaccine* 2006;24:2514-20. doi:10.1016/j.vaccine.2005.12.025.
14. Goldblatt D, Southern J, Ashton L, et al. Immunogenicity and boosting after a reduced number of doses of a pneumococcal conjugate vaccine in infants and toddlers. *Pediatr Infect Dis J* 2006;25:312-9. doi:10.1097/01.inf.0000207483.60267.e7.

For peer review only

15. Russell FM, Balloch A, Tang ML, et al. Immunogenicity following one, two, or three doses of the 7-valent pneumococcal conjugate vaccine. *Vaccine* 2009;27:5685-91. doi:10.1016/j.vaccine.2009.06.098.
16. Silfverdal SA, Hogh B, Bergsaker MR, et al. Immunogenicity of a 2-dose priming and booster vaccination with the 10-valent pneumococcal nontypeable Haemophilus influenzae protein D conjugate vaccine. *Pediatr Infect Dis J* 2009;28:e276-82. doi:10.1097/INF.0b013e3181b48ca3.
17. Gutierrez Brito M, Thompson A, Girgenti D, et al. Immunogenicity and safety of 13-valent pneumococcal conjugate vaccine in Mexico. *Revista panamericana de salud publica = Pan American journal of public health* 2013;33:414-21.
18. Rodgers GL, Esposito S, Principi N, et al. Immune response to 13-valent pneumococcal conjugate vaccine with a reduced dosing schedule. *Vaccine* 2013;31:4765-74. doi:10.1016/j.vaccine.2013.08.009.
19. Russell FM, Licciardi PV, Balloch A, et al. Safety and immunogenicity of the 23-valent pneumococcal polysaccharide vaccine at 12 months of age, following one, two, or three doses of the 7-valent pneumococcal conjugate vaccine in infancy. *Vaccine* 2010;28:3086-94. doi:10.1016/j.vaccine.2010.02.065.
20. Huebner RE, Mbelle N, Forrest B, et al. Immunogenicity after one, two or three doses and impact on the antibody response to coadministered antigens of a nonavalent pneumococcal conjugate vaccine in infants of Soweto, South Africa. *Pediatr Infect Dis J* 2002;21:1004-7. doi:10.1097/01.inf.0000036012.37665.fe.
21. Barzilay EJ, O'Brien KL, Kwok YS, et al. Could a single dose of pneumococcal conjugate vaccine in children be effective? Modeling the optimal age of vaccination. *Vaccine* 2006;24:904-13. doi:10.1016/j.vaccine.2005.08.092.
22. Goldblatt D, Southern J, Andrews NJ, et al. Pneumococcal conjugate vaccine 13 delivered as one primary and one booster dose (1 + 1) compared with two primary doses and a booster (2 + 1) in UK infants: a multicentre, parallel group randomised controlled trial. *The Lancet. Infectious diseases* 2017. doi:10.1016/S1473-3099(17)30654-0.
23. O'Brien KL, Dagan R. The potential indirect effect of conjugate pneumococcal vaccines. *Vaccine* 2003;21:1815-25. doi:S0264410X02008071 [pii].
24. Kayhty H, Auranen K, Nohynek H, et al. Nasopharyngeal colonization: a target for pneumococcal vaccination. *Expert Rev Vaccines* 2006;5:651-67. doi:10.1586/14760584.5.5.651.
25. Moore MR, Hyde TB, Hennessy TW, et al. Impact of a conjugate vaccine on community-wide carriage of nonsusceptible Streptococcus pneumoniae in Alaska. *J Infect Dis* 2004;190:2031-8. doi:10.1086/425422.
26. Huang SS, Platt R, Rifas-Shiman SL, et al. Post-PCV7 changes in colonizing pneumococcal serotypes in 16 Massachusetts communities, 2001 and 2004. *Pediatrics* 2005;116:e408-13. doi:10.1542/peds.2004-2338.
27. Dunais B, Bruno P, Carsenti-Dellamonica H, et al. Trends in nasopharyngeal carriage of Streptococcus pneumoniae among children attending daycare centers in southeastern France from 1999 to 2006. *Pediatr Infect Dis J* 2008;27:1033-5. doi:10.1097/INF.0b013e31817bb8cf.
28. van Gils EJ, Veenhoven RH, Hak E, et al. Effect of reduced-dose schedules with 7-valent pneumococcal conjugate vaccine on nasopharyngeal pneumococcal carriage in children: a randomized controlled trial. *JAMA* 2009;302:159-67. doi:10.1001/jama.2009.975.
29. Clarke C, Bakaletz LO, Ruiz-Guinazu J, et al. Impact of protein D-containing pneumococcal conjugate vaccines on non-typeable Haemophilus influenzae acute otitis media and carriage. *Expert Rev Vaccines* 2017;16:1-14. doi:10.1080/14760584.2017.1333905.
30. Balloch A, Licciardi PV, Leach A, et al. Results from an inter-laboratory comparison of pneumococcal serotype-specific IgG measurement and critical

For peer review only - http://bmjopen.bmj.com/site/about/guidelines.xhtml

parameters that affect assay performance. *Vaccine* 2010;28:1333-40. doi:10.1016/j.vaccine.2009.11.011.
31. WHO Expert Committee on Biological Standardization. WHO Technical Report Series 927. Geneva: World Health Organisation, 2005.
32. Feavers I, Knezevic I, Powell M, et al. Challenges in the evaluation and licensing of new pneumococcal vaccines, 7-8 July 2008, Ottawa, Canada. *Vaccine* 2009;27:3681-8. doi:10.1016/j.vaccine.2009.03.087.
33. World Health Organization. WHO/Health Canada Consulation on serological criteria for evaluation and licensing of new Pneumococcal vaccines, Ottawa, Canada, 7-8 July 2008. Secondary World Health Organization. WHO/Health Canada Consulation on serological criteria for evaluation and licensing of new Pneumococcal vaccines, Ottawa, Canada, 7-8 July 2008. http://www.who.int/biologicals/publications/meetings/areas/vaccines/pneumococcal/Pneumo Meeting Report FINAL IK 24_Dec_08.pdf?ua=1.
34. World Health Organization, Expert Committee on Biological Standardization. Recommendations to assure the quality, safety and efficacy of pneumococcal conjugate vaccines. Geneva, 19-23 October 2009. . Secondary World Health Organization, Expert Committee on Biological Standardization. Recommendations to assure the quality, safety and efficacy of pneumococcal conjugate vaccines. Geneva, 19-23 October 2009. . http://www.who.int/biologicals/areas/vaccines/pneumo/Pneumo_final_23APRIL_2010.pdf?ua=1.
35. Licciardi PV, Toh ZQ, Clutterbuck EA, et al. No long-term evidence of hyporesponsiveness after use of pneumococcal conjugate vaccine in children previously immunized with pneumococcal polysaccharide vaccine. *The Journal of allergy and clinical immunology* 2016;137:1772-79 e11. doi:10.1016/j.jaci.2015.12.1303.
36. Richter SS, Heilmann KP, Dohrn CL, et al. Accuracy of phenotypic methods for identification of Streptococcus pneumoniae isolates included in surveillance programs. *J Clin Microbiol* 2008;46:2184-8. doi:10.1128/JCM.00461-08.
37. Satzke C, Turner P, Virolainen-Julkunen A, et al. Standard method for detecting upper respiratory carriage of Streptococcus pneumoniae: updated recommendations from the World Health Organization Pneumococcal Carriage Working Group. *Vaccine* 2013;32:165-79. doi:10.1016/j.vaccine.2013.08.062.
38. Turner P, Hinds J, Turner C, et al. Improved detection of nasopharyngeal cocolonization by multiple pneumococcal serotypes by use of latex agglutination or molecular serotyping by microarray. *J Clin Microbiol* 2011;49:1784-9. doi:10.1128/JCM.00157-11.
39. Smith-Vaughan H, Byun R, Nadkarni M, et al. Measuring nasal bacterial load and its association with otitis media. *BMC Ear Nose Throat Disord* 2006;6:10. doi:10.1186/1472-6815-6-10.
40. Price EP, Harris TM, Spargo J, et al. Simultaneous identification of Haemophilus influenzae and Haemophilus haemolyticus using real-time PCR. *Future microbiology* 2017;12:585-93. doi:10.2217/fmb-2016-0215.
41. Feiveson A. Power by simulation. *The Stata Journal* 2002;2:107-24.
42. Farrington CP, Manning G. Test statistics and sample size formulae for comparative binomial trials with null hypothesis of non-zero risk difference or non-unity relative risk. *Stat Med* 1990;9:1447-54.
43. Dinleyici EC, Yargic ZA. Current knowledge regarding the investigational 13-valent pneumococcal conjugate vaccine. *Expert Rev Vaccines* 2009;8:977-86. doi:10.1586/erv.09.68.
44. Immunogenicity, safety, and tolerability of 13-valent pneumococcal conjugate vaccine given to infants with routine pediatric vaccinations in Mexico: Interim

For peer review only

Report. Abstract: 7th International Symposium on Pneumococci and Pneumococcal Diseases; 2010; Tel Aviv, Israel.
45. Prymula R, Schuerman L. 10-valent pneumococcal nontypeable Haemophilus influenzae PD conjugate vaccine: Synflorix. *Expert Rev Vaccines* 2009;8:1479-500. doi:10.1586/erv.09.113.

AUTHORS' CONTRIBUTIONS

BT was involved with the study design, led the funding and ethics applications, has been involved in the day-to-day management of the trial and data analysis and drafted the protocol and this manuscript. NTT advised on the study design and location, was involved in the approval processes in Vietnam, and has been involved in the day-to-day management and implementation of the trial. DYU advised on the study design and location and has been involved in the day-to-day implementation of the trial. AB advised on the study design, assisted with the funding applications, and advised on and provided oversight of the immunology laboratory procedures. KB advised on the study design and location and has been responsible for the day-to-day management and implementation of the trial. YBC advised on the study design and funding applications, especially the statistical aspects of the trial. PL advised on the study design, assisted with the funding applications, and advised on and provided oversight of the immunology laboratory procedures. CDN advised on the study design and statistical analysis plan. NTMP advised on the study design and location, was involved in the approval processes in Vietnam, and has been involved in the day-to-day management of the trial. CS advised on the study design, assisted with the funding applications, and advised on and provided oversight of the microbiology laboratory procedures. HSV advised on the study design, assisted with the funding applications, and advised on and provided oversight of the microbiology laboratory procedures. TQHV advised on the study design and advised on and provided oversight of the laboratory procedures at Pasteur. TNH advised on the study design and location, undertook consultations, was involved in the approval processes in Vietnam, and has had overall responsibility for the conduct of the trial in Vietnam as Site Principal Investigator. EKM conceived the study, undertook consultations, provided oversight for the funding and ethics applications, provided oversight for the conduct of the trial and data analysis, and has had overall responsibility for all aspects of the trial as the Principal Investigator. All authors contributed to refinement of the study protocol and reviewed and approved this manuscript.

For peer review only

FUNDING STATEMENT

This work is supported by the National Health and Medical Research Council of Australia (grant number 566792) and the Bill & Melinda Gates Foundation (grant number OPP1116833). The doses of PCV10 and funding for the opsonophagocytic assays are provided by GlaxoSmithKline Biologicals SA (GSK).

COMPETING INTERESTS STATEMENT

All authors receive salary support from grants from the National Health and Medical Research Council of Australia and/or the Bill and Melinda Gates Foundation. Non-financial support (in the form of PCV10 vaccine doses) and funding for opsonphagocytic assays are provided by GSK Biologicals SA. EKM is a member of the DSMB for a current Novavax trial, for which he receives consulting fees. He has received travel costs from the GSK group of companies for one international conference, and an honorarium from Merck for one advisory group meeting. He does not have any paid consultancies with or receive any research funds from pharmaceutical companies. Members of CS's team have received awards that were funded (but not assessed) by Pfizer. None of the authors have any other competing interests to declare.

APPENDICES

Appendix 1 - Biological Specimens

Appendix 2 - Plain Language Statement and Informed Consent Form. These materials were translated into Vietnamese, and back-translated into English, by FHI360.

For peer review only

APPENDIX 1

Biological Specimens

Specimens include NP swabs, bacterial isolates cultured from NP swabs, serum from whole blood, plasma from whole blood and peripheral blood mononuclear cells (PBMCs). Long-term storage of specimens is at the Pneumococcal Laboratory at MCRI or at the Pasteur Institute of Ho Chi Minh City at -80°C. No genetic or HIV testing will be performed on stored samples and they will not be used to establish a tissue bank. Consent for the long-term storage of samples and their use in potential future studies is recorded on the ICF.

Sample Collection

Blood samples are collected using a butterfly needle into gel vacutainer tubes or sodium heparin vacutainer tubes. The volume of blood collected at different ages is as follows: 2.0ml at 2 months of age; 3.5ml from 3-10 months and 19 months of age; and 3.5ml or 7.5ml at 18 months and 24 months of age, depending on the assays to be conducted. Blood samples collected into gel vacutainer tubes are kept chilled in a cooler box and transported to the Pasteur Institute laboratory the same day. On arrival at the laboratory the samples are centrifuged and the sera divided into up to three aliquots, stored in micro-tubes and frozen at -80°C prior to analysis. For blood samples where plasma cell and memory B cell responses are assessed, samples are collected into sodium heparin vacutainer tubes and transported to the Pasteur Institute laboratory at room temperature the same day. On arrival at the laboratory plasma and PBMCs are separated from each heparinized blood sample by density gradient centrifugation. Plasma are divided into up to four aliquots and stored at -80°C prior to analysis.

NP samples are collected and transported according to standard guidelines.[1] In brief, NP samples are collected using sterile swabs and placed immediately into 1000µL Skim Milk Tryptone Glucose Glycerol (STGG) transport medium. The samples are kept chilled in a cooler box and transported to the Pasteur Institute laboratory the same day. On arrival at the laboratory two aliquots are removed and the aliquots and original sample are frozen at -80°C prior to analysis.

For peer review only

Serotype-specific IgG

Serotype-specific anti-pneumococcal IgG levels to each of the 13 serotypes in 13v-PCV are measured using a modified 3rd generation standardized ELISA at the Pasteur Institute laboratory.[2] Briefly, microtiter wells are coated with 2.5-10 mg/mL pneumococcal polysaccharide, depending on the serotype. This is diluted in phosphate buffered saline by incubating at 22° C overnight. To neutralize unspecified cell wall polysaccharide antibodies, 1/100 diluted serum samples are incubated overnight with 10 mg/mL of cell wall polysaccharide and 30mg/mL of serotype 22F, before further dilutions. A reference serum (89-SF, Food and Drug Administration, Bethesda MD) is used and incubated overnight with 10 mg/mL of cell wall polysaccharide. Horse radish peroxidase conjugated anti-human IgG and the TMB Peroxidase Substrate system is used for detection. Results are expressed as µg/mL of serotype-specific IgG. Three control sera will be used on each plate to assess inter-assay variation.

Opsonophagocytic Assay (OPA)

OPAs are conducted at the Pneumococcal Laboratory at MCRI.[3] Serial dilutions of a heat-inactivated sera, in Hanks balanced salt solution with Mg^{++}, Ca^{++} and gelatine, are made in a 96-well sterile microtitre plate. Frozen stock of pneumococci are thawed, washed and diluted to 5×10^4 CFU/serotype/mL. Standard bacterial dilutions are added to all wells and the plate incubated at RT for 30 min. At 30 min, baby rabbit complement, thawed just prior to use, followed by HL-60 cells (2×10^7 cells/ml) is added to all test wells. A bacterial control (heat inactivated foetal calf serum in place of human sera and no complement) and complement control (no sera) are included on all plates. Plates are placed on a horizontal shaker and incubated for 45 min at 37°C in 5% CO_2. The reaction is stopped at 45 min by placing the plate on ice. A 10µL aliquot of this mixture is then spotted onto Todd-Hewitt broth–yeast extract (0.5%) agar plates. After application of an overlay THYE agar containing selective antibiotic (Optochin, Spectinomycin, Streptomycin or Trimethoprim) and 2,3,5-Triphenyltetrazolium chloride (TTC), the plates are incubated overnight at 37°C in 5% CO_2. After overnight incubation, plates are counted and the results expressed as opsonisation indices (OI) where the OI is defined as the interpolated dilution of serum that kills 50% of bacteria.

For peer review only

Memory B cells

Analysis of the memory B cell response is undertaken at the Pasteur Institute laboratory, by ELISPOT assay.[3] PBMCs are re-suspended in RPMI Foetal Calf Serum (FCS) at a concentration of $2x10^6$ cells/mL and 100µL added to each well of the culture plate containing an antigen cocktail (Staphylococcus aureus Cowan strain – Pansorbin cells (SAC; 1:5000), 2.5µg/mL CpG and 83ng/mL pokeweed mitogen). Plates are incubated at 37°C with 5% CO_2 and 95% humidity for 5 days. At day 5, cells are harvested and washed and the cell pellet re-suspended in 1mL RPMI-FCS and counted by trypan blue. Cells are then made up to a final concentration of $2x10^6$ cells/mL for seeding onto antigen-coated ELISPOT plates. Multiscreen hydrophobic polyvinyldene difluoride (PVDF) membrane ELISPOT plates coated with anti-IgG (10µg/mL), tetanus toxoid (5µg/mL), diphtheria toxoid (10µg/mL) or pneumococcal polysaccharides conjugated to methylated human serum albumin at concentrations in the range 10-20µg/mL are sealed and incubated overnight at 4°C. ELISPOT plates are then washed and blocked with RPMI-FCS for 30 minutes at 37°C with 5% CO2 and 95% humidity. Cultured cells or *ex vivo* PBMCs are washed and seeded at 200 to $2x10^5$ cells/well of the antigen-coated ELISPOT plates in RPMI-FCS and incubated overnight at 37°C with 5% CO_2 and 95% humidity. Cells are then washed with PBS-T and bound IgG detected with an alkaline phosphatase-conjugated IgG for 4 hours at RT. ELISPOT plates are washed again before addition of an alkaline phosphatase substrate solution (nitroblue tetrazolium plus 5-bromo-4-chloro-3-indoylphosphate in dimethyl formamide). The reaction is stopped with two washes in distilled water. Cells are visualized and counted using an automated ELISPOT reader and software. The total frequency of IgG-secreting antibody-forming cells (AFCs) is used as the positive control and 1,000 IgG AFCs/10^6 cultured PBMCs is the lower cut-off for inclusion in the analysis. Up to $15x10^6$ cells/mL are used for the memory B cell assay at the Pasteur Institute and the remainder of the PBMCs are cryopreserved in liquid nitrogen in aliquots of $8-10x10^6$ cells/mL for planned T cell assays.

S. pneumoniae identification and serotyping

Identification of *S. pneumoniae* is conducted in line with WHO guidelines.[1] In brief, 50µl swab is plated onto Columbia colistin-nalidixic acid blood agar plates, and identification is primarily based on colonial morphology (flat, with a dimple, 1-3mm in size), α-haemolysis and optochin sensitivity. One colony, plus any additional colonies if morphologically distinct, is sub-cultured onto horse blood agar with an optochin disc. Any colonies that are optochin resistant or intermediately resistant but

For peer review only - http://bmjopen.bmj.com/site/about/guidelines.xhtml

otherwise appear to be *S. pneumoniae* are subject to *lytA* PCR,[1] following DNA preparation using the InstaGene matrix (BioRad). All presumptive pneumococci are serotyped, primarily by latex agglutination using reagents produced in-house using antisera from the Statens SerumInstitut, as previously described.[4 5] In summary, pneumococcal culture is made to a 4-5 McFarland density standard and then 10μL of the suspension mixed with 10μL of latex reagent on clear glass slides and rotated for 1 minute. A positive test is indicated by aggregation of latex particles and clearing of the suspension. Isolates that do not react with antisera are subject to *lytA* PCR.

H. influenzae identification

Identification of *H. influenzae* is made from 50μl swab plated onto bacitracin-vancomycin-clindamycin-chocolate-agar. One presumptive *H. influenzae* colony, plus any additional colonies if morphologically distinct, is selected. Colonies are identified as grayish, semi-opaque, smooth, flat or convex, 1-3mm in size. Confirmation is initially demonstrated by X and V growth factor dependence. Capsular and NTHi strains are discriminated using the Phadebact® Haemophilus coagglutination test. All NTHi isolates are tested for beta-lactamase production using nitrocefin.[6] Following identification of presumptive NTHi, DNA is extracted using the InstaGene matrix (BioRad)[7] and tested by *siaT* and *hypD* PCR for discrimination between NTHi and *H. haemolyticus*.[8]

Quantification of *H. influenzae* and pneumococcus

DNA is extracted from 100μl of STGG medium using high-throughput systems (MagNA Pure LC, Roche) using the DNA Isolation Kit II (Bacteria, Fungi) (Roche) incorporating enzymatic digestion. Quantification of *H. influenzae* and pneumococci is then performed using real-time quantitative PCR (qPCR).[9] qPCR targeting the *hpd3* and/or *siaT* gene (*H. influenzae*) or *lytA* gene (pneumococcus) is conducted in 25μl reactions containing 2μl of template DNA on a Stratagene Mx3005 machine using Brilliant III Ultra-Fast qPCR Master Mix (Agilent Technologies) according to the manufacturer's instructions. The density of each bacterial species is assessed in comparison to a set of approximately five reference standards run with each assay to give the density of carriage.

Microarray serotyping

Samples that contain pneumococci are tested by DNA microarray as described previously with minor modifications.[4] Following a culture amplification step (on selective agar such as horse blood agar with 5 μg/ml gentamicin), DNA is extracted

For peer review only - http://bmjopen.bmj.com/site/about/guidelines.xhtml

using the Qiacube HT platform (Qiagen). When only a single α-haemolytic colony grows, it is sub-cultured before DNA extraction for microarray. DNA is labelled and then hybridised to the Senti-SP microarray (formally BUGS microarray), scanned on an Agilent scanner, and uploaded to Senti-Net (a cloud based software platform). Serotype-specific density is calculated by multiplying pneumococcal density (measured by *lytA* qPCR) by the relative abundance of each serotype (determined by microarray).

Immunogenicity of *Infanrix-hexa*

The specific IgG to *Haemophilus influenzae* type b (Hib) will be measured by ELISA. High binding ELISA plates are coated with Hib polysaccharide (HBO-HA, the PRP capsular linked to human albumin) antigen and incubated at 37°C for 2 hours and then overnight at 4°C. The plates are washed and blocked with 1% Gelatin in PBS, then loaded with dilutions of standards and patient samples. Following two hours incubation at 37°C, the plates are washed and peroxidase-labelled anti-human IgG is added to each well. Bound specific antibody is detected using the substrate TMB. After the substrate reaction, the intensity of the colour developed is proportional to the amount of IgG-specific antibodies detected in the sample. Results for the samples are determined directly using a standard curve and expressed as µg/mL. Three control sera will be used on each plate to assess inter-assay variation.

The specific IgG to tetanus and diphtheria will be measured using a commercial solid phase ELISA (Genzyme Virotech). The wells are coated with antigen. Specific antibodies of the sample bind to the antigen coated wells and are detected by a secondary enzyme conjugated antibody specific for human IgG. After the substrate reaction, the intensity of the colour developed is proportional to the amount of IgG-specific antibodies detected in the sample. Results for the samples are determined directly using a standard curve and expressed as IU/mL. Two control sera will be used on each plate to assess inter-assay variation.

The Hepatitis B surface antibodies will be measured using AxSym analyzer system. Patient serum is incubated with Micro-particles coated with recombinant HbsAg. Antibody present in the serum binds with antigen on the particles. When this reaction mixture is transferred to the matrix cell, the micro-particles bind irreversibly to the glass fibre matrix. Biotinylated rHBsAg is then added forming an antigen-antibody-antigen complex. Anti-Biotin: Alkaline phosphatase conjugate is dispensed onto the matrix cell and binds with any microparticle-bound antigen-antibody-antigen complex.

For peer review only - http://bmjopen.bmj.com/site/about/guidelines.xhtml

The matrix cell is washed to remove any unbound antibody and the substrate 4-Methylumbelliferyl Phosphate is added. The alkaline phosphatase-labelled conjugate catalyses the removal of a phosphate group from the substrate, yielding a fluorescent product, 4-Methylumbelliferone. This fluorescent product is measured and the concentration of anti-HBs in the sample is determined from a calibration curve and will be reported in IU/mL. A positive and negative control will be included in each assay.

The specific IgG to *B. pertussis* (PT) will be measured using a commercial solid phase ELISA (Genzyme Virotech). The wells are coated with antigen. Specific antibodies of the sample bind to the antigen coated wells and are detected by a secondary enzyme conjugated antibody specific for human IgG. After the substrate reaction, the intensity of the colour developed is proportional to the amount of IgG-specific antibodies detected in the sample. Results for the samples are derived using the optical density ratio of the cut-off control and the patient sample and expressed in VE or Virotech Units which have been calibrated with the reference standard IgG anti-Pertussis toxin (Lot 3, 200 U/ml) of the Centre for Biologic Evaluation and Research (CBER), FDA. Three control sera will be used on each plate to assess inter-assay variation.

For peer review only

Appendix 1 - References

1. Satzke C, Turner P, Virolainen-Julkunen A, et al. Standard method for detecting upper respiratory carriage of Streptococcus pneumoniae: updated recommendations from the World Health Organization Pneumococcal Carriage Working Group. *Vaccine* 2013;32:165-79. doi:10.1016/j.vaccine.2013.08.062.
2. Balloch A, Licciardi PV, Leach A, et al. Results from an inter-laboratory comparison of pneumococcal serotype-specific IgG measurement and critical parameters that affect assay performance. *Vaccine* 2010;28:1333-40. doi:10.1016/j.vaccine.2009.11.011.
3. Licciardi PV, Toh ZQ, Clutterbuck EA, et al. No long-term evidence of hyporesponsiveness after use of pneumococcal conjugate vaccine in children previously immunized with pneumococcal polysaccharide vaccine. *The Journal of allergy and clinical immunology* 2016;137:1772-79 e11. doi:10.1016/j.jaci.2015.12.1303.
4. Satzke C, Dunne EM, Porter BD, et al. The PneuCarriage Project: A Multi-Centre Comparative Study to Identify the Best Serotyping Methods for Examining Pneumococcal Carriage in Vaccine Evaluation Studies. *PLoS medicine* 2015;12:e1001903; discussion e03. doi:10.1371/journal.pmed.1001903.
5. Porter BD, Ortika BD, Satzke C. Capsular Serotyping of Streptococcus pneumoniae by latex agglutination. *Journal of visualized experiments : JoVE* 2014:51747. doi:10.3791/51747.
6. Tenover FC, Huang MB, Rasheed JK, et al. Development of PCR assays to detect ampicillin resistance genes in cerebrospinal fluid samples containing Haemophilus influenzae. *J Clin Microbiol* 1994;32:2729-37.
7. Carter J, Hutton S, Sriprakash KS, et al. Culture of the causative organism of donovanosis (Calymmatobacterium granulomatis) in HEp-2 cells. *J Clin Microbiol* 1997;35:2915-7.
8. Price EP, Harris TM, Spargo J, et al. Simultaneous identification of Haemophilus influenzae and Haemophilus haemolyticus using real-time PCR. *Future microbiology* 2017;12:585-93. doi:10.2217/fmb-2016-0215.
9. Smith-Vaughan H, Byun R, Nadkarni M, et al. Measuring nasal bacterial load and its association with otitis media. *BMC Ear Nose Throat Disord* 2006;6:10. doi:10.1186/1472-6815-6-10.

For peer review only

APPENDIX 2

Plain Language Statements and Informed Consent Form

These materials were translated into Vietnamese, and back-translated into English, by FHI360. This trial uses two Plain Language Statements, one for participants enrolled at 2 months of age and randomised into Arms A-F, and one for participants enrolled at 18 months of age into Arm G. The same Informed Consent Form is used for participants in all Arms.

For peer review only

PO Box 41096, Casuarina NT 0811, Australia
John Mathews Building (Bldg 58),
Royal Darwin Hospital Campus, Rocklands Dve, Casuarina NT 0810
Ph: 08 8922 8196 | Fax: 08 8927 5187 | Web: www.menzies.edu.au
ABN: 70 413 542 847

discovery for a healthy tomorrow

INFORMATION SHEET: Vietnam Pneumococcal Vaccine Study

This is for you to keep.

Principal Investigators:
Assoc. Prof. Tran Ngoc Huu
Prof. Edward Kim Mulholland

Research Partners:
Pasteur Institute, Ho Chi Minh City
Menzies School of Health Research
Murdoch Childrens Research Institute

Introduction

Health research helps us to understand diseases and find ways to prevent them. Vaccines (like the routine baby injections) are an important way to prevent diseases. Pneumonia is a common problem in Vietnam and throughout the developing world. In the developing world it is the leading cause of death amongst under 5 year olds. A number of germs cause pneumonia but the most common germ is a bacteria called pneumococcus. Pneumococcus can also cause ear infections as well as other, more severe diseases like meningitis (infection around the brain). This germ normally lives in the nose of humans and is spread from person to person by touching or sneezing. There are more than 90 types of this germ but only some types cause serious infections in young children.

Why are we doing the study?

There are vaccines available to protect against infection with pneumococcus. These are called pneumococcal vaccines. Many countries around the world give all their babies a pneumococcal vaccine that protects against 7 types of the pneumococcal disease (7v-PCV). There are two new vaccines which have been developed. Both new vaccines give more protection against pneumococcal disease than the 7v-PCV. Both vaccines have completed all their tests and are licensed and being used by many countries in Europe and the United States. The clinical trials have shown that these vaccines are safe; therefore there is little danger to any child participating in this study. The vaccines are likely to provide some protection from ear infections and pneumonia. Unfortunately the costs of these vaccines are very high, so not all countries in the world can afford them. We are doing this study to find the best ways to protect babies from this germ and also to make it cheaper for countries, like Vietnam, to afford to buy the vaccine.

Benefits of the study

By joining the study your baby can be protected from the commonest pneumococcal germs. Both these vaccines are very expensive and are not presently available to other babies in Vietnam. They have been especially made for use in babies and young children and will protect the babies from the common diseases caused by the pneumococcus. We hope to find a schedule that works and which countries like Vietnam can afford. In addition children will receive 4 doses of *Infanrix-Hexa*: 3 doses during early infancy and a booster dose at either 18 or 19 months of age.

For peer review only - http://bmjopen.bmj.com/site/about/guidelines.xhtml

What does the study involve?

The study will include 1400 babies and we will be looking at 7 different vaccine schedules in this study. 1200 babies will be enrolled at 2 months old and will be randomly allocated to 1 of 6 groups. An additional 200 babies will be enrolled at 18 months old to act as controls.

Consent: A study doctor or nurse will discuss the study with each child's parent or legal guardian. They will explain what is involved and ask some questions about the baby's health. If you agree to join the study she will ask you to sign a consent form which says that you agree for your baby to join. If you consent to taking part in the study, she will perform a health check of your baby to make sure your baby is healthy to take part.

Vaccinations & health checks: If you agree to your baby to take part in the study you will need to come to the clinic between 9 and 11 times over a period of 22 months. The study nurse will remind you when you need to come. Like rolling a dice your baby will be allocated to 1 of 6 groups. Your baby will get between one and four doses of one of the two types of Pneumococcal vaccine, either the Prevnar-13 (13v-PCV which covers 13 types of the pneumococcal germ) or the 10v-Synflorix vaccine (which covers 10 types of the pneumococcal germ and may be better at protecting against pneumonia). Depending on which group your baby is randomly placed in will depend on when, how many doses and what type of Pneumococcal vaccine your baby will receive. Your baby will also get an infant vaccine (Infanrix-hexa 6-1) that covers all the diseases (diphtheria, tetanus, pertussis, hepatitis B, polio virus and *Haemophilus influenzae* type B) that are covered by the standard vaccines used in VIetnam. Vaccines will be given by staff from Pasteur Institute Ho Chi Minh City. Your baby will also have regular health checks during the study.

Questionnaire: At the start of the study you will be asked some general questions about your family and your baby's health. These are simply to help us understand how the vaccines work best. The results will be kept confidential (see below).

Blood tests: Up to four blood tests will be taken during the study, by staff from Children's Hospital Number 2. The blood tests are to check the response to the vaccines. If you would prefer, we can put local anesthetic cream on your baby's skin before taking the blood test so that it doesn't hurt as much. The amount of blood taken will vary depending on the age of the child: 2.0mls at 2 months of age; 3.5mls from 3 to 10 and 19 months of age; and 3.5mls or 7.5mls at 18 and 24 months of age.

Nose swabs: Six nose swabs will be taken during the study, at 2, 6, 9, 12, 18 and 24 months of age. The nose swabs are to see if the vaccine will help stop the spread of the pneumococcus from child to child. This will involve putting a cotton wool swab (like a cotton bud) into the baby's nose for a couple of seconds. This may make the baby sneeze and possibly cry briefly – it tickles quite a lot, but doesn't really hurt.

Summary of changes: Additional procedures and vaccines

Groups A-E	*18 months*	*Measles and Rubella given*
	19 months	*Infanrix Hexa given*
	24 months	*Nose swab taken*
Group F	*18 months*	*Infanrix Hexa given*
	19 months	*Measles and Rubella given*<br>*Blood taken*
	24 months	*Nose swab taken*<br>*Blood taken*<br>*Synflorix given*

Hospital record review: If your baby becomes unwell during the study, the staff may need to look at your child's medical records.

Menzies School of Health Research
PO Box 41096, Casuarina NT 0811, Australia | John Mathews Building (Bldg 58), Royal Darwin Hospital Campus, Rocklands Dve, Casuarina NT 0810
[illegible]

For peer review only - http://bmjopen.bmj.com/site/about/guidelines.xhtml

For peer review only

Are there any risks?

The vaccines we are using are licensed many countries. As with all vaccines there is likely to be some pain felt, and there is a small risk of soreness and redness where the vaccine was given. Babies in the study will get up to 4 extra injections than they would routinely get. We will check the babies to make sure they don't have any unexpected reactions. We also have a study doctor who will be keeping a record of any serious illnesses that are unlikely to occur during the study.

Confidentiality

All information collected in this study will remain confidential and will be used for research purposes only. All information will be kept secure. Your baby will be given an identification number at the start of the study. Any information collected will use this number and will not include your baby's name. The samples we collect will be sent to overseas laboratories to have further tests. These laboratories will not be given your child's name. We will ask your permission if it is alright for your baby's blood and nose swab samples to be stored indefinitely for other similar tests in the future. This would help us to perform any new pneumococcal test that may be developed in the future. The results of the study will be published in scientific journals and presented at conferences. There will never be details published that would identify your baby.

Voluntary Participation and Withdrawal from the Study

Your baby does not have to take part in the study. Your baby will get the best treatment available and the full attention of the health staff even if they do not participate. You are free to withdraw your baby from the study at any point. This will not affect any of your baby's further health care treatment and there will be no harmful consequences for your baby. If your baby has not had all their pneumococcal vaccines they may not be fully protected against the pneumococcal germs which most commonly affect infants. However, they will still gain some protection from the doses of vaccine received.

Compensation

We will pay 200,000VND towards the transport cost for coming to the clinic for each study visit. If your baby becomes ill or injured as a result of taking part in this clinical study, medical treatment will be provided.

Ethical Approval

This study has been approved by the People's Committee of Ho Chi Minh City. This study has also been approved by the Vietnam Ministry of Health Ethics Committee and the Menzies School of Health Research Ethics Committee, Australia. The ethics committees make sure that the study is being done in the best and safest way. If you have any concerns or complaints regarding the conduct of the research project you are invited to contact:

Vietnam Ministry of Health
Ethics Committee
Phone: 04 62732156

OR

Human Research Ethics Committee of the NT
Department of Health and Menzies School of Health Research
PO Box 41096, Casuarina, NT 0811, Australia
Phone: 61 8 8922 7922
Email: ethics@menzies.edu.au

How is the study funded?

The funding to perform the study is from the National Health and Medical Research Council, Australia and the Bill & Melinda Gates Foundation.

For peer review only - http://bmjopen.bmj.com/site/about/guidelines.xhtml

Your Right to Ask Questions

Please feel free to contact us if you have any questions or concerns.

If you have any questions regarding the study activities, please phone:

If you have any questions regarding adverse events, please phone:

Commune Health Centre Number:

For peer review only

Menzies School of Health Research
PO Box 41096, Casuarina NT 0811, Australia | John Mathews Building (Bldg 58), Royal Darwin Hospital Campus, Rocklands Dve, Casuarina NT 0810
Ph: 08 8922 8196 | Fax: [illegible] | Web: www.menzies.edu.au | ABN: [illegible]

For peer review only - http://bmjopen.bmj.com/site/about/guidelines.xhtml

menzies
school of health research

PO Box 41096, Casuarina NT 0811, Australia
John Mathews Building (Bldg 58),
Royal Darwin Hospital Campus, Rocklands Dve, Casuarina NT 0810
Ph: 08 8922 8196 | Fax: 08 8927 5187 | Web: www.menzies.edu.au
ABN: 70 413 542 847

discovery for a healthy tomorrow

INFORMATION SHEET: Vietnam Pneumococcal Vaccine Study (Control group)

This is for you to keep.

Principal Investigators:
Assoc. Prof. Tran Ngoc Huu
Prof. Edward Kim Mulholland

Research Partners:
Pasteur Institute, Ho Chi Minh City
Menzies School of Health Research
Murdoch Children's Research Institute

For peer review only

Introduction

Health research helps us to understand diseases and find ways to prevent them. Vaccines (like the routine baby injections) are an important way to prevent diseases. Pneumonia is a common problem in Vietnam and throughout the developing world. In the developing world it is the leading cause of death amongst under 5 year olds. A number of germs cause pneumonia but the most common germ is a bacteria called pneumococcus. Pneumococcus can also cause ear infections as well as other, more severe diseases like meningitis (infection around the brain). This germ normally lives in the nose of humans and is spread from person to person by touching or sneezing. There are more than 90 types of this germ but only some types cause serious infections in young children.

Why are we doing the study?

There are vaccines available to protect against infection with pneumococcus. These are called pneumococcal vaccines. Many countries around the world give all their babies a pneumococcal vaccine that protects against 7 types of the pneumococcal disease (7v-PCV). There are two new vaccines which have been developed. Both new vaccines give more protection against pneumococcal disease than the 7v-PCV.Both vaccines have completed all their tests and are licensed and being used by many countries in Europe and the United States. The clinical trials have shown that these vaccines are safe; therefore there is little danger to any child participating in this study. The vaccines are likely to provide some protection from ear infections and pneumonia. Unfortunately the costs of these vaccines are very high, so not all countries in the world can afford them. We are doing this study to find the best ways to protect babies from this germ and also to make it cheaper for countries, like Vietnam, to afford to buy the vaccine.

Benefits of the study

By joining the study your baby can be protected from the commonest pneumococcal germs. Both these vaccines are very expensive and are not presently available to other babies in Vietnam. They have been especially made for use in babies and young children and will protect the babies from the common diseases caused by the pneumococcus. We hope to find a schedule that works and which countries like Vietnam can afford. In addition your baby will receive a dose of Infanrix-hexa at 18 months of age.

For peer review only - http://bmjopen.bmj.com/site/about/guidelines.xhtml

What does the study involve?

The study will include 200 babies to act as comparisons to participants in an existing study of six different vaccine schedules.

Consent: A study doctor or nurse will discuss the study with each child's parent or legal guardian. They will explain what is involved and ask some questions about the baby's health. If you agree to join the study she will ask you to sign a consent form which says that you agree for your baby to join. If you consent to taking part in the study, she will perform a health check of your baby to make sure your baby is healthy to take part.

Vaccinations & health checks: If you agree to your baby to take part in the study you will need to come to the clinic 3 times over a period of 6 months. The study nurse will remind you when you need to come. Your baby will get a single dose of (Infanrix-hexa 6-1) that covers six diseases (diphtheria, tetanus, pertussis, hepatitis B, polio virus and *Haemophilus influenzae* type B) at 18 months of age, a single dose of Measles and Rubella (MR) at 19 months of age and a single dose of Pneumococcal vaccine (10v-Synflorix vaccine, which covers 10 types of the pneumococcal germ) at 24 months of age. Vaccines will be given by staff from Pasteur Institute Ho Chi Minh City. Your baby will also have a doctor's health check at each study visit.

Questionnaire: At the start of the study you will be asked some general questions about your family and your baby's health. These are simply to help us understand how the vaccines work best. The results will be kept confidential (see below).

Blood tests: Three blood tests will be taken over the six months, by staff from Children's Hospital Number 2. The blood tests are to check the response to the vaccines. If you would prefer, we can put local anesthetic cream on your baby's skin before taking the blood test so that it doesn't hurt as much. The amount of blood taken will be 3.5 or 7.5mls at 18 and 24 months of age; and 3.5mls at 19 months of age.

Nose swabs: Two nose swabs will be taken during the study, at 18 and 24 months of age. The nose swabs are to see if the vaccine will help stop the spread of the pneumococcus from child to child. This will involve putting a cotton wool swab (like a cotton bud) into the baby's nose for a couple of seconds. This may make the baby sneeze and possibly cry briefly – it tickles quite a lot, but doesn't really hurt.

Hospital record review: If your baby becomes unwell during the study, the staff may need to look at your child's medical records.

Are there any risks?

The vaccines we are using are licensed many countries. As with all vaccines there is likely to be some pain felt, and there is a small risk of soreness and redness where the vaccine was given. We will check the babies to make sure they don't have any unexpected reactions. We also have a study doctor who will be keeping a record of any serious illnesses that are unlikely to occur during the study.

Confidentiality

All information collected in this study will remain confidential and will be used for research purposes only. All information will be kept secure. Your baby will be given an identification number at the start of the study. Any information collected will use this number and will not include your baby's name. The samples we collect will be sent to overseas laboratories to have further tests. These laboratories will not be given your child's name. We will ask your permission if it is alright for your baby's blood and nose swab samples to be stored indefinitely for other similar tests in the future. This would help us to perform any new pneumococcal test that may be developed in the

For peer review only - http://bmjopen.bmj.com/site/about/guidelines.xhtml

future. The results of the study will be published in scientific journals and presented at conferences. There will never be details published that would identify your baby.

Voluntary Participation and Withdrawal from the Study

Your baby does not have to take part in the study. Your baby will get the best treatment available and the full attention of the health staff even if they do not participate. You are free to withdraw your baby from the study at any point. This will not affect any of your baby's further health care treatment and there will be no harmful consequences for your baby. If your baby has not had all their pneumococcal vaccines they may not be fully protected against the pneumococcal germs which most commonly affect infants. However, they will still gain some protection from the doses of vaccine received.

Compensation

We will pay 200,000VND towards the transport cost for coming to the clinic for each study visit. If your baby becomes ill or injured as a result of taking part in this clinical study, medical treatment will be provided.

Ethical Approval

This study has been approved by the People's Committee of Ho Chi Minh City. This study has also been approved by the Vietnam Ministry of Health Ethics Committee and the Menzies School of Health Research Ethics Committee, Australia. The ethics committees make sure that the study is being done in the best and safest way. If you have any concerns or complaints regarding the conduct of the research project you are invited to contact:

Vietnam Ministry of Health<br>Ethics Committee<br>Phone: 04 62732156	OR	Human Research Ethics Committee of the NT<br>Department of Health and Menzies School of Health Research<br>PO Box 41096, Casuarina, NT 0811, Australia<br>Phone: 61 8 8922 7922<br>Email: ethics@menzies.edu.au

How is the study funded?

The funding to perform the study is from the National Health and Medical Research Council, Australia and the Bill & Melinda Gates Foundation.

Your Right to Ask Questions

Please feel free to contact us if you have any questions or concerns.

If you have any questions regarding the study activities, please phone:

If you have any questions regarding adverse events, please phone:

Commune Health Centre Number:

For peer review only - http://bmjopen.bmj.com/site/about/guidelines.xhtml

menzies school of health research

PO Box 41096, Casuarina NT 0811, Australia
John Mathews Building (Bldg 58),
Royal Darwin Hospital Campus, Rocklands Dve, Casuarina NT 0810
Ph: 08 8922 8196 | Fax: 08 8927 5187 | Web: www.menzies.edu.au
ABN: 70 413 542 847

discovery for a healthy tomorrow

CONSENT FORM

This means you can say NO

**Screening Number:**	|___|___|___|___|
**Participant ID:**	|___|___|___|___|
**Date:**	__ __ /__ __ /__ __ dd / mm / yy

Principal Investigators:

Assoc. Prof. Tran Ngoc Huu
Prof. Edward Kim Mulholland

Research Partners:

Pasteur Institute, Ho Chi Minh City
Menzies School of Health Research

This form is to record if you agree for your infant to take part in the **"Evaluation of Different Infant Vaccination Schedules Incorporating Pneumococcal Vaccination"**. You should only sign this form if you are happy that the information about the study has been clearly explained to you, you have received enough information about the study and you have had all your questions answered satisfactorily.

Please record the name of the person you have spoken to about the study:

____

By agreeing for your infant to take part in the study, you understand that:

- You are free to withdraw your child from the study at any time without having to give a reason;
- Your child will be vaccinated against all the diseases that are covered by the standard vaccines used in Vietnam, although these vaccines may be given at different times;
- If your child becomes sick, their hospital records will be reviewed by the study doctor or other designated study staff; and
- The samples taken in this study will be sent to overseas laboratories to test vaccine responses and carriage of bacteria

For peer review only

For peer review only - http://bmjopen.bmj.com/site/about/guidelines.xhtml

menzies
school of health research

PO Box 41096, Casuarina NT 0811, Australia
John Mathews Building (Bldg 58),
Royal Darwin Hospital Campus, Rocklands Dve, Casuarina NT 0810
Ph: 08 8922 8196 | Fax: 08 8927 5187 | Web: www.menzies.edu.au
ABN: 70 413 542 847

discovery for a healthy tomorrow

Screening Number: |___|___|___|___|

Participant ID: |___|___|___|___|

Date: __ __ /__ __ /__ __

Consent:

❑ YES, I agree for my infant to take part in this study.

❑ NO, I do not agree for my infant to take part in this study.

Use of samples:

❑ YES, you may indefinitely store my unused identified samples for future work in the same general area of research that has obtained ethics committee approval.

❑ NO, you may **NOT USE** my samples for future research. Destroy my unused samples at the close of the study.

Signed (parent/legal guardian): ______________________ Date: __ __ /__ __ /__ __
dd / mm / yy

Name of parent/legal guardian: ______________________ Time: __ __ :__ __
hh : mm

Relationship to infant: ______________________

Name of infant or baby of: ______________________

Infant Sex: male / female Infant DOB: __ __ /__ __ /__ __
dd / mm / yy

Signed (study nurse): ______________________ Date: __ __ /__ __ /__ __
dd / mm / yy

***If illiterate:** A literate witness must sign (if possible, this person should be selected by the participant and should have no connection to the research team).*

I have witnessed the accurate reading of the consent form to the parent of the potential participant, and the individual has had the opportunity to ask questions. I confirm that the individual has given consent freely.

Signed (witness): ______________________ Date: __ __ /__ __ /__ __
dd / mm / yy

Name of witness: ______________________

For peer review only

For peer review only - http://bmjopen.bmj.com/site/about/guidelines.xhtml

SPIRIT
STANDARD PROTOCOL ITEMS: RECOMMENDATIONS FOR INTERVENTIONAL TRIALS

SPIRIT 2013 Checklist: Recommended items to address in a clinical trial protocol and related documents*

Section/item	Item No	Description	Addressed on page number
**Administrative information**			
Title	1	Descriptive title identifying the study design, population, interventions, and, if applicable, trial acronym	______3_______
Trial registration	2a	Trial identifier and registry name. If not yet registered, name of intended registry	______3_______
	2b	All items from the World Health Organization Trial Registration Data Set	______3_______
Protocol version	3	Date and version identifier	______5_______
Funding	4	Sources and types of financial, material, and other support	______30______
Roles and responsibilities	5a	Names, affiliations, and roles of protocol contributors	______29______
	5b	Name and contact information for the trial sponsor	______5_______
	5c	Role of study sponsor and funders, if any, in study design; collection, management, analysis, and interpretation of data; writing of the report; and the decision to submit the report for publication, including whether they will have ultimate authority over any of these activities	______5_______
	5d	Composition, roles, and responsibilities of the coordinating centre, steering committee, endpoint adjudication committee, data management team, and other individuals or groups overseeing the trial, if applicable (see Item 21a for data monitoring committee)	______n/a______

For peer review only

Introduction

Background and rationale	6a	Description of research question and justification for undertaking the trial, including summary of relevant studies (published and unpublished) examining benefits and harms for each intervention	______6_______
	6b	Explanation for choice of comparators	______8_______
Objectives	7	Specific objectives or hypotheses	______9______
Trial design	8	Description of trial design including type of trial (eg, parallel group, crossover, factorial, single group), allocation ratio, and framework (eg, superiority, equivalence, noninferiority, exploratory)	______11______

Methods: Participants, interventions, and outcomes

Study setting	9	Description of study settings (eg, community clinic, academic hospital) and list of countries where data will be collected. Reference to where list of study sites can be obtained	______12______
Eligibility criteria	10	Inclusion and exclusion criteria for participants. If applicable, eligibility criteria for study centres and individuals who will perform the interventions (eg, surgeons, psychotherapists)	______12______
Interventions	11a	Interventions for each group with sufficient detail to allow replication, including how and when they will be administered	______13______
	11b	Criteria for discontinuing or modifying allocated interventions for a given trial participant (eg, drug dose change in response to harms, participant request, or improving/worsening disease)	______14______
	11c	Strategies to improve adherence to intervention protocols, and any procedures for monitoring adherence (eg, drug tablet return, laboratory tests)	______14______
	11d	Relevant concomitant care and interventions that are permitted or prohibited during the trial	______14______
Outcomes	12	Primary, secondary, and other outcomes, including the specific measurement variable (eg, systolic blood pressure), analysis metric (eg, change from baseline, final value, time to event), method of aggregation (eg, median, proportion), and time point for each outcome. Explanation of the clinical relevance of chosen efficacy and harm outcomes is strongly recommended	______15______
Participant timeline	13	Time schedule of enrolment, interventions (including any run-ins and washouts), assessments, and visits for participants. A schematic diagram is highly recommended (see Figure)	______17______

For peer review only - http://bmjopen.bmj.com/site/about/guidelines.xhtml

Sample size	14	Estimated number of participants needed to achieve study objectives and how it was determined, including clinical and statistical assumptions supporting any sample size calculations	______18______
Recruitment	15	Strategies for achieving adequate participant enrolment to reach target sample size	______19______

Methods: Assignment of interventions (for controlled trials)

Allocation:

Sequence generation	16a	Method of generating the allocation sequence (eg, computer-generated random numbers), and list of any factors for stratification. To reduce predictability of a random sequence, details of any planned restriction (eg, blocking) should be provided in a separate document that is unavailable to those who enrol participants or assign interventions	______19______
Allocation concealment mechanism	16b	Mechanism of implementing the allocation sequence (eg, central telephone; sequentially numbered, opaque, sealed envelopes), describing any steps to conceal the sequence until interventions are assigned	______19______
Implementation	16c	Who will generate the allocation sequence, who will enrol participants, and who will assign participants to interventions	______19______
Blinding (masking)	17a	Who will be blinded after assignment to interventions (eg, trial participants, care providers, outcome assessors, data analysts), and how	______19______
	17b	If blinded, circumstances under which unblinding is permissible, and procedure for revealing a participant's allocated intervention during the trial	______n/a______

Methods: Data collection, management, and analysis

Data collection methods	18a	Plans for assessment and collection of outcome, baseline, and other trial data, including any related processes to promote data quality (eg, duplicate measurements, training of assessors) and a description of study instruments (eg, questionnaires, laboratory tests) along with their reliability and validity, if known. Reference to where data collection forms can be found, if not in the protocol	______20______
	18b	Plans to promote participant retention and complete follow-up, including list of any outcome data to be collected for participants who discontinue or deviate from intervention protocols	______20______

For peer review only - http://bmjopen.bmj.com/site/about/guidelines.xhtml

Data management	19	Plans for data entry, coding, security, and storage, including any related processes to promote data quality (eg, double data entry; range checks for data values). Reference to where details of data management procedures can be found, if not in the protocol	______20______
Statistical methods	20a	Statistical methods for analysing primary and secondary outcomes. Reference to where other details of the statistical analysis plan can be found, if not in the protocol	______20______
	20b	Methods for any additional analyses (eg, subgroup and adjusted analyses)	______21______
	20c	Definition of analysis population relating to protocol non-adherence (eg, as randomised analysis), and any statistical methods to handle missing data (eg, multiple imputation)	______22______
**Methods: Monitoring**			
Data monitoring	21a	Composition of data monitoring committee (DMC); summary of its role and reporting structure; statement of whether it is independent from the sponsor and competing interests; and reference to where further details about its charter can be found, if not in the protocol. Alternatively, an explanation of why a DMC is not needed	______22______
	21b	Description of any interim analyses and stopping guidelines, including who will have access to these interim results and make the final decision to terminate the trial	______22______
Harms	22	Plans for collecting, assessing, reporting, and managing solicited and spontaneously reported adverse events and other unintended effects of trial interventions or trial conduct	______23______
Auditing	23	Frequency and procedures for auditing trial conduct, if any, and whether the process will be independent from investigators and the sponsor	______23______
**Ethics and dissemination**			
Research ethics approval	24	Plans for seeking research ethics committee/institutional review board (REC/IRB) approval	______23______
Protocol amendments	25	Plans for communicating important protocol modifications (eg, changes to eligibility criteria, outcomes, analyses) to relevant parties (eg, investigators, REC/IRBs, trial participants, trial registries, journals, regulators)	______23______

For peer review only - http://bmjopen.bmj.com/site/about/guidelines.xhtml

Consent or assent	26a	Who will obtain informed consent or assent from potential trial participants or authorised surrogates, and how (see Item 32)	______24______
	26b	Additional consent provisions for collection and use of participant data and biological specimens in ancillary studies, if applicable	______24______
Confidentiality	27	How personal information about potential and enrolled participants will be collected, shared, and maintained in order to protect confidentiality before, during, and after the trial	______24______
Declaration of interests	28	Financial and other competing interests for principal investigators for the overall trial and each study site	______30______
Access to data	29	Statement of who will have access to the final trial dataset, and disclosure of contractual agreements that limit such access for investigators	______24______
Ancillary and post-trial care	30	Provisions, if any, for ancillary and post-trial care, and for compensation to those who suffer harm from trial participation	______24______
Dissemination policy	31a	Plans for investigators and sponsor to communicate trial results to participants, healthcare professionals, the public, and other relevant groups (eg, via publication, reporting in results databases, or other data sharing arrangements), including any publication restrictions	______25______
	31b	Authorship eligibility guidelines and any intended use of professional writers	______25______
	31c	Plans, if any, for granting public access to the full protocol, participant-level dataset, and statistical code	______25______
**Appendices**			
Informed consent materials	32	Model consent form and other related documentation given to participants and authorised surrogates	_Appendices.pdf_
Biological specimens	33	Plans for collection, laboratory evaluation, and storage of biological specimens for genetic or molecular analysis in the current trial and for future use in ancillary studies, if applicable	_Appendices.pdf_

*It is strongly recommended that this checklist be read in conjunction with the SPIRIT 2013 Explanation & Elaboration for important clarification on the items. Amendments to the protocol should be tracked and dated. The SPIRIT checklist is copyrighted by the SPIRIT Group under the Creative Commons "Attribution-NonCommercial-NoDerivs 3.0 Unported" license.

For peer review only - http://bmjopen.bmj.com/site/about/guidelines.xhtml

BMJ Open

Evaluation of different infant vaccination schedules incorporating pneumococcal vaccination (the Vietnam Pneumococcal Project): protocol of a randomised controlled trial

Journal:	*BMJ Open*
Manuscript ID	bmjopen-2017-019795.R2
Article Type:	Protocol
Date Submitted by the Author:	21-Mar-2018
Complete List of Authors:	Temple, Beth; Menzies School of Health Research, Global Health Division; London School of Hygiene and Tropical Medicine, Epidemiology and Population Health<br>Toan, Nguyen; Pasteur Institute of Ho Chi Minh City, Department of Disease Control and Prevention<br>Uyen, Doan ; Pasteur Institute of Ho Chi Minh City, Department of Disease Control and Prevention<br>Balloch, Anne; Murdoch Childrens Research Institute, Pneumococcal Research<br>Bright, Kathryn; Murdoch Childrens Research Institute, Pneumococcal Research<br>Cheung, Yin Bun; Duke-NUS Medical School, Centre for Quantitative Medicine; University of Tampere and Tampere University Hospital, Centre for Child Health Research<br>Licciardi, Paul; Murdoch Childrens Research Institute, Pneumococcal Research; University of Melbourne, Department of Paediatrics<br>Nguyen, Cattram; Murdoch Childrens Research Institute, Pneumococcal Research; University of Melbourne, Department of Paediatrics<br>Phuong, Nguyen; Pasteur Institute of Ho Chi Minh City, Department of Disease Control and Prevention<br>Satzke, Catherine; Murdoch Childrens Research Institute, Pneumococcal Research; University of Melbourne, Department of Paediatrics<br>Smith-Vaughan, Heidi; Menzies School of Health Research, Child Health Division<br>Vu, Thi Que Huong; Pasteur Institute of Ho Chi Minh City, Microbiology and Immunology<br>Huu, Tran; Pasteur Institute of Ho Chi Minh City, Department of Disease Control and Prevention<br>Muholland , Kim; London School of Hygiene and Tropical Medicine, Epidemiology and Population Health; Murdoch Childrens Research Institute, Pneumococcal Research
**Primary Subject Heading**:	Global health
Secondary Subject Heading:	Immunology (including allergy), Epidemiology, Infectious diseases, Paediatrics
Keywords:	Clinical trials < THERAPEUTICS, Paediatric infectious disease &

	immunisation < PAEDIATRICS, MICROBIOLOGY, Epidemiology < INFECTIOUS DISEASES

SCHOLARONE™
Manuscripts

For peer review only

Title

Evaluation of different infant vaccination schedules incorporating pneumococcal vaccination (The Vietnam Pneumococcal Project): protocol of a randomised controlled trial

Corresponding author

Beth Temple
Menzies School of Health Research
PO Box 41096
Casuarina NT 0811
Australia
+61 8 8946 8600
beth.temple@menzies.edu.au

Co-authors

TEMPLE, Beth[1, 2]
TOAN, Nguyen Trong[4]
UYEN, Doan Y[4]
BALLOCH, Anne[3]
BRIGHT, Kathryn[3]
CHEUNG, Yin Bun[5, 6]
LICCIARDI, Paul[3, 7]
NGUYEN, Cattram Duong[3, 7]
PHUONG, Nguyen Thi Minh[4]
SATZKE, Catherine[3, 7]
SMITH-VAUGHAN, Heidi[8]
VU, Thi Que Huong[9]
HUU, Tran Ngoc[4]
MULHOLLAND, Edward Kim[2, 3]

[1]Global Health, Menzies School of Health Research, Darwin, Australia
[2]Epidemiology and Population Health, London School of Hygiene & Tropical Medicine, London, UK
[3]Pneumococcal Research, Murdoch Childrens Research Institute, Melbourne, Australia
[4]Department of Disease Control and Prevention, Pasteur Institute of Ho Chi Minh City, Ho Chi Minh City, Vietnam
[5]Centre for Quantitative Medicine, Duke-NUS Medical School, Singapore, Singapore
[6]Centre for Child Health Research, University of Tampere and Tampere University Hospital, Tampere, Finland
[7]Department of Paediatrics, University of Melbourne, Melbourne, Australia
[8]Child Health, Menzies School of Health Research, Darwin, Australia
[9]Microbiology and Immunology, Pasteur Institute of Ho Chi Minh City, Ho Chi Minh City, Vietnam

Word count: 6,416 words

For peer review only

ABSTRACT

Introduction: The World Health Organization (WHO) recommends the use of pneumococcal conjugate vaccine (PCV) as a priority. However there are many countries yet to introduce PCV, especially in Asia. This trial aims to evaluate different PCV schedules and to provide a head-to-head comparison of PCV10 and PCV13, in order to generate evidence to assist with decisions regarding PCV introduction. Schedules will be compared in relation to their immunogenicity and impact on nasopharyngeal carriage of *Streptococcus pneumoniae* and *Haemophilus influenzae*.
Methods and analysis: This randomised, single-blind controlled trial involves 1200 infants recruited at 2 months of age to one of six infant PCV schedules: PCV10 in a 3+1, 3+0, 2+1 or two-dose schedule; PCV13 in a 2+1 schedule; and controls that receive two doses of PCV10 and 18 and 24 months. An additional control group of 200 children is recruited at 18 months that receive one dose of PCV10 at 24 months. All participants are followed up until 24 months of age. The primary outcome is the post-primary series immunogenicity, expressed as the proportions of participants with serotype-specific antibody levels ≥0.35μg/mL for each serotype in PCV10.
Ethics and dissemination: Ethical approval has been obtained from the Human Research Ethics Committee of the Northern Territory Department of Health and Menzies School of Health Research (EC00153) and the Vietnam Ministry of Health Ethics Committee. The results, interpretation and conclusions will be presented to parents and guardians, at national and international conferences, and published in peer-reviewed open access journals.
Trial registration details: NCT01953510

Strengths and limitations of this study

- This study is specifically designed to address two independent questions within a single study: which schedule to use for the provision of PCV, and which PCV to use.
- This study includes a head-to-head comparison of the two licensed PCVs, allowing a direct assessment of their relative immunogenicity and impact on nasopharyngeal carriage.
- The primary outcome is the criteria used for the licensing and varying of PCV schedules.
- This study has relatively low power for the secondary nasopharyngeal carriage outcomes, so the ability to draw conclusions relating to these outcomes is vulnerable in the event of lower than anticipated carriage rates.

For peer review only

ADMINISTRATIVE INFORMATON

Title
Evaluation of different infant vaccination schedules incorporating pneumococcal vaccination (the Vietnam Pneumococcal Project)

Trial registration
ClinicalTrials.gov: NCT01953510

Trial registration - data set

Data category	Information
Primary registry and trial identifying number	ClinicalTrials.gov NCT01953510
Date of registration in primary registry	25 September 2013
Secondary identifying numbers	09/19, 10PN-PD-DIT-079
Source(s) of monetary or material support	National Health and Medical Research Council, Australia<br>Bill & Melinda Gates Foundation<br>GlaxoSmithKline Biologicals SA
Primary sponsor	Murdoch Childrens Research Institute, Australia
Contact for public queries	Professor Kim Mulholland<br>kim.mulholland@lshtm.ac.uk
Contact for scientific queries	Professor Kim Mulholland<br>kim.mulholland@lshtm.ac.uk
Public title	Trial of pneumococcal vaccine schedules in Ho Chi Minh City, Vietnam
Scientific title	Evaluation of different infant vaccination schedules incorporating pneumococcal vaccination (the Vietnam Pneumococcal Project)
Countries of recruitment	Vietnam
Health condition(s) or problem(s) studied	Pneumococcal vaccination responses
Intervention(s)	Active Comparator A: PCV10 administered at 2, 3, 4 and 9 months of age (3+1)
	Experimental B: PCV10 administered at 2, 3 and 4 months of age (3+0)
	Experimental C: PCV10 administered at 2, 4 and 9 months of age (2+1)
	Experimental D: PCV10 administered at 2 and 6 months of age (2 dose)
	Experimental E: PCV13 administered at 2, 4 and 9 months of age (2+1 PCV13)
	Control F: No infant PCV vaccination; PCV10 administered at 18 and 24 months of age
	Control G: Recruited at 18 months of age, non-randomised; PCV10 administered at 24 months of age
Key inclusion and exclusion criteria	Inclusion:<br>• Aged between 2 months and 2 months plus 2 weeks (Arms A-F) or aged between 18m and 18m plus 4 weeks

For peer review only - http://bmjopen.bmj.com/site/about/guidelines.xhtml

	(Arm G)<br>• No significant maternal or perinatal history<br>• Born at or after 36 weeks gestation<br>• Written and signed informed consent from parent/legal guardian<br>• Lives within approximately 30 minutes of the commune health centre<br>• Family anticipates living in the study area for the next 22 months (Arms A-F) or 6 months (Arm G)<br>• Has received three doses of either Quinvaxem or Infanrix-hexa in infancy (Arm G)<br>•
	Exclusion:<br>• Known allergy to any component of the vaccine<br>• Allergic reaction or anaphylactic reaction to any previous vaccine<br>• Known immunodeficiency disorder<br>• Known HIV-infected mother<br>• Known thrombocytopenia or coagulation disorder<br>• On immunosuppressive medication<br>• Administration or planned administration of any immunoglobulin or blood product since birth<br>• Severe birth defect requiring ongoing medical care<br>• Chronic or progressive disease<br>• Seizure disorder<br>• History of invasive pneumococcal, meningococcal or *Haemophilus influenzae* type b diseases, or tetanus, measles, pertussis or diphtheria infections<br>• Receipt of any 2 month vaccines through the EPI program (Arms A-F), or receipt of PCV (Arm G);<br>Family plans on giving the infant the Quinvaxem (DTP-Hib-HBV) or OPV vaccines (Arms A-F)
Study type	Interventional, randomised, parallel group, open label phase II/III trial (Arms A-F). Non-randomised (Arm G). Outcomes assessors (laboratory) blinded. Purpose: prevention.
Enrolment period	Arms A-F: 30 September 2013 - 8 January 2015<br>Arm G: 14 April 2015 - 12 May 2016
Sample size	Target: 1400<br>Number enrolled: 1400
Recruitment status	Active, not recruiting
Primary outcome	Proportion of children with IgG antibody concentration ≥0.35μg/mL for individual pneumococcal serotypes, four weeks post-primary series, measured by ELISA
Key secondary outcomes	Geometric mean concentration (GMC) of serotype-specific IgG, four weeks post-primary series, measured by ELISA
	Proportion of children with IgG antibody concentration ≥0.35μg/mL and GMCs, four weeks post-booster, measured by ELISA
	Proportion of children with serotype-specific opsonisation indices ≥8, four weeks post-primary series and four weeks post-booster, measured by opsonophagocytic assay
	Median number of serotype-specific antibody secreting memory B cells, four weeks post-booster and at 18 months of

For peer review only

	age, measured by ELISPOT
	Proportion of children carrying pneumococcus (any pneumococci, capsular pneumococci, or vaccine-type pneumococci) in the nasopharynx at 12 months of age, measured by culture and latex agglutination serotyping
	Proportion of children carrying NTHi in the nasopharynx at 12 months of age, measured by culture and PCR
Ethics Review	Approved by the Human Research Ethics Committee of the Northern Territory Department of Health and Menzies School of Health Research (EC00153) and the Vietnam Ministry of Health Ethics Committee

Protocol version

Protocol version 10.0 dated 3 June 2015 with Letter of Amendment Number 1 dated 1 September 2016

Revision chronology
Original: Version 3.1, 5 June 2013
First amendment: Version 5.0, 21 April 2014. Main reason for amendment: the Vietnam Ministry of Health (MOH) does not permit the co-administration of measles vaccine and *Infanrix-hexa* vaccine. Measles, *Infanrix-hexa* and PCV were scheduled to be given at 9 months of age in Arms C and E. An additional visit at 9.5 months of age was added for these groups, for receipt of PCV and *Infanrix-hexa*.
Second amendment: Version 7.0, 8 December 2014. Main reason for amendment was that additional funding was secured to: extend the follow up of all participants from 18 to 24 months of age; evaluate a single dose of PCV10 at 18 months of age; and recruit an additional control group at 18 months of age (Arm G) to provide a comparator for the original control group (Arm F). Version 7.0 was never implemented (see below).
Third amendment: Version 9.0, 4 March 2015. Main reason for amendment: to incorporate minor clarifications to version 7.0 requested during review by MOH. These changes did not affect participant recruitment or follow-up and the version number was only changed at the request of MOH.
Fourth amendment: Version 10.0, 3 June 2015. Main reason for amendment: to incorporate additional minor clarifications to version 9.0 requested during review by the Vietnam Ministry of Health. These changes did not affect participant recruitment or follow-up and the version number was only changed at the request of MOH.

Roles and responsibilities

Sponsor contact information
Trial Sponsor: Murdoch Childrens Research Institute, Royal Children's Hospital, Flemington Road, Parkville, Victoria 3052, Australia
Telephone: +61 3 8341 6200
Contact name: Professor Kim Mulholland

Sponsor and funder
GSK was consulted during the design of the trial. None of the funders have any role in the trial conduct, trial management, laboratory tests, or data analyses.

For peer review only

INTRODUCTION

Background and rationale

Streptococcus pneumoniae (pneumococcus) remains a leading vaccine preventable cause of serious infection in young children, despite the availability of effective vaccines. The first infant pneumococcal vaccine, the 7-valent pneumococcal conjugate vaccine (PCV7), was licensed in the United States in the year 2000. Introduction of PCV7 has been associated with dramatic reductions in pneumococcal disease.[1-3] However, geographical variation in serotype distribution[4-7] and an increase in invasive pneumococcal disease (IPD) caused by non-PCV7 serotypes following vaccine introduction[8] necessitated the development of higher valency PCVs.

There are currently two licensed PCVs: PCV10, a 10-valent pneumococcal vaccine that uses non-typeable *Haemophilus influenzae* (NTHi) protein D as a carrier protein for eight of the ten serotypes (*Synflorix™*, PHiD-CV, GSK); and PCV13, a 13-valent pneumococcal CRM_{197} conjugate vaccine (*Prevnar-13™/Prevenar-13™*, Pfizer). Both have been shown to be non-inferior to PCV7 in terms of post-primary series immunogenicity for the shared serotypes.[9-11] Despite the availability of both PCV10 and PCV13 for several years, there have been no published studies to date directly comparing their post-primary series immunogenicity or impact on nasopharyngeal (NP) carriage.

The cost of PCVs is a major barrier to vaccine introduction in low to middle-income countries; therefore investigation of alternative schedules with a reduced number of doses is of great importance. The uptake of PCV introduction in Asia has been particularly slow. Three schedules are currently in routine use around the world for PCV introduction: a 3+1 schedule (a three-dose primary series followed by a booster dose in the second year of life); a 3+0 schedule (a three-dose primary series without a booster dose); and a 2+1 schedule (a two-dose primary series followed by a booster dose in the second year of life). Data from periods of PCV7 shortage in the United States show high vaccine effectiveness of a two-dose primary series against invasive pneumococcal disease (IPD),[12 13] and trial data of CRM_{197}-conjugated PCVs show comparable immunogenicity following a two- or three-dose primary series, although antibody levels to serotypes 6B and 23F tend to be lower after two doses.[14 15] Trials of PCV10 and PCV13 also support the use of a two-dose

For peer review only

primary series. A trial of PCV10 in Europe directly comparing the immunogenicity of a two- and three-dose primary series showed a similar proportion of participants achieving protective antibody levels (≥0.2µg/mL) for all ten serotypes.[16] In a trial of PCV13 in Mexico, over 93% of participants achieved protective antibody levels (≥0.35µg/mL) for most of the 13 serotypes following two doses, with the exception of serotypes 6B and 23F.[17] Four trials in Europe directly comparing PCV13 and PCV7 responses showed comparable immune responses between the vaccines following two doses.[18]

In developing countries, a 2+1 schedule with a booster dose in the first year of life may be advantageous. This modified schedule would likely increase compliance, would provide full immunisation closer to the peak incidence of pneumococcal disease, and could enable the booster dose to coincide with measles vaccination. Alternatively, a further reduced PCV schedule with only two doses may be optimal for pneumococcal vaccination. Our previous trial in Fiji showed that protective antibody levels were reached for five of the seven serotypes following a single dose of PCV7 at 14 weeks of age.[15] Furthermore, a booster dose of the 23-valent pneumococcal polysaccharide vaccine at 12 months of age was more immunogenic following a single dose primary series of PCV7 compared with a two or three dose primary series for four serotypes, and comparable for the other three serotypes.[19] A trial of PCV9 from South Africa also showed that one dose at six weeks of age elicited a significant response for seven serotypes,[20] and modelling data from the US suggest that a single dose of PCV could prevent up to 62% of IPD.[21] More recently, in the UK, where routine infant PCV vaccination has been in place for over 10 years, a 1+1 schedule of PCV13 was shown to elicit equivalent or superior post-booster responses compared with a 2+1 schedule for nine serotypes.[22]

Carriage of pneumococci in the nasopharynx is commonly a prerequisite for IPD, and is the usual means of transmission of the bacteria. The herd effect of pneumococcal vaccination is mediated by the impact on NP carriage.[23] Vaccination with PCVs generally results in a decrease in vaccine type (VT) pneumococcal carriage, which is most commonly observed after a booster dose and often accompanied by a compensatory increase in non-VT carriage.[23-27] There have been few trials that evaluate the effect of different PCV schedules on carriage. A trial from the Netherlands showed that a two-dose primary series with or without a booster reduced VT carriage at 12 months of age compared with controls.[28] VT carriage was further reduced at 18 months in the group that received the booster dose,

For peer review only

compared with the group that did not receive the booster, although this difference did not persist at 24 months of age. Similarly, our trial in Fiji showed that a two or three dose primary series with or without a booster reduced VT carriage at 12 months of age compared with controls, but no difference was seen at 17 months of age (F Russell, personal communication).

It has been hypothesised that the Protein D carrier in PCV10 may result in an impact on *H. influenzae* carriage. A recent review of the impact of Protein D-containing PCVs on NTHi carriage concludes that any such impact is likely to be small and transient, although changes in the density of carriage are yet to be evaluated. Two large phase III trials (POET trial of an 11-valent PCV and COMPAS trial of PCV10) showed trends towards a reduction in NTHi carriage following a booster dose of PCV, along with a trial of PCV10 in toddlers in Kenya; but other trials conducted in Finland, the Netherlands and the Czech Republic showed no impact of PCV10 on NTHi carriage.[29]

This trial includes six infant vaccination schedules: four different PCV10 schedules (Arm A, a 3+1 schedule at 2, 3, 4 and 9 months of age; Arm B, a 3+0 schedule at 2, 3 and 4 months; Arm C, a 2+1 schedule at 2, 4 and 9 months; and Arm D, a 2-dose schedule at 2 and 6 months); a 2+1 PCV13 schedule at 2, 4 and 9 months (Arm E); and a control group that receives two doses of PCV10 at 18 and 24 months (Arm F). In response to more recent interest in schedules with only one or two doses of PCV, which may be sufficient to maintain herd immunity at the population level, an additional control group is recruited at 18 months of age for comparison with the initial control group (Arm G).

Explanation for choice of comparators

There was no PCV licensed in Vietnam at the time the protocol was finalised in 2013. The inclusion of control groups that receive no infant doses of PCV is therefore justified. Control group participants recruited in infancy receive two doses of PCV10, at 18 and 24 months of age. Control group participants recruited at 18 months of age receive a single dose of PCV10 at 24 months of age. Intervention group participants receive at least two doses of PCV in the first year of life. All participants receive pneumococcal immunisation that is likely to be effective and is not otherwise available in Vietnam. The specific regimens to be evaluated are based on likely future global recommendations and to directly compare the two licensed PCVs.

For peer review only - http://bmjopen.bmj.com/site/about/guidelines.xhtml

Both PCV10 and PCV13 have been shown to be non-inferior to PCV7 for the serotypes common to both vaccines, and to have the potential to provide protection against the additional serotypes included.[9-11] For both vaccines the most common adverse reactions are redness at the injection site and irritability, which are common following administration of other vaccines. Other adverse reactions may include: drowsiness; temporary loss of appetite; pain, redness or swelling at the injection site; and fever. Such reactions are usually temporary.

Objectives

This trial has been designed to answer two independent questions concurrently, relating to the evaluation of different schedules incorporating PCV10 and the comparison of PCV10 and PCV13:

1) What is the optimal schedule for provision of EPI vaccines with the incorporation of PCV10; and
2) How do the responses to vaccination with PCV10 or PCV13 compare?

The primary endpoint for both study questions is the post-primary series immunogenicity. For this endpoint data from Arms A and B are combined, as they receive an identical three-dose primary series (see Table 1 for a detailed description of the trial arms). The primary analysis for each study question is to assess non-inferiority of the post-primary series immunogenicity (in terms of the proportion of participants achieving protective levels of serotype-specific IgG of ≥0.35μg/mL), using Arms A+B as the comparator (see below for details). Non-inferiority is assessed for each of the ten serotypes in PCV10, and an overall conclusion of non-inferiority drawn if found for at least seven of the ten serotypes.

1) What is the optimal schedule for provision of Expanded Program of Immunisation (EPI) vaccines with the incorporation of PCV10?

Primary objective

The primary objective is to compare a 2+1 schedule at 2, 4 and 9 months of age with a 3+1 schedule at 2, 3, 4 and 9 months of age. The primary hypothesis is that the proportion of participants with protective levels of antibody is non-inferior following a two-dose primary series (Arm C) compared with a three-dose primary series (Arms A+B). The schedules will also be compared in relation to: the Geometric Mean

For peer review only - http://bmjopen.bmj.com/site/about/guidelines.xhtml

Concentrations (GMCs) of IgG and opsonophagocytosis post-primary series; the proportion of participants with protective levels of antibody, the GMCs of IgG and opsonophagocytosis post-booster; the memory B cell responses; and the impact on nasopharyngeal (NP) carriage rates and density of bacteria of interest.

Key secondary objectives

- To investigate an experimental two-dose schedule at 2 and 6 months of age (Arm D), compared with a 3+1 schedule (Arm A+/-B) and a 2+1 schedule (Arm C);
- To assess the impact of a booster dose on NP carriage of pneumococcus and NTHi, comparing a 3+1 schedule (Arm A) with a 3+0 schedule (Arm B) and with unvaccinated controls (Arm F); and
- To evaluate a single dose of PCV10 at 18 months of age (Arm F), compared with unvaccinated controls (Arm G).

2) How do the responses to vaccination with PCV10 or PCV13 compare?

Primary objective

The primary objective is to compare a PCV13 schedule at 2, 4 and 9 months of age with a PCV10 schedule at 2, 3, 4 and 9 months of age. The primary hypothesis is that the proportion of participants with protective levels of antibody is non-inferior following a two-dose primary series of PCV13 (Arm E) compared with a three-dose primary series of PCV10 (Arms A+B). The schedules will also be compared in relation to: the GMCs of IgG and opsonophagocytosis post-primary series; the proportion of participants with protective levels of antibody, the GMCs of IgG and opsonophagocytosis post-booster; the memory B cell responses; and the impact on nasopharyngeal (NP) carriage rates and density of bacteria of interest.

Key secondary objectives

- To compare PCV10 (Arm C) and PCV13 (Arm E) in a 2+1 schedule at 2, 4 and 9 months of age; and
- To compare the responses to a single dose of PCV10 (Arm D) and PCV13 (Arm E).

For peer review only

Additional objectives

Additional objectives relating to the second control group (Arm G) are:

- To evaluate a single dose of PCV10 at 18 months of age, comparing serotype-specific antibody levels in Arms F and G at 18, 19 and 24 months of age; and
- To compare the immunogenicity and reactogenicity of *Infanrix-hexa* at 18 months of age in children who have received 3 doses of *Infanrix-hexa* or *Quinvaxem* in infancy (Arm G).

Trial design

The Vietnam Pneumococcal Project is a single-blind, open-label, randomized controlled phase II/III non-inferiority trial to investigate simplified childhood vaccination schedules that are more appropriate for developing country use. This is a seven-arm trial that includes six different infant vaccination schedules (Arm A-F) and an additional control group (Arm G) recruited at 18 months of age (Table 1). Arm A receives PCV10 at 2, 3, 4 and 9 months of age (3+1); Arm B receives PCV10 at 2, 3 and 4 months (3+0); Arm C receives PCV10 at 2, 4 and 9 months (2+1); Arm D receives PCV10 at 2 and 6 months (2-dose); Arm E receives PCV13 at 2, 4 and 9 months (2+1); Arm F receives two doses of PCV10 at 18 and 24 months; and Arm G receives one dose of PCV10 at 24 months. Participants also receive *Infanrix-hexa* (DTP-Hib-HBV-IPV) instead of the routine EPI vaccine *Quinvaxem* (DTP-Hib-HBV): four doses for participants in Arms A-F and one dose for Arm G participants.

For peer review only

METHODS AND ANALYSIS

Study setting

PCV introduction in Asia has been slow, in part due to a lack of local or regional data on the effect of PCV. We selected the Southeast Asian country of Vietnam as the location for the trial as a country with a strong health system, a track record of conducting relevant clinical trials, and a Government with strong interest both in the trial and in introducing PCV in the near future. Furthermore, trial results from Vietnam are likely to be considered as applicable to other countries in the region. This is the first trial involving infants to take place within Ho Chi Minh City, the largest city in Vietnam. The trial is conducted in two districts, District 4 and District 7. Districts are divided into communes, each of which has a health centre that provides preventive health services including EPI immunizations, along with some primary health care services. The study is conducted in one commune health centre in each district, with participants drawn from the surrounding communes within that district.

Eligibility criteria

Inclusion criteria

Subjects must meet all of the following inclusion criteria in order to be eligible to participate: aged between 2 months and 2 months plus 2 weeks (Arms A-F) or aged between 18 months and 18 months plus 4 weeks (Arm G); no significant maternal or perinatal history; born at or after 36 weeks gestation; written informed consent from the parent/legal guardian; lives within approximately 30 minutes of the commune health centre; anticipates living in the study area for the next 22 months (Arms A-F) or 6 months (Arm G); and received 3 doses of either *Quinvaxem* or *Infanrix-hexa* in infancy (Arm G only).

Exclusion criteria

Subjects meeting any of the following exclusion criteria at baseline will be excluded from study participation: known allergy to any component of the vaccine; allergic or anaphylactic reaction to any previous vaccine; known immunodeficiency disorder; known HIV-infected mother; known thrombocytopenia or coagulation disorder; on immunosuppressive medication; administration or planned administration of any immunoglobulin or blood product since birth; severe birth defect requiring ongoing medical care; chronic or progressive disease; seizure disorder; history of invasive pneumococcal, meningococcal or *H. influenzae* type b diseases, or tetanus,

For peer review only

measles, pertussis or diphtheria infections; receipt of any 2 month vaccines through the EPI program (Arms A-F), or receipt of PCV (Arm G); or family plans on giving the infant *Quinvaxem* (Arms A-F).

Interventions

PCV schedules

Eligible participants recruited in infancy are randomised to one of six different vaccination schedules (Table 1). Participants randomised to Arms A-D receive PCV10 in a: 3+1 schedule at 2, 3, 4 and 9 months of age; a 3+0 schedule at 2, 3 and 4 months of age; a 2+1 schedule at 2, 4 and 9 months of age; or a two-dose schedule at 2 and 6 months of age, respectively. Participants randomised to Arm E receive PCV13 in a 2+1 schedule at 2, 4 and 9 months of age. Control group participants receive PCV10 at 18 and 24 months of age if randomised to Arm F, or PCV10 at 24 months of age if recruited to Arm G at 18 months of age. PCV is administered by intramuscular injection into the anterolateral thigh in children less than 18 months old and in the deltoid muscle of the arm in children aged 18 months and over. All vaccinations are performed by nurses specifically trained in infant vaccine administration.

PCV10

PCV10 (*Synflorix*) is a 10-valent pneumococcal polysaccharide conjugate vaccine using Protein D (a highly conserved surface protein from NTHi) as the main carrier protein. PCV10 is presented as a turbid white suspension in a two-dose vial. One dose consists of 0.5mL of the liquid vaccine, containing 1µg of pneumococcal polysaccharide from serotypes 1, 5, 6B, 7F, 9V, 14 and 23F and 3µg of pneumococcal polysaccharide from serotypes 4, 18C and 19F. Serotypes 1, 4, 5, 6B, 7F, 9V, 14 and 23F are conjugated to Protein D; serotype 18C is conjugated to tetanus toxoid carrier protein; and serotype 19F is conjugated to diphtheria toxoid carrier protein.

PCV13

PCV13 (*Prevnar-13*) is a 13-valent pneumococcal polysaccharide conjugate vaccine using non-toxic diphtheria CRM_{197} carrier protein. PCV13 is presented as a 0.5mL suspension in a single-dose pre-filled syringe. One dose contains approximately 2.2µg of pneumococcal polysaccharide from serotypes 1, 3, 4, 5, 6A, 7F, 9V, 14,

18C, 19A, 19F and 23F and 4.4µg of pneumococcal polysaccharide from serotype 6B.

Criteria for discontinuing or modifying allocated interventions

There is no modification of doses for participants in this study. If a participant has an allergic or anaphylactic response to vaccination they will be withdrawn from the study. Participants may also be withdrawn voluntarily by the parent/legal guardian at any time, or by the study staff if they refuse any further study procedures or develop any of the exclusion criteria during the course of the study.

Strategies to improve and monitor adherence

Scheduled visit dates are noted on a health record card kept by the parent. If a participant does not attend a scheduled visit, a reminder phone call is made from the study clinic. If the participant cannot be contacted directly, their local Commune Health Centre is contacted for further follow up by phone or by home visit.

Relevant concomitant care

Participants receive *Infanrix-hexa*, which is only available on the private market, instead of the routine EPI vaccine *Quinvaxem*. Participants in Arms A-F receive four doses in one of the following schedules: 2, 3, 4 and 19 months (Arms A and B); 2, 4, 9.5 and 19 months (Arms C and E); 2, 4, 6 and 19 months (Arm D); or 2, 3, 4 and 18 months (Arm F); and participants in Arm G receive one dose at 18 months of age. The routine EPI measles and measles-rubella immunisations are also provided during the course of the study: measles at 9 months of age and measles-rubella at 18 (Arms A-E) or 19 (Arms F-G) months of age. Participants allocated to one of the 2+1 vaccination schedules (Arms C and E) receive measles at 9 months of age and receive PCV and *Infanrix-hexa* two weeks later. For visits with two vaccinations, the vaccines are administered in different limbs. Other vaccinations are permitted in this study with a two-week interval from study vaccines, with the exception of *Quinvaxem* in Arms A-F. Other medications are also permitted, with the exception of immunosuppressive medication and medications listed as contraindicated to the study vaccines.

For peer review only - http://bmjopen.bmj.com/site/about/guidelines.xhtml

Outcomes

Primary outcome measure

The primary outcome measure is the concentration of serotype-specific IgG for the ten serotypes common to both PCV10 and PCV13, assessed four weeks post-primary series and measured using a modified 3rd generation standardized ELISA.[30] Primary comparisons between arms are made in terms of the proportion of children with antibody concentration ≥0.35µg/mL for individual serotypes. The cut-off of 0.35µg/mL was determined as a result of a pooled analysis of data from efficacy trials,[31] and is used as the basis for non-inferiority assessments for the approval of new PCVs.[32-34]

Secondary immunogenicity outcome measures

- Serotype-specific IgG antibody concentrations for all PCV13 serotypes are measured by ELISA from all blood samples (Table 1) and are summarised in terms of both the proportion of children with antibody concentration ≥0.35µg/mL and the GMC.
- Opsonisation indices (OI) for all PCV13 serotypes are measured by opsonophagocytic assay (OPA)[35] for 100 participants per intervention group (Arms A-E) four weeks post-primary series and four weeks post-booster, and are summarised in terms of the proportion of participants with OI ≥8 and the Geometric Mean Titre (GMT).
- Polysaccharide specific memory B cells for serotypes 1, 5, 6B, 14, 18C, 19A and 23F are enumerated by ELISPOT[35] for 50 participants per intervention group (Arms A-E) post-booster and at 18 months of age, and for 100 participants per control group (Arms F and G) at 18 and 24 months of age. The results are summarised as the median number of antibody secreting cells.

Nasopharyngeal carriage outcome measures

- NP carriage of pneumococcal serotypes is measured by traditional culture (colonial morphology, α-haemolysis, the optochin test and *lytA* PCR where indicated)[36] and latex agglutination using type-specific antisera at 2, 6, 9 and 12 months of age in all groups and at 18 and 24 months of age in the control groups (Arms F and G). NP carriage and density of pneumococcal serotypes are measured by quantitative real-time PCR (qPCR) targeting *lytA* and microarray at 18 and 24 months of age.[37 38] Overall, capsular, vaccine-type and serotype-specific carriage rates are described. The antimicrobial resistance of

For peer review only

pneumococcal isolates is determined at 12 months of age by CLSI disk diffusion, for oxacillin, erythromycin, trimethoprim/sulphamethoxazole, ofloxacin, clindamycin, vancomycin, tetracycline, and chloramphenicol. E-tests are conducted for penicillin, ceftriaxone, and vancomycin where indicated, and CLSI breakpoints applied.

- NP carriage of *H. influenzae* is measured by traditional culture (colonial morphology, X and V dependence, *SiaT* PCR for discrimination from *H. haemolyticus*, and the Phadebact® Haemophilus coagglutination test) at 12 months of age in all groups, at 6 and 9 months of age in Arms A and C, and from all swabs in the control groups (Arms F and G). Overall density of *H. influenzae* carriage is measured by qPCR targeting *hpd* and *SiaT* diagnostic targets at 18 and 24 months of age.[39 40]

Immunogenicity of *Infanrix-hexa*

Immunogenicity of *Infanrix-hexa* is measured in terms of IgG levels to diphtheria, tetanus, Hib PRP antigen, hepatitis B surface antigen, and *B. pertussis* (PT). IgG levels will be determined by ELISA, using commercial test kits.

An overview of the procedures for collection, transportation and laboratory analyses of the blood and NP samples can be found in Appendix 1.

For peer review only

For peer review only - http://bmjopen.bmj.com/site/about/guidelines.xhtml

Participant timeline

Table 1: Schedule of enrolment, interventions and assessments

Age (months)	2m	3m	4m	5m	6m	7m	9m	9.5m	10m	12m	18m	19m	24m
ENROLMENT:													
Informed consent	X										X[1]		
Eligibility assessment	X										X[1]		
Allocation	X												
INTERVENTIONS:													
PCV10 - Group A	X	X	X				X						
PCV10 - Group B	X	X	X										
PCV10 - Group C	X		X					X					
PCV10 - Group D	X				X								
PCV13 - Group E	X		X					X					
PCV10 - Group F											X		X
PCV10 - Group G													X
ASSESSMENTS:													
Demographics	X										X[1]		
Household characteristics	X										X[1]		
Nasopharyngeal swab	X				X		X			X	X		X
Blood sample - Group A	X[2]			X			X		X		X[2]		
Blood sample - Group B				X	X		X[2]		X		X[2]		
Blood sample - Group C				X	X[2]		X		X		X[2]		
Blood sample - Group D		X			X	X	X[2]				X[2]		
Blood sample - Group E		X[2]		X			X		X		X[2]		
Blood sample - Group F											X	X	X
Blood sample - Group G											X	X	X
General health	X	X	X	X	X	X	X	X	X	X	X	X	X

[1] Group G only. Any events occurring before 18m do not apply to Group G.
[2] Each participant provides only one of these blood samples (the last 50 participants per group enrolled into Groups A-E provide this blood sample at 18 months; the remainder provide it at the other time point)

For peer review only - http://bmjopen.bmj.com/site/about/guidelines.xhtml

For peer review only

Sample size

The target sample size for infant recruitment (Groups A-F) is 1200 with an allocation ratio of 3:3:5:4:5:4, resulting in target group sizes of: A=150, B=150, C=250, D=200, E=250 and F=200. An additional target of 200 children aged 18 months are recruited into Group G. Sample size calculations are based on the primary outcome of post-primary series immunogenicity (proportion of participants with serotype-specific antibody concentrations ≥0.35μg/mL) for each of the two study questions. A non-inferiority margin of 10% difference in absolute risk is deemed clinically significant, as used by regulatory authorities. Non-inferiority is assessed for each of the ten serotypes in PCV10 (comparing Groups A+B with Group C or Group E), and an overall conclusion of non-inferiority is drawn if the alternative hypotheses are accepted for at least seven of the ten serotypes. This sample size provides >99% power for the overall conclusion of non-inferiority with a 5% one-sided type I error rate, estimated by simulation using a tailor-made program written for implementation in Stata with 10,000 replications.[41] Powers for serotype-specific hypotheses range from 83% to >99%, calculated in PASS Software 2002 using the Farrington-Manning (1990) method.[42] Based on findings from our earlier work in Fiji and from data available in the literature,[43-4543-45] the assumed probabilities of antibody concentration ≥0.35μg/mL are: 95% for serotypes 1, 4, 5, 7F, 9V, 14 and 19F; 90% for serotype 18C; 80% for serotype 23F; and 75% for serotype 6B. The within-subject correlation between the multiple binary endpoints is captured by a subject-level variation term with standard deviation 1.7 in a random-effect logistic regression model, and the loss to follow up rate is assumed to be 5% post-primary series and 10% at 12 months of age. The sample size also provides 98% power to detect a difference in post-primary series immunogenicity following two doses of PCV10 or PCV13, defined by a 10% difference in absolute risk based on a Fisher's Exact test (5% two-sided).

Carriage outcomes: The sample size provides 76% and 71% power to detect a difference in NTHi carriage rates at 12 months of age between Groups A and F and Groups A and B, respectively, and 64% and 59% power to detect a difference in vaccine-type pneumococcal carriage rates between Groups A and F and Groups A and B, respectively. Difference in carriage is defined by a relative risk of 0.6. The calculations were based on Fisher's Exact tests (5% one-sided), assuming carriage rates in Group F (controls) of 30% for NTHi and 24% for vaccine-type pneumococci, based on data from Vietnam (L Yoshida, personal communication).

For peer review only

Recruitment

Participants in Groups A-F are recruited from infants born in the study communes during the enrolment period. Commune health centre staff identify potential participants from the commune health centre birth records. Based on the expected number of births, around a quarter of infants born in the study communes need to be enrolled to complete recruitment within the target enrolment period of 12 months. Recruitment rates will be monitored on a monthly basis and meetings held with study staff and commune health centre staff to discuss any significant declines in recruitment rates. Commune health centre staff visit the home of potential participants when the infant is approximately six weeks old and provide verbal and written information about the trial, in Vietnamese. Those interested in participating are referred to the study clinic when the infant is approximately two months old. At this time, written informed consent is obtained (Appendix 2), after which a study nurse/doctor examines the infant to ensure that all the eligibility criteria are met. Participants in Group G are recruited from children turning 18 months old in the study communes in parallel to the children in Groups A-F turning 18 months.

Allocation

The allocation sequence for Groups A-F is produced using a computer-generated list of random numbers using a block randomisation scheme, stratified by district. The group allocation is contained within a sealed envelope at the study clinic, with sequential ID numbers written on the outside of the envelope. The allocation sequence is generated at Menzies School of Health Research. A study doctor will enrol participants and assign them to a study group by selecting the next available envelope. The envelope is not opened until after completion of the informed consent and eligibility assessment processes.

Blinding

All laboratory staff are blinded to the study group allocation as the key outcome measures that address the study objectives are all laboratory based. Laboratory samples are labelled with the ID number, which does not identify the study group. Given the different timing of the vaccination schedules in the different groups, the study nurses, vaccine administrators and participants will not be blinded to the study group allocation.

For peer review only

Data collection methods

Standardised carbon copy data collection forms are used and are completed by dedicated, trained study staff. The original is transported to the trial office for data entry, with the carbon copy filed at the clinic. Blood samples and NP swabs are collected by staff specifically trained in the collection of samples from infants, and the volume of blood collected and the swab quality are recorded.

Retention: Appointments are documented on a parent-held health record card and a reminder phone call made the week before the scheduled visit. If a participant fails to attend an appointment, a follow up phone call is made to rebook the visit. Participants are given a small payment towards the transport costs of coming to the clinic for each study visit. Participants who miss a study visit will continue to be followed up for both sample collection and vaccine administration where possible, with attempts made to contact such participants until such time as they would have completed the study.

Data management

Data collection forms are double-entered by dedicated data entry staff into pre-coded EpiData version 3.1 files with built in range and consistency checks. Entered data are validated monthly and then uploaded to a central Microsoft Access database, stored on a secure server. Immunology results are double-entered in a Microsoft Excel spreadsheet. NP culture results are entered in a Microsoft Access database and qPCR and microarray results exported from SentiNET into a Microsoft Excel database. The data collection forms and laboratory results are linked at the time of analysis.

Statistical methods

Analysis of primary and secondary outcomes

For each of the two study questions, the primary objective is to compare a 2+1 schedule of 1) PCV10 and 2) PCV13, with a 3+1 schedule of PCV10. The primary outcome is the proportion of participants with serotype-specific antibody concentrations ≥0.35µg/mL, four weeks post-primary series (at 5 months of age). Data from Arms A and B are combined to form the three-dose post-primary series group. The primary analyses assess the non-inferiority of: 1) two doses of PCV10 at 2 and 4 months of age (Arm C) compared with three doses at 2, 3 and 4 months of age (Arms A+B); and 2) two doses of PCV13 at 2 and 4 months of age (Arm E) compared with three doses of PCV10 at 2, 3 and 4 months of age (Arms A+B). The

For peer review only

proportion of children achieving protective levels of serotype-specific IgG (≥0.35µg/ml) four weeks post-primary series is determined for each of the ten PCV10 serotypes. The non-inferiority margin is defined by a 10% difference in absolute risk. The serotype-specific risk differences (Arm A+B - Arm C) with 90% CIs are calculated using the Newcombe Score method, and the null hypothesis rejected if the upper bound of the CI is <10%. Overall non-inferiority is declared if at least seven of the ten individual null hypotheses are rejected at one-sided 5% level of significance. Secondary data analyses to address the primary objective include the ratio of GMCs post-primary series (Arm C / Arms A+B and Arm E / Arms A+B) with 95% CIs, and the booster response analysed by ANCOVA, adjusting for pre-booster levels.

Analysis of key secondary objectives for study question 1:

- A single dose of PCV10 at 2 months of age (Arm D) will be assessed for non-inferiority to three doses at 2, 3 and 4 months of age (Arms A+B), as described for the primary objective
- The impact of a booster dose on pneumococcal and NTHi carriage will be assessed at 12 months of age. Overall pneumococcal, capsular pneumococcal, PCV10 type (with/without 6A and 19A) and NTHi carriage rates will be determined. Proportions will first be compared between the 3+1 group (Arm A) and the control group (Arm F), using Fisher's Exact test. Where significant differences are found, rates will then be compared between the 3+0 group (Arm B) and controls and between the 3+1 and 3+0 groups.

Analysis of key secondary objectives for study question 2:

- The immunogenicity of two doses of PCV10 or PCV13 will be compared in relation to the proportion of participants with serotype-specific antibody concentrations ≥0.35µg/mL (to the ten shared serotypes), four weeks post-primary series (at 5 months of age). A significant difference will be indicated by a 10% difference in absolute risk, comparing PCV10 (Arm C) with PCV13 (Arm E), and an overall difference will be declared if at least 7 of the 10 individual null hypotheses are rejected and the 7 differences are in the same direction.
- The immunogenicity of a single dose of PCV10 or PCV13 will be compared, as described for the immunogenicity of two doses.

Additional analyses

For peer review only - http://bmjopen.bmj.com/site/about/guidelines.xhtml

Descriptive analyses at the group level will be conducted on the OPA, ELISPOT and microarray data.

Populations of analysis

Analyses will be on a per-protocol population. The primary non-inferiority analyses will be repeated on an intention-to-treat population (ITT), with all participants analysed in the group they were randomised to. Any differences between the per-protocol and ITT analyses will be reported. For each outcome, all available data will contribute to the analyses. To investigate whether data are missing completely at random, we will explore whether attrition varies across the study arms based on baseline covariates. If differential attrition is dependent on baseline variables, we will use a modelling approach to adjust for any such baseline factors and we will present the adjusted results along with the primary analysis.

Additional populations of analysis

- OPAs will be conducted on a subset of 100 participants per group. The first 100 participants per group with both post-primary series and post-booster blood samples available will contribute to the OPA analysis.
- B cell assays will be conducted on a subset of 50 participants per group for Arms A-E and 100 participants per group for Arms F and G. The last 50/100 participants enrolled per group will have blood samples collected for the B cell analysis.

Data monitoring

Data monitoring committee: Safety oversight is under the direction of an independent Data Safety and Monitoring Board (DSMB), in accordance with a DSMB Charter kept in the trial office. The DSMB will meet approximately three times a year to review aggregate and individual participant data related to safety, data integrity and overall conduct of the trial, including a detailed review of all Serious Adverse Events (SAEs). Interim analyses and stopping guidelines: No interim analyses are planned. Stopping guidelines are based on safety. An extraordinary meeting of the DSMB will be called in the event that serious safety issues emerge, to provide recommendations regarding termination of the trial. A final decision to terminate rests with the Principal Investigators and the Sponsor.

For peer review only

For peer review only - http://bmjopen.bmj.com/site/about/guidelines.xhtml

Harms

Data on SAEs will be collected throughout the study, with parents asked about hospitalisations and significant signs and symptoms at each study visit and through a regular review of hospital records. Details of any SAEs will be recorded on the standard reporting form from the Vietnam Ministry of Health and reported to the Principal Investigators and the Ethics Committees. Participants will be kept under observation for 30 minutes following vaccine administration to monitor for any adverse reactions, and information on reactogenicity in the 72 hours following vaccine administration will be recorded on parent held diary cards.

Auditing

External site monitoring will be provided by FHI360, to independently assess protocol and GCP compliance. Monitoring visits will occur at study initiation, close-out and approximately twice a year in each study clinic. 100% of Informed Consent Forms and SAEs and a random selection of approximately 20% of participant folders will be monitored, along with the Trial Regulatory File and laboratory records.

Patient and public involvement

Patients were not involved in the development, design, recruitment or conduct of the study. Community consultation took place at the district level during the design phase, as well as discussion and approval of the design from the district and city level Ministry of Health and the People's Committee of Ho Chi Minh City. Participants will be informed of the overall study results by post, with a postal address collected at the final study visit.

ETHICS AND DISSEMINATION

Research ethics approval

The protocol, the Plain Language Statement (PLS) and the Informed Consent Form (ICF) have approval from the Institutional Review Board at the Pasteur Institute of Ho Chi Minh City, the Vietnam Ministry of Health Ethical Review Committee and the Human Research Ethics Committee of the Northern Territory Department of Health and the Menzies School of Health Research. Both Ethics Committees receive annual reports on the trial progress, for continuing approval of the trial.

Protocol amendments

For peer review only

Any modifications to the protocol that may impact on the conduct of the study will be documented in a formal protocol amendment and approved by both Ethics Committees prior to implementation of the changes. The modified protocol will be given a new version number and date. The Ethics Committees will also be notified of any minor corrections/clarifications or administrative changes to the protocol, which will be documented in a protocol amendment letter. Significant protocol changes will also be updated in the ClinicalTrials.gov record.

Consent

Obtaining consent: The consent process is undertaken by specifically trained study staff. The study staff will go through the PLS and ICF, translated into Vietnamese, in detail with the potential participant's parent/legal guardian. The study staff will then discuss the trial further and answer any questions that may arise. Written informed consent is required prior to enrolment of the infant into the study. Consent is obtained from the parent/legal guardian as the participants are too young to provide consent themselves. A copy of the PLS and ICF will be given to the parent/legal guardian for their records.

Ancillary studies: Specific consent for the indefinite storage of blood and NP samples for future research related to the trial will be obtained from the parent/legal guardian and recorded on the ICF. Any future research will undergo ethical review. Any samples for which indefinite storage is not consented to will be destroyed at the close of the trial.

Confidentiality

All study-related information will be stored securely and held in strict confidence. All documents kept at the study clinics, including the ICFs and participant folders, are stored in locked cabinets. All documents kept centrally are stored in the trial office, which is kept locked. Electronic data is stored in the trial office and on a secure password protected server. The electronic data and laboratory samples are coded by a unique participant number and do not contain the participant name. Access to participants' information will be granted to FHI360 for monitoring purposes, and to the Ethics Committees or DSMB if required.

Access to data

For peer review only - http://bmjopen.bmj.com/site/about/guidelines.xhtml

The final trial dataset will be under the custody of the trial sponsor, MCRI. The Principal Investigator, trial manager and trial statistician will have access to the full anonymised final dataset.

Ancillary and post-trial care

Participants are advised to come to the study clinic for ancillary care, or to Children's Hospital Number 2 in Ho Chi Minh City, where they will not be charged for treatment and services. All participants are covered by clinical trials insurance for trial related harms.

Dissemination policy

Plans

Participants will be informed of the overall study results by post, with a postal address collected at the final study visit. Following completion of the trial, the results will be submitted for publication in peer-reviewed journals, and presented at relevant international conferences. Agreements between MCRI and each of the Pasteur Institute of Ho Chi Minh City and GSK Biologicals SA provide that a party must obtain the prior approval of the other parties in advance of submitting a manuscript for publication, and that such approval will not be unreasonably withheld.

Authorship

A publication subcommittee will consider all proposed publications, with the final decision on content and authorship resting with the Principal Investigator. The role of each author will be published. Group authors may be used where appropriate. There are no plans for the use of professional writers.

Reproducible research

There are no plans to grant public access to the full protocol, participant-level dataset or statistical code.

For peer review only

References

1. Myint TT, Madhava H, Balmer P, et al. The impact of 7-valent pneumococcal conjugate vaccine on invasive pneumococcal disease: a literature review. *Advances in therapy* 2013;30:127-51. doi:10.1007/s12325-013-0007-6.
2. Conklin L, Loo JD, Kirk J, et al. Systematic review of the effect of pneumococcal conjugate vaccine dosing schedules on vaccine-type invasive pneumococcal disease among young children. *Pediatr Infect Dis J* 2014;33 Suppl 2:S109-18. doi:10.1097/INF.0000000000000078.
3. Loo JD, Conklin L, Fleming-Dutra KE, et al. Systematic review of the effect of pneumococcal conjugate vaccine dosing schedules on prevention of pneumonia. *Pediatr Infect Dis J* 2014;33 Suppl 2:S140-51. doi:10.1097/INF.0000000000000082.
4. Kalin M. Pneumococcal serotypes and their clinical relevance. *Thorax* 1998;53:159-62.
5. Hausdorff WP, Feikin DR, Klugman KP. Epidemiological differences among pneumococcal serotypes. *The Lancet. Infectious diseases* 2005;5:83-93. doi:10.1016/S1473-3099(05)01280-6.
6. Hausdorff WP, Bryant J, Paradiso PR, et al. Which pneumococcal serogroups cause the most invasive disease: implications for conjugate vaccine formulation and use, part I. *Clin Infect Dis* 2000;30:100-21. doi:10.1086/313608.
7. Hausdorff WP, Bryant J, Kloek C, et al. The contribution of specific pneumococcal serogroups to different disease manifestations: implications for conjugate vaccine formulation and use, part II. *Clin Infect Dis* 2000;30:122-40. doi:10.1086/313609.
8. Weinberger DM, Malley R, Lipsitch M. Serotype replacement in disease after pneumococcal vaccination. *Lancet* 2011;378:1962-73. doi:10.1016/S0140-6736(10)62225-8.
9. Vesikari T, Wysocki J, Chevallier B, et al. Immunogenicity of the 10-valent pneumococcal non-typeable Haemophilus influenzae protein D conjugate vaccine (PHiD-CV) compared to the licensed 7vCRM vaccine. *Pediatr Infect Dis J* 2009;28:S66-76. doi:10.1097/INF.0b013e318199f8ef.
10. Yeh SH, Gurtman A, Hurley DC, et al. Immunogenicity and safety of 13-valent pneumococcal conjugate vaccine in infants and toddlers. *Pediatrics* 2010;126:e493-505. doi:10.1542/peds.2009-3027.
11. Kieninger DM, Kueper K, Steul K, et al. Safety, tolerability, and immunologic noninferiority of a 13-valent pneumococcal conjugate vaccine compared to a 7-valent pneumococcal conjugate vaccine given with routine pediatric vaccinations in Germany. *Vaccine* 2010;28:4192-203. doi:10.1016/j.vaccine.2010.04.008.
12. Whitney CG, Pilishvili T, Farley MM, et al. Effectiveness of seven-valent pneumococcal conjugate vaccine against invasive pneumococcal disease: a matched case-control study. *Lancet* 2006;368:1495-502. doi:10.1016/S0140-6736(06)69637-2.
13. Mahon BE, Hsu K, Karumuri S, et al. Effectiveness of abbreviated and delayed 7-valent pneumococcal conjugate vaccine dosing regimens. *Vaccine* 2006;24:2514-20. doi:10.1016/j.vaccine.2005.12.025.
14. Goldblatt D, Southern J, Ashton L, et al. Immunogenicity and boosting after a reduced number of doses of a pneumococcal conjugate vaccine in infants and toddlers. *Pediatr Infect Dis J* 2006;25:312-9. doi:10.1097/01.inf.0000207483.60267.e7.

For peer review only - http://bmjopen.bmj.com/site/about/guidelines.xhtml

15. Russell FM, Balloch A, Tang ML, et al. Immunogenicity following one, two, or three doses of the 7-valent pneumococcal conjugate vaccine. *Vaccine* 2009;27:5685-91. doi:10.1016/j.vaccine.2009.06.098.
16. Silfverdal SA, Hogh B, Bergsaker MR, et al. Immunogenicity of a 2-dose priming and booster vaccination with the 10-valent pneumococcal nontypeable Haemophilus influenzae protein D conjugate vaccine. *Pediatr Infect Dis J* 2009;28:e276-82. doi:10.1097/INF.0b013e3181b48ca3.
17. Gutierrez Brito M, Thompson A, Girgenti D, et al. Immunogenicity and safety of 13-valent pneumococcal conjugate vaccine in Mexico. *Revista panamericana de salud publica = Pan American journal of public health* 2013;33:414-21.
18. Rodgers GL, Esposito S, Principi N, et al. Immune response to 13-valent pneumococcal conjugate vaccine with a reduced dosing schedule. *Vaccine* 2013;31:4765-74. doi:10.1016/j.vaccine.2013.08.009.
19. Russell FM, Licciardi PV, Balloch A, et al. Safety and immunogenicity of the 23-valent pneumococcal polysaccharide vaccine at 12 months of age, following one, two, or three doses of the 7-valent pneumococcal conjugate vaccine in infancy. *Vaccine* 2010;28:3086-94. doi:10.1016/j.vaccine.2010.02.065.
20. Huebner RE, Mbelle N, Forrest B, et al. Immunogenicity after one, two or three doses and impact on the antibody response to coadministered antigens of a nonavalent pneumococcal conjugate vaccine in infants of Soweto, South Africa. *Pediatr Infect Dis J* 2002;21:1004-7. doi:10.1097/01.inf.0000036012.37665.fe.
21. Barzilay EJ, O'Brien KL, Kwok YS, et al. Could a single dose of pneumococcal conjugate vaccine in children be effective? Modeling the optimal age of vaccination. *Vaccine* 2006;24:904-13. doi:10.1016/j.vaccine.2005.08.092.
22. Goldblatt D, Southern J, Andrews NJ, et al. Pneumococcal conjugate vaccine 13 delivered as one primary and one booster dose (1 + 1) compared with two primary doses and a booster (2 + 1) in UK infants: a multicentre, parallel group randomised controlled trial. *The Lancet. Infectious diseases* 2017. doi:10.1016/S1473-3099(17)30654-0.
23. O'Brien KL, Dagan R. The potential indirect effect of conjugate pneumococcal vaccines. *Vaccine* 2003;21:1815-25. doi:S0264410X02008071 [pii].
24. Kayhty H, Auranen K, Nohynek H, et al. Nasopharyngeal colonization: a target for pneumococcal vaccination. *Expert Rev Vaccines* 2006;5:651-67. doi:10.1586/14760584.5.5.651.
25. Moore MR, Hyde TB, Hennessy TW, et al. Impact of a conjugate vaccine on community-wide carriage of nonsusceptible Streptococcus pneumoniae in Alaska. *J Infect Dis* 2004;190:2031-8. doi:10.1086/425422.
26. Huang SS, Platt R, Rifas-Shiman SL, et al. Post-PCV7 changes in colonizing pneumococcal serotypes in 16 Massachusetts communities, 2001 and 2004. *Pediatrics* 2005;116:e408-13. doi:10.1542/peds.2004-2338.
27. Dunais B, Bruno P, Carsenti-Dellamonica H, et al. Trends in nasopharyngeal carriage of Streptococcus pneumoniae among children attending daycare centers in southeastern France from 1999 to 2006. *Pediatr Infect Dis J* 2008;27:1033-5. doi:10.1097/INF.0b013e31817bb8cf.
28. van Gils EJ, Veenhoven RH, Hak E, et al. Effect of reduced-dose schedules with 7-valent pneumococcal conjugate vaccine on nasopharyngeal pneumococcal carriage in children: a randomized controlled trial. *JAMA* 2009;302:159-67. doi:10.1001/jama.2009.975.
29. Clarke C, Bakaletz LO, Ruiz-Guinazu J, et al. Impact of protein D-containing pneumococcal conjugate vaccines on non-typeable Haemophilus influenzae acute otitis media and carriage. *Expert Rev Vaccines* 2017;16:1-14. doi:10.1080/14760584.2017.1333905.
30. Balloch A, Licciardi PV, Leach A, et al. Results from an inter-laboratory comparison of pneumococcal serotype-specific IgG measurement and critical

For peer review only - http://bmjopen.bmj.com/site/about/guidelines.xhtml

parameters that affect assay performance. *Vaccine* 2010;28:1333-40. doi:10.1016/j.vaccine.2009.11.011.
31. WHO Expert Committee on Biological Standardization. WHO Technical Report Series 927. Geneva: World Health Organisation, 2005.
32. Feavers I, Knezevic I, Powell M, et al. Challenges in the evaluation and licensing of new pneumococcal vaccines, 7-8 July 2008, Ottawa, Canada. *Vaccine* 2009;27:3681-8. doi:10.1016/j.vaccine.2009.03.087.
33. World Health Organization. WHO/Health Canada Consulation on serological criteria for evaluation and licensing of new Pneumococcal vaccines, Ottawa, Canada, 7-8 July 2008. Secondary World Health Organization. WHO/Health Canada Consulation on serological criteria for evaluation and licensing of new Pneumococcal vaccines, Ottawa, Canada, 7-8 July 2008. http://www.who.int/biologicals/publications/meetings/areas/vaccines/pneumococcal/Pneumo Meeting Report FINAL IK 24 Dec 08.pdf?ua=1.
34. World Health Organization, Expert Committee on Biological Standardization. Recommendations to assure the quality, safety and efficacy of pneumococcal conjugate vaccines. Geneva, 19-23 October 2009. . Secondary World Health Organization, Expert Committee on Biological Standardization. Recommendations to assure the quality, safety and efficacy of pneumococcal conjugate vaccines. Geneva, 19-23 October 2009. . http://www.who.int/biologicals/areas/vaccines/pneumo/Pneumo final 23APRIL 2010.pdf?ua=1.
35. Licciardi PV, Toh ZQ, Clutterbuck EA, et al. No long-term evidence of hyporesponsiveness after use of pneumococcal conjugate vaccine in children previously immunized with pneumococcal polysaccharide vaccine. *The Journal of allergy and clinical immunology* 2016;137:1772-79 e11. doi:10.1016/j.jaci.2015.12.1303.
36. Richter SS, Heilmann KP, Dohrn CL, et al. Accuracy of phenotypic methods for identification of Streptococcus pneumoniae isolates included in surveillance programs. *J Clin Microbiol* 2008;46:2184-8. doi:10.1128/JCM.00461-08.
37. Satzke C, Turner P, Virolainen-Julkunen A, et al. Standard method for detecting upper respiratory carriage of Streptococcus pneumoniae: updated recommendations from the World Health Organization Pneumococcal Carriage Working Group. *Vaccine* 2013;32:165-79. doi:10.1016/j.vaccine.2013.08.062.
38. Turner P, Hinds J, Turner C, et al. Improved detection of nasopharyngeal cocolonization by multiple pneumococcal serotypes by use of latex agglutination or molecular serotyping by microarray. *J Clin Microbiol* 2011;49:1784-9. doi:10.1128/JCM.00157-11.
39. Smith-Vaughan H, Byun R, Nadkarni M, et al. Measuring nasal bacterial load and its association with otitis media. *BMC Ear Nose Throat Disord* 2006;6:10. doi:10.1186/1472-6815-6-10.
40. Price EP, Harris TM, Spargo J, et al. Simultaneous identification of Haemophilus influenzae and Haemophilus haemolyticus using real-time PCR. *Future microbiology* 2017;12:585-93. doi:10.2217/fmb-2016-0215.
41. Feiveson A. Power by simulation. *The Stata Journal* 2002;2:107-24.
42. Farrington CP, Manning G. Test statistics and sample size formulae for comparative binomial trials with null hypothesis of non-zero risk difference or non-unity relative risk. *Stat Med* 1990;9:1447-54.
43. Dinleyici EC, Yargic ZA. Current knowledge regarding the investigational 13-valent pneumococcal conjugate vaccine. *Expert Rev Vaccines* 2009;8:977-86. doi:10.1586/erv.09.68.
44. Immunogenicity, safety, and tolerability of 13-valent pneumococcal conjugate vaccine given to infants with routine pediatric vaccinations in Mexico: Interim

For peer review only

Report. Abstract: 7th International Symposium on Pneumococci and Pneumococcal Diseases; 2010; Tel Aviv, Israel.
45. Prymula R, Schuerman L. 10-valent pneumococcal nontypeable Haemophilus influenzae PD conjugate vaccine: Synflorix. *Expert Rev Vaccines* 2009;8:1479-500. doi:10.1586/erv.09.113.

AUTHORS' CONTRIBUTIONS

BT was involved with the study design, led the funding and ethics applications, has been involved in the day-to-day management of the trial and data analysis and drafted the protocol and this manuscript. NTT advised on the study design and location, was involved in the approval processes in Vietnam, and has been involved in the day-to-day management and implementation of the trial. DYU advised on the study design and location and has been involved in the day-to-day implementation of the trial. AB advised on the study design, assisted with the funding applications, and advised on and provided oversight of the immunology laboratory procedures. KB advised on the study design and location and has been responsible for the day-to-day management and implementation of the trial. YBC advised on the study design and funding applications, especially the statistical aspects of the trial. PL advised on the study design, assisted with the funding applications, and advised on and provided oversight of the immunology laboratory procedures. CDN advised on the study design and statistical analysis plan. NTMP advised on the study design and location, was involved in the approval processes in Vietnam, and has been involved in the day-to-day management of the trial. CS advised on the study design, assisted with the funding applications, and advised on and provided oversight of the microbiology laboratory procedures. HSV advised on the study design, assisted with the funding applications, and advised on and provided oversight of the microbiology laboratory procedures. TQHV advised on the study design and advised on and provided oversight of the laboratory procedures at Pasteur. TNH advised on the study design and location, undertook consultations, was involved in the approval processes in Vietnam, and has had overall responsibility for the conduct of the trial in Vietnam as Site Principal Investigator. EKM conceived the study, undertook consultations, provided oversight for the funding and ethics applications, provided oversight for the conduct of the trial and data analysis, and has had overall responsibility for all aspects of the trial as the Principal Investigator. All authors contributed to refinement of the study protocol and reviewed and approved this manuscript.

For peer review only - http://bmjopen.bmj.com/site/about/guidelines.xhtml

FUNDING STATEMENT

This work is supported by the National Health and Medical Research Council of Australia (grant number 566792) and the Bill & Melinda Gates Foundation (grant number OPP1116833). The doses of PCV10 and funding for the opsonophagocytic assays are provided by GlaxoSmithKline Biologicals SA (GSK).

COMPETING INTERESTS STATEMENT

All authors receive salary support from grants from the National Health and Medical Research Council of Australia and/or the Bill and Melinda Gates Foundation. Non-financial support (in the form of PCV10 vaccine doses) and funding for opsonphagocytic assays are provided by GSK Biologicals SA. EKM is a member of the DSMB for a current Novavax trial, for which he receives consulting fees. He has received travel costs from the GSK group of companies for one international conference, and an honorarium from Merck for one advisory group meeting. He does not have any paid consultancies with or receive any research funds from pharmaceutical companies. Members of CS's team have received awards that were funded (but not assessed) by Pfizer. None of the authors have any other competing interests to declare.

APPENDICES

Appendix 1 - Biological Specimens

Appendix 2 - Plain Language Statement and Informed Consent Form. These materials were translated into Vietnamese, and back-translated into English, by FHI360.

For peer review only

APPENDIX 1

Biological Specimens

Specimens include NP swabs, bacterial isolates cultured from NP swabs, serum from whole blood, plasma from whole blood and peripheral blood mononuclear cells (PBMCs). Long-term storage of specimens is at the Pneumococcal Laboratory at MCRI or at the Pasteur Institute of Ho Chi Minh City at -80°C. No genetic or HIV testing will be performed on stored samples and they will not be used to establish a tissue bank. Consent for the long-term storage of samples and their use in potential future studies is recorded on the ICF.

Sample Collection

Blood samples are collected using a butterfly needle into gel vacutainer tubes or sodium heparin vacutainer tubes. The volume of blood collected at different ages is as follows: 2.0ml at 2 months of age; 3.5ml from 3-10 months and 19 months of age; and 3.5ml or 7.5ml at 18 months and 24 months of age, depending on the assays to be conducted. Blood samples collected into gel vacutainer tubes are kept chilled in a cooler box and transported to the Pasteur Institute laboratory the same day. On arrival at the laboratory the samples are centrifuged and the sera divided into up to three aliquots, stored in micro-tubes and frozen at -80°C prior to analysis. For blood samples where plasma cell and memory B cell responses are assessed, samples are collected into sodium heparin vacutainer tubes and transported to the Pasteur Institute laboratory at room temperature the same day. On arrival at the laboratory plasma and PBMCs are separated from each heparinized blood sample by density gradient centrifugation. Plasma are divided into up to four aliquots and stored at -80°C prior to analysis.

NP samples are collected and transported according to standard guidelines.[1] In brief, NP samples are collected using sterile swabs and placed immediately into 1000µL Skim Milk Tryptone Glucose Glycerol (STGG) transport medium. The samples are kept chilled in a cooler box and transported to the Pasteur Institute laboratory the same day. On arrival at the laboratory two aliquots are removed and the aliquots and original sample are frozen at -80°C prior to analysis.

For peer review only - http://bmjopen.bmj.com/site/about/guidelines.xhtml

Serotype-specific IgG

Serotype-specific anti-pneumococcal IgG levels to each of the 13 serotypes in 13v-PCV are measured using a modified 3rd generation standardized ELISA at the Pasteur Institute laboratory.[2] Briefly, microtiter wells are coated with 2.5-10 mg/mL pneumococcal polysaccharide, depending on the serotype. This is diluted in phosphate buffered saline by incubating at 22° C overnight. To neutralize unspecified cell wall polysaccharide antibodies, 1/100 diluted serum samples are incubated overnight with 10 mg/mL of cell wall polysaccharide and 30mg/mL of serotype 22F, before further dilutions. A reference serum (89-SF, Food and Drug Administration, Bethesda MD) is used and incubated overnight with 10 mg/mL of cell wall polysaccharide. Horse radish peroxidase conjugated anti-human IgG and the TMB Peroxidase Substrate system is used for detection. Results are expressed as µg/mL of serotype-specific IgG. Three control sera will be used on each plate to assess inter-assay variation.

Opsonophagocytic Assay (OPA)

OPAs are conducted at the Pneumococcal Laboratory at MCRI.[3] Serial dilutions of a heat-inactivated sera, in Hanks balanced salt solution with Mg^{++}, Ca^{++} and gelatine, are made in a 96-well sterile microtitre plate. Frozen stock of pneumococci are thawed, washed and diluted to 5×10^4 CFU/serotype/mL. Standard bacterial dilutions are added to all wells and the plate incubated at RT for 30 min. At 30 min, baby rabbit complement, thawed just prior to use, followed by HL-60 cells (2×10^7 cells/ml) is added to all test wells. A bacterial control (heat inactivated foetal calf serum in place of human sera and no complement) and complement control (no sera) are included on all plates. Plates are placed on a horizontal shaker and incubated for 45 min at 37°C in 5% CO_2. The reaction is stopped at 45 min by placing the plate on ice. A 10µL aliquot of this mixture is then spotted onto Todd-Hewitt broth–yeast extract (0.5%) agar plates. After application of an overlay THYE agar containing selective antibiotic (Optochin, Spectinomycin, Streptomycin or Trimethoprim) and 2,3,5-Triphenyltetrazolium chloride (TTC), the plates are incubated overnight at 37°C in 5% CO_2. After overnight incubation, plates are counted and the results expressed as opsonisation indices (OI) where the OI is defined as the interpolated dilution of serum that kills 50% of bacteria.

Memory B cells

Analysis of the memory B cell response is undertaken at the Pasteur Institute laboratory, by ELISPOT assay.[3] PBMCs are re-suspended in RPMI Foetal Calf Serum (FCS) at a concentration of $2x10^6$ cells/mL and 100µL added to each well of the culture plate containing an antigen cocktail (Staphylococcus aureus Cowan strain – Pansorbin cells (SAC; 1:5000), 2.5µg/mL CpG and 83ng/mL pokeweed mitogen). Plates are incubated at 37^oC with 5% CO_2 and 95% humidity for 5 days. At day 5, cells are harvested and washed and the cell pellet re-suspended in 1mL RPMI-FCS and counted by trypan blue. Cells are then made up to a final concentration of $2x10^6$ cells/mL for seeding onto antigen-coated ELISPOT plates. Multiscreen hydrophobic polyvinyldene difluoride (PVDF) membrane ELISPOT plates coated with anti-IgG (10µg/mL), tetanus toxoid (5µg/mL), diphtheria toxoid (10µg/mL) or pneumococcal polysaccharides conjugated to methylated human serum albumin at concentrations in the range 10-20µg/mL are sealed and incubated overnight at 4^oC. ELISPOT plates are then washed and blocked with RPMI-FCS for 30 minutes at 37^oC with 5% CO2 and 95% humidity. Cultured cells or *ex vivo* PBMCs are washed and seeded at 200 to $2x10^5$ cells/well of the antigen-coated ELISPOT plates in RPMI-FCS and incubated overnight at 37^oC with 5% CO_2 and 95% humidity. Cells are then washed with PBS-T and bound IgG detected with an alkaline phosphatase-conjugated IgG for 4 hours at RT. ELISPOT plates are washed again before addition of an alkaline phosphatase substrate solution (nitroblue tetrazolium plus 5-bromo-4-chloro-3-indoylphosphate in dimethyl formamide). The reaction is stopped with two washes in distilled water. Cells are visualized and counted using an automated ELISPOT reader and software. The total frequency of IgG-secreting antibody-forming cells (AFCs) is used as the positive control and 1,000 IgG AFCs/10^6 cultured PBMCs is the lower cut-off for inclusion in the analysis. Up to $15x10^6$ cells/mL are used for the memory B cell assay at the Pasteur Institute and the remainder of the PBMCs are cryopreserved in liquid nitrogen in aliquots of 8-$10x10^6$ cells/mL for planned T cell assays.

S. pneumoniae identification and serotyping

Identification of *S. pneumoniae* is conducted in line with WHO guidelines.[1] In brief, 50µl swab is plated onto Columbia colistin-nalidixic acid blood agar plates, and identification is primarily based on colonial morphology (flat, with a dimple, 1-3mm in size), α-haemolysis and optochin sensitivity. One colony, plus any additional colonies if morphologically distinct, is sub-cultured onto horse blood agar with an optochin disc. Any colonies that are optochin resistant or intermediately resistant but

For peer review only - http://bmjopen.bmj.com/site/about/guidelines.xhtml

otherwise appear to be *S. pneumoniae* are subject to *lytA* PCR,[1] following DNA preparation using the InstaGene matrix (BioRad). All presumptive pneumococci are serotyped, primarily by latex agglutination using reagents produced in-house using antisera from the Statens SerumInstitut, as previously described.[4 5] In summary, pneumococcal culture is made to a 4-5 McFarland density standard and then 10µL of the suspension mixed with 10µL of latex reagent on clear glass slides and rotated for 1 minute. A positive test is indicated by aggregation of latex particles and clearing of the suspension. Isolates that do not react with antisera are subject to *lytA* PCR.

H. influenzae identification

Identification of *H. influenzae* is made from 50µl swab plated onto bacitracin-vancomycin-clindamycin-chocolate-agar. One presumptive *H. influenzae* colony, plus any additional colonies if morphologically distinct, is selected. Colonies are identified as grayish, semi-opaque, smooth, flat or convex, 1-3mm in size. Confirmation is initially demonstrated by X and V growth factor dependence. Capsular and NTHi strains are discriminated using the Phadebact® Haemophilus coagglutination test. All NTHi isolates are tested for beta-lactamase production using nitrocefin.[6] Following identification of presumptive NTHi, DNA is extracted using the InstaGene matrix (BioRad)[7] and tested by *siaT* and *hypD* PCR for discrimination between NTHi and *H. haemolyticus*.[8]

Quantification of *H. influenzae* and pneumococcus

DNA is extracted from 100µl of STGG medium using high-throughput systems (MagNA Pure LC, Roche) using the DNA Isolation Kit II (Bacteria, Fungi) (Roche) incorporating enzymatic digestion. Quantification of *H. influenzae* and pneumococci is then performed using real-time quantitative PCR (qPCR).[9] qPCR targeting the *hpd3* and/or *siaT* gene (*H. influenzae*) or *lytA* gene (pneumococcus) is conducted in 25µl reactions containing 2µl of template DNA on a Stratagene Mx3005 machine using Brilliant III Ultra-Fast qPCR Master Mix (Agilent Technologies) according to the manufacturer's instructions. The density of each bacterial species is assessed in comparison to a set of approximately five reference standards run with each assay to give the density of carriage.

Microarray serotyping

Samples that contain pneumococci are tested by DNA microarray as described previously with minor modifications.[4] Following a culture amplification step (on selective agar such as horse blood agar with 5 µg/ml gentamicin), DNA is extracted

For peer review only

using the Qiacube HT platform (Qiagen). When only a single α-haemolytic colony grows, it is sub-cultured before DNA extraction for microarray. DNA is labelled and then hybridised to the Senti-SP microarray (formally BUGS microarray), scanned on an Agilent scanner, and uploaded to Senti-Net (a cloud based software platform). Serotype-specific density is calculated by multiplying pneumococcal density (measured by *lytA* qPCR) by the relative abundance of each serotype (determined by microarray).

Immunogenicity of *Infanrix-hexa*

The specific IgG to *Haemophilus influenzae* type b (Hib) will be measured by ELISA. High binding ELISA plates are coated with Hib polysaccharide (HBO-HA, the PRP capsular linked to human albumin) antigen and incubated at 37°C for 2 hours and then overnight at 4°C. The plates are washed and blocked with 1% Gelatin in PBS, then loaded with dilutions of standards and patient samples. Following two hours incubation at 37°C, the plates are washed and peroxidase-labelled anti-human IgG is added to each well. Bound specific antibody is detected using the substrate TMB. After the substrate reaction, the intensity of the colour developed is proportional to the amount of IgG-specific antibodies detected in the sample. Results for the samples are determined directly using a standard curve and expressed as µg/mL. Three control sera will be used on each plate to assess inter-assay variation.

The specific IgG to tetanus and diphtheria will be measured using a commercial solid phase ELISA (Genzyme Virotech). The wells are coated with antigen. Specific antibodies of the sample bind to the antigen coated wells and are detected by a secondary enzyme conjugated antibody specific for human IgG. After the substrate reaction, the intensity of the colour developed is proportional to the amount of IgG-specific antibodies detected in the sample. Results for the samples are determined directly using a standard curve and expressed as IU/mL. Two control sera will be used on each plate to assess inter-assay variation.

The Hepatitis B surface antibodies will be measured using AxSym analyzer system. Patient serum is incubated with Micro-particles coated with recombinant HbsAg. Antibody present in the serum binds with antigen on the particles. When this reaction mixture is transferred to the matrix cell, the micro-particles bind irreversibly to the glass fibre matrix. Biotinylated rHBsAg is then added forming an antigen-antibody-antigen complex. Anti-Biotin: Alkaline phosphatase conjugate is dispensed onto the matrix cell and binds with any microparticle-bound antigen-antibody-antigen complex.

For peer review only

The matrix cell is washed to remove any unbound antibody and the substrate 4-Methylumbelliferyl Phosphate is added. The alkaline phosphatase-labelled conjugate catalyses the removal of a phosphate group from the substrate, yielding a fluorescent product, 4-Methylumbelliferone. This fluorescent product is measured and the concentration of anti-HBs in the sample is determined from a calibration curve and will be reported in IU/mL. A positive and negative control will be included in each assay.

The specific IgG to *B. pertussis* (PT) will be measured using a commercial solid phase ELISA (Genzyme Virotech). The wells are coated with antigen. Specific antibodies of the sample bind to the antigen coated wells and are detected by a secondary enzyme conjugated antibody specific for human IgG. After the substrate reaction, the intensity of the colour developed is proportional to the amount of IgG-specific antibodies detected in the sample. Results for the samples are derived using the optical density ratio of the cut-off control and the patient sample and expressed in VE or Virotech Units which have been calibrated with the reference standard IgG anti-Pertussis toxin (Lot 3, 200 U/ml) of the Centre for Biologic Evaluation and Research (CBER), FDA. Three control sera will be used on each plate to assess inter-assay variation.

For peer review only

Appendix 1 - References

1. Satzke C, Turner P, Virolainen-Julkunen A, et al. Standard method for detecting upper respiratory carriage of Streptococcus pneumoniae: updated recommendations from the World Health Organization Pneumococcal Carriage Working Group. *Vaccine* 2013;32:165-79. doi:10.1016/j.vaccine.2013.08.062.
2. Balloch A, Licciardi PV, Leach A, et al. Results from an inter-laboratory comparison of pneumococcal serotype-specific IgG measurement and critical parameters that affect assay performance. *Vaccine* 2010;28:1333-40. doi:10.1016/j.vaccine.2009.11.011.
3. Licciardi PV, Toh ZQ, Clutterbuck EA, et al. No long-term evidence of hyporesponsiveness after use of pneumococcal conjugate vaccine in children previously immunized with pneumococcal polysaccharide vaccine. *The Journal of allergy and clinical immunology* 2016;137:1772-79 e11. doi:10.1016/j.jaci.2015.12.1303.
4. Satzke C, Dunne EM, Porter BD, et al. The PneuCarriage Project: A Multi-Centre Comparative Study to Identify the Best Serotyping Methods for Examining Pneumococcal Carriage in Vaccine Evaluation Studies. *PLoS medicine* 2015;12:e1001903; discussion e03. doi:10.1371/journal.pmed.1001903.
5. Porter BD, Ortika BD, Satzke C. Capsular Serotyping of Streptococcus pneumoniae by latex agglutination. *Journal of visualized experiments : JoVE* 2014:51747. doi:10.3791/51747.
6. Tenover FC, Huang MB, Rasheed JK, et al. Development of PCR assays to detect ampicillin resistance genes in cerebrospinal fluid samples containing Haemophilus influenzae. *J Clin Microbiol* 1994;32:2729-37.
7. Carter J, Hutton S, Sriprakash KS, et al. Culture of the causative organism of donovanosis (Calymmatobacterium granulomatis) in HEp-2 cells. *J Clin Microbiol* 1997;35:2915-7.
8. Price EP, Harris TM, Spargo J, et al. Simultaneous identification of Haemophilus influenzae and Haemophilus haemolyticus using real-time PCR. *Future microbiology* 2017;12:585-93. doi:10.2217/fmb-2016-0215.
9. Smith-Vaughan H, Byun R, Nadkarni M, et al. Measuring nasal bacterial load and its association with otitis media. *BMC Ear Nose Throat Disord* 2006;6:10. doi:10.1186/1472-6815-6-10.

For peer review only

APPENDIX 2

Plain Language Statements and Informed Consent Form

These materials were translated into Vietnamese, and back-translated into English, by FHI360. This trial uses two Plain Language Statements, one for participants enrolled at 2 months of age and randomised into Arms A-F, and one for participants enrolled at 18 months of age into Arm G. The same Informed Consent Form is used for participants in all Arms.

For peer review only

menzies school of health research

PO Box 41096, Casuarina NT 0811, Australia
John Mathews Building (Bldg 58),
Royal Darwin Hospital Campus, Rocklands Dve, Casuarina NT 0810
Ph: 08 8922 8196 | Fax: 08 8927 5187 | Web: www.menzies.edu.au
ABN: 70 413 542 847

discovery for a healthy tomorrow

INFORMATION SHEET: Vietnam Pneumococcal Vaccine Study

This is for you to keep.

Principal Investigators:
Assoc. Prof. Tran Ngoc Huu
Prof. Edward Kim Mulholland

Research Partners:
Pasteur Institute, Ho Chi Minh City
Menzies School of Health Research
Murdoch Childrens Research Institute

For peer review only

Introduction

Health research helps us to understand diseases and find ways to prevent them. Vaccines (like the routine baby injections) are an important way to prevent diseases. Pneumonia is a common problem in Vietnam and throughout the developing world. In the developing world it is the leading cause of death amongst under 5 year olds. A number of germs cause pneumonia but the most common germ is a bacteria called pneumococcus. Pneumococcus can also cause ear infections as well as other, more severe diseases like meningitis (infection around the brain). This germ normally lives in the nose of humans and is spread from person to person by touching or sneezing. There are more than 90 types of this germ but only some types cause serious infections in young children.

Why are we doing the study?

There are vaccines available to protect against infection with pneumococcus. These are called pneumococcal vaccines. Many countries around the world give all their babies a pneumococcal vaccine that protects against 7 types of the pneumococcal disease (7v-PCV). There are two new vaccines which have been developed. Both new vaccines give more protection against pneumococcal disease than the 7v-PCV. Both vaccines have completed all their tests and are licensed and being used by many countries in Europe and the United States. The clinical trials have shown that these vaccines are safe; therefore there is little danger to any child participating in this study. The vaccines are likely to provide some protection from ear infections and pneumonia. Unfortunately the costs of these vaccines are very high, so not all countries in the world can afford them. We are doing this study to find the best ways to protect babies from this germ and also to make it cheaper for countries, like Vietnam, to afford to buy the vaccine.

Benefits of the study

By joining the study your baby can be protected from the commonest pneumococcal germs. Both these vaccines are very expensive and are not presently available to other babies in Vietnam. They have been especially made for use in babies and young children and will protect the babies from the common diseases caused by the pneumococcus. We hope to find a schedule that works and which countries like Vietnam can afford. In addition children will receive 4 doses of *Infanrix-Hexa*: 3 doses during early infancy and a booster dose at either 18 or 19 months of age.

For peer review only - http://bmjopen.bmj.com/site/about/guidelines.xhtml

What does the study involve?

The study will include 1400 babies and we will be looking at 7 different vaccine schedules in this study. 1200 babies will be enrolled at 2 months old and will be randomly allocated to 1 of 6 groups. An additional 200 babies will be enrolled at 18 months old to act as controls.

Consent: A study doctor or nurse will discuss the study with each child's parent or legal guardian. They will explain what is involved and ask some questions about the baby's health. If you agree to join the study she will ask you to sign a consent form which says that you agree for your baby to join. If you consent to taking part in the study, she will perform a health check of your baby to make sure your baby is healthy to take part.

Vaccinations & health checks: If you agree to your baby to take part in the study you will need to come to the clinic between 9 and 11 times over a period of 22 months. The study nurse will remind you when you need to come. Like rolling a dice your baby will be allocated to 1 of 6 groups. Your baby will get between one and four doses of one of the two types of Pneumococcal vaccine, either the Prevnar-13 (13v-PCV which covers 13 types of the pneumococcal germ) or the 10v-Synflorix vaccine (which covers 10 types of the pneumococcal germ and may be better at protecting against pneumonia). Depending on which group your baby is randomly placed in will depend on when, how many doses and what type of Pneumococcal vaccine your baby will receive. Your baby will also get an infant vaccine (Infanrix-hexa 6-1) that covers all the diseases (diphtheria, tetanus, pertussis, hepatitis B, polio virus and *Haemophilus influenzae* type B) that are covered by the standard vaccines used in VIetnam. Vaccines will be given by staff from Pasteur Institute Ho Chi Minh City. Your baby will also have regular health checks during the study.

Questionnaire: At the start of the study you will be asked some general questions about your family and your baby's health. These are simply to help us understand how the vaccines work best. The results will be kept confidential (see below).

Blood tests: Up to four blood tests will be taken during the study, by staff from Children's Hospital Number 2. The blood tests are to check the response to the vaccines. If you would prefer, we can put local anesthetic cream on your baby's skin before taking the blood test so that it doesn't hurt as much. The amount of blood taken will vary depending on the age of the child: 2.0mls at 2 months of age; 3.5mls from 3 to 10 and 19 months of age; and 3.5mls or 7.5mls at 18 and 24 months of age.

Nose swabs: Six nose swabs will be taken during the study, at 2, 6, 9, 12, 18 and 24 months of age. The nose swabs are to see if the vaccine will help stop the spread of the pneumococcus from child to child. This will involve putting a cotton wool swab (like a cotton bud) into the baby's nose for a couple of seconds. This may make the baby sneeze and possibly cry briefly – it tickles quite a lot, but doesn't really hurt.

Summary of changes: Additional procedures and vaccines

Groups A-E	*18 months*	*Measles and Rubella given*
	19 months	*Infanrix Hexa given*
	24 months	*Nose swab taken*
Group F	*18 months*	*Infanrix Hexa given*
	19 months	*Measles and Rubella given*<br>*Blood taken*
	24 months	*Nose swab taken*<br>*Blood taken*<br>*Synflorix given*

Hospital record review: If your baby becomes unwell during the study, the staff may need to look at your child's medical records.

Menzies School of Health Research
PO Box 41096, Casuarina NT 0811, Australia | John Mathews Building (Bldg 58), Royal Darwin Hospital Campus, Rocklands Dve, Casuarina NT 0810
Ph: 08 8922 8196 | Fax: 08 8927 5187 | Web: www.menzies.edu.au | ABN 70 413 542 847

For peer review only - http://bmjopen.bmj.com/site/about/guidelines.xhtml

Are there any risks?

The vaccines we are using are licensed many countries. As with all vaccines there is likely to be some pain felt, and there is a small risk of soreness and redness where the vaccine was given. Babies in the study will get up to 4 extra injections than they would routinely get. We will check the babies to make sure they don't have any unexpected reactions. We also have a study doctor who will be keeping a record of any serious illnesses that are unlikely to occur during the study.

Confidentiality

All information collected in this study will remain confidential and will be used for research purposes only. All information will be kept secure. Your baby will be given an identification number at the start of the study. Any information collected will use this number and will not include your baby's name. The samples we collect will be sent to overseas laboratories to have further tests. These laboratories will not be given your child's name. We will ask your permission if it is alright for your baby's blood and nose swab samples to be stored indefinitely for other similar tests in the future. This would help us to perform any new pneumococcal test that may be developed in the future. The results of the study will be published in scientific journals and presented at conferences. There will never be details published that would identify your baby.

Voluntary Participation and Withdrawal from the Study

Your baby does not have to take part in the study. Your baby will get the best treatment available and the full attention of the health staff even if they do not participate. You are free to withdraw your baby from the study at any point. This will not affect any of your baby's further health care treatment and there will be no harmful consequences for your baby. If your baby has not had all their pneumococcal vaccines they may not be fully protected against the pneumococcal germs which most commonly affect infants. However, they will still gain some protection from the doses of vaccine received.

Compensation

We will pay 200,000VND towards the transport cost for coming to the clinic for each study visit. If your baby becomes ill or injured as a result of taking part in this clinical study, medical treatment will be provided.

Ethical Approval

This study has been approved by the People's Committee of Ho Chi Minh City. This study has also been approved by the Vietnam Ministry of Health Ethics Committee and the Menzies School of Health Research Ethics Committee, Australia. The ethics committees make sure that the study is being done in the best and safest way. If you have any concerns or complaints regarding the conduct of the research project you are invited to contact:

Vietnam Ministry of Health<br>Ethics Committee<br>Phone: 04 62732156	OR	Human Research Ethics Committee of the NT<br>Department of Health and Menzies School of Health Research<br>PO Box 41096, Casuarina, NT 0811, Australia<br>Phone: 61 8 8922 7922<br>Email: ethics@menzies.edu.au

How is the study funded?

The funding to perform the study is from the National Health and Medical Research Council, Australia and the Bill & Melinda Gates Foundation.

For peer review only - http://bmjopen.bmj.com/site/about/guidelines.xhtml

Your Right to Ask Questions

Please feel free to contact us if you have any questions or concerns.

If you have any questions regarding the study activities, please phone:

If you have any questions regarding adverse events, please phone:

Commune Health Centre Number:

For peer review only

For peer review only - http://bmjopen.bmj.com/site/about/guidelines.xhtml

menzies school of health research

PO Box 41096, Casuarina NT 0811, Australia
John Mathews Building (Bldg 58),
Royal Darwin Hospital Campus, Rocklands Dve, Casuarina NT 0810
Ph: 08 8922 8196 | Fax: 08 8927 5187 | Web: www.menzies.edu.au
ABN: 70 413 542 847

discovery for a healthy tomorrow

INFORMATION SHEET: Vietnam Pneumococcal Vaccine Study (Control group)

This is for you to keep.

Principal Investigators:
Assoc. Prof. Tran Ngoc Huu
Prof. Edward Kim Mulholland

Research Partners:
Pasteur Institute, Ho Chi Minh City
Menzies School of Health Research
Murdoch Children's Research Institute

For peer review only

Introduction

Health research helps us to understand diseases and find ways to prevent them. Vaccines (like the routine baby injections) are an important way to prevent diseases. Pneumonia is a common problem in Vietnam and throughout the developing world. In the developing world it is the leading cause of death amongst under 5 year olds. A number of germs cause pneumonia but the most common germ is a bacteria called pneumococcus. Pneumococcus can also cause ear infections as well as other, more severe diseases like meningitis (infection around the brain). This germ normally lives in the nose of humans and is spread from person to person by touching or sneezing. There are more than 90 types of this germ but only some types cause serious infections in young children.

Why are we doing the study?

There are vaccines available to protect against infection with pneumococcus. These are called pneumococcal vaccines. Many countries around the world give all their babies a pneumococcal vaccine that protects against 7 types of the pneumococcal disease (7v-PCV). There are two new vaccines which have been developed. Both new vaccines give more protection against pneumococcal disease than the 7v-PCV.Both vaccines have completed all their tests and are licensed and being used by many countries in Europe and the United States. The clinical trials have shown that these vaccines are safe; therefore there is little danger to any child participating in this study. The vaccines are likely to provide some protection from ear infections and pneumonia. Unfortunately the costs of these vaccines are very high, so not all countries in the world can afford them. We are doing this study to find the best ways to protect babies from this germ and also to make it cheaper for countries, like Vietnam, to afford to buy the vaccine.

Benefits of the study

By joining the study your baby can be protected from the commonest pneumococcal germs. Both these vaccines are very expensive and are not presently available to other babies in Vietnam. They have been especially made for use in babies and young children and will protect the babies from the common diseases caused by the pneumococcus. We hope to find a schedule that works and which countries like Vietnam can afford. In addition your baby will receive a dose of Infanrix-hexa at 18 months of age.

For peer review only - http://bmjopen.bmj.com/site/about/guidelines.xhtml

What does the study involve?

The study will include 200 babies to act as comparisons to participants in an existing study of six different vaccine schedules.

Consent: A study doctor or nurse will discuss the study with each child's parent or legal guardian. They will explain what is involved and ask some questions about the baby's health. If you agree to join the study she will ask you to sign a consent form which says that you agree for your baby to join. If you consent to taking part in the study, she will perform a health check of your baby to make sure your baby is healthy to take part.

Vaccinations & health checks: If you agree to your baby to take part in the study you will need to come to the clinic 3 times over a period of 6 months. The study nurse will remind you when you need to come. Your baby will get a single dose of (Infanrix-hexa 6-1) that covers six diseases (diphtheria, tetanus, pertussis, hepatitis B, polio virus and *Haemophilus influenzae* type B) at 18 months of age, a single dose of Measles and Rubella (MR) at 19 months of age and a single dose of Pneumococcal vaccine (10v-Synflorix vaccine, which covers 10 types of the pneumococcal germ) at 24 months of age. Vaccines will be given by staff from Pasteur Institute Ho Chi Minh City. Your baby will also have a doctor's health check at each study visit.

Questionnaire: At the start of the study you will be asked some general questions about your family and your baby's health. These are simply to help us understand how the vaccines work best. The results will be kept confidential (see below).

Blood tests: Three blood tests will be taken over the six months, by staff from Children's Hospital Number 2. The blood tests are to check the response to the vaccines. If you would prefer, we can put local anesthetic cream on your baby's skin before taking the blood test so that it doesn't hurt as much. The amount of blood taken will be 3.5 or 7.5mls at 18 and 24 months of age; and 3.5mls at 19 months of age.

Nose swabs: Two nose swabs will be taken during the study, at 18 and 24 months of age. The nose swabs are to see if the vaccine will help stop the spread of the pneumococcus from child to child. This will involve putting a cotton wool swab (like a cotton bud) into the baby's nose for a couple of seconds. This may make the baby sneeze and possibly cry briefly – it tickles quite a lot, but doesn't really hurt.

Hospital record review: If your baby becomes unwell during the study, the staff may need to look at your child's medical records.

Are there any risks?

The vaccines we are using are licensed many countries. As with all vaccines there is likely to be some pain felt, and there is a small risk of soreness and redness where the vaccine was given. We will check the babies to make sure they don't have any unexpected reactions. We also have a study doctor who will be keeping a record of any serious illnesses that are unlikely to occur during the study.

Confidentiality

All information collected in this study will remain confidential and will be used for research purposes only. All information will be kept secure. Your baby will be given an identification number at the start of the study. Any information collected will use this number and will not include your baby's name. The samples we collect will be sent to overseas laboratories to have further tests. These laboratories will not be given your child's name. We will ask your permission if it is alright for your baby's blood and nose swab samples to be stored indefinitely for other similar tests in the future. This would help us to perform any new pneumococcal test that may be developed in the

For peer review only - http://bmjopen.bmj.com/site/about/guidelines.xhtml

future. The results of the study will be published in scientific journals and presented at conferences. There will never be details published that would identify your baby.

Voluntary Participation and Withdrawal from the Study

Your baby does not have to take part in the study. Your baby will get the best treatment available and the full attention of the health staff even if they do not participate. You are free to withdraw your baby from the study at any point. This will not affect any of your baby's further health care treatment and there will be no harmful consequences for your baby. If your baby has not had all their pneumococcal vaccines they may not be fully protected against the pneumococcal germs which most commonly affect infants. However, they will still gain some protection from the doses of vaccine received.

Compensation

We will pay 200,000VND towards the transport cost for coming to the clinic for each study visit. If your baby becomes ill or injured as a result of taking part in this clinical study, medical treatment will be provided.

Ethical Approval

This study has been approved by the People's Committee of Ho Chi Minh City. This study has also been approved by the Vietnam Ministry of Health Ethics Committee and the Menzies School of Health Research Ethics Committee, Australia. The ethics committees make sure that the study is being done in the best and safest way. If you have any concerns or complaints regarding the conduct of the research project you are invited to contact:

Vietnam Ministry of Health<br>Ethics Committee<br>Phone: 04 62732156	OR	Human Research Ethics Committee of the NT<br>Department of Health and Menzies School of Health Research<br>PO Box 41096, Casuarina, NT 0811, Australia<br>Phone: 61 8 8922 7922<br>Email: ethics@menzies.edu.au

How is the study funded?

The funding to perform the study is from the National Health and Medical Research Council, Australia and the Bill & Melinda Gates Foundation.

Your Right to Ask Questions

Please feel free to contact us if you have any questions or concerns.

If you have any questions regarding the study activities, please phone:

If you have any questions regarding adverse events, please phone:

Commune Health Centre Number:

For peer review only - http://bmjopen.bmj.com/site/about/guidelines.xhtml

menzies
school of health research

PO Box 41096, Casuarina NT 0811, Australia
John Mathews Building (Bldg 58),
Royal Darwin Hospital Campus, Rocklands Dve, Casuarina NT 0810
Ph: 08 8922 8196 | Fax: 08 8927 5187 | Web: www.menzies.edu.au
ABN: 70 413 542 847

discovery for a healthy tomorrow

CONSENT FORM

This means you can say NO

Screening Number: |___|___|___|___|

Participant ID: |___|___|___|___|

Date: __ __ /__ __ /__ __
dd / mm / yy

Principal Investigators:

Assoc. Prof. Tran Ngoc Huu
Prof. Edward Kim Mulholland

Research Partners:

Pasteur Institute, Ho Chi Minh City
Menzies School of Health Research

This form is to record if you agree for your infant to take part in the **"Evaluation of Different Infant Vaccination Schedules Incorporating Pneumococcal Vaccination"**. You should only sign this form if you are happy that the information about the study has been clearly explained to you, you have received enough information about the study and you have had all your questions answered satisfactorily.

Please record the name of the person you have spoken to about the study:

____

By agreeing for your infant to take part in the study, you understand that:

- You are free to withdraw your child from the study at any time without having to give a reason;
- Your child will be vaccinated against all the diseases that are covered by the standard vaccines used in Vietnam, although these vaccines may be given at different times;
- If your child becomes sick, their hospital records will be reviewed by the study doctor or other designated study staff; and
- The samples taken in this study will be sent to overseas laboratories to test vaccine responses and carriage of bacteria

For peer review only

For peer review only - http://bmjopen.bmj.com/site/about/guidelines.xhtml

menzies school of health research

PO Box 41096, Casuarina NT 0811, Australia
John Mathews Building (Bldg 58),
Royal Darwin Hospital Campus, Rocklands Dve, Casuarina NT 0810
Ph: 08 8922 8196 | Fax: 08 8927 5187 | Web: www.menzies.edu.au
ABN: 70 413 542 847

discovery for a healthy tomorrow

Screening Number: |___|___|___|___|

Participant ID: |___|___|___|___|

Date: __ __ /__ __ /__ __

Consent:

❑ YES, I agree for my infant to take part in this study.

❑ NO, I do not agree for my infant to take part in this study.

Use of samples:

❑ YES, you may indefinitely store my unused identified samples for future work in the same general area of research that has obtained ethics committee approval.

❑ NO, you may **NOT USE** my samples for future research. Destroy my unused samples at the close of the study.

Signed (parent/legal guardian): ______________________ Date: __ __ /__ __ /__ __
dd / mm / yy

Name of parent/legal guardian: ______________________ Time: __ __ :__ __
hh : mm

Relationship to infant: ______________________

Name of infant or baby of: ______________________

Infant Sex: male / female Infant DOB: __ __ /__ __ /__ __
dd / mm / yy

Signed (study nurse): ______________________ Date: __ __ /__ __ /__ __
dd / mm / yy

If illiterate: *A literate witness must sign (if possible, this person should be selected by the participant and should have no connection to the research team).*

I have witnessed the accurate reading of the consent form to the parent of the potential participant, and the individual has had the opportunity to ask questions. I confirm that the individual has given consent freely.

Signed (witness): ______________________ Date: __ __ /__ __ /__ __
dd / mm / yy

Name of witness: ______________________

For peer review only

For peer review only - http://bmjopen.bmj.com/site/about/guidelines.xhtml

STANDARD PROTOCOL ITEMS: RECOMMENDATIONS FOR INTERVENTIONAL TRIALS

SPIRIT 2013 Checklist: Recommended items to address in a clinical trial protocol and related documents*

Section/item	Item No	Description	Addressed on page number
**Administrative information**			
Title	1	Descriptive title identifying the study design, population, interventions, and, if applicable, trial acronym	______3_______
Trial registration	2a	Trial identifier and registry name. If not yet registered, name of intended registry	______3_______
	2b	All items from the World Health Organization Trial Registration Data Set	______3_______
Protocol version	3	Date and version identifier	______5_______
Funding	4	Sources and types of financial, material, and other support	______30______
Roles and responsibilities	5a	Names, affiliations, and roles of protocol contributors	______29______
	5b	Name and contact information for the trial sponsor	______5_______
	5c	Role of study sponsor and funders, if any, in study design; collection, management, analysis, and interpretation of data; writing of the report; and the decision to submit the report for publication, including whether they will have ultimate authority over any of these activities	______5_______
	5d	Composition, roles, and responsibilities of the coordinating centre, steering committee, endpoint adjudication committee, data management team, and other individuals or groups overseeing the trial, if applicable (see Item 21a for data monitoring committee)	______n/a______

For peer review only

For peer review only - http://bmjopen.bmj.com/site/about/guidelines.xhtml

Introduction

Background and rationale	6a	Description of research question and justification for undertaking the trial, including summary of relevant studies (published and unpublished) examining benefits and harms for each intervention	______6_______
	6b	Explanation for choice of comparators	______8_______
Objectives	7	Specific objectives or hypotheses	______9______
Trial design	8	Description of trial design including type of trial (eg, parallel group, crossover, factorial, single group), allocation ratio, and framework (eg, superiority, equivalence, noninferiority, exploratory)	______11______

Methods: Participants, interventions, and outcomes

Study setting	9	Description of study settings (eg, community clinic, academic hospital) and list of countries where data will be collected. Reference to where list of study sites can be obtained	______12______
Eligibility criteria	10	Inclusion and exclusion criteria for participants. If applicable, eligibility criteria for study centres and individuals who will perform the interventions (eg, surgeons, psychotherapists)	______12______
Interventions	11a	Interventions for each group with sufficient detail to allow replication, including how and when they will be administered	______13______
	11b	Criteria for discontinuing or modifying allocated interventions for a given trial participant (eg, drug dose change in response to harms, participant request, or improving/worsening disease)	______14______
	11c	Strategies to improve adherence to intervention protocols, and any procedures for monitoring adherence (eg, drug tablet return, laboratory tests)	______14______
	11d	Relevant concomitant care and interventions that are permitted or prohibited during the trial	______14______
Outcomes	12	Primary, secondary, and other outcomes, including the specific measurement variable (eg, systolic blood pressure), analysis metric (eg, change from baseline, final value, time to event), method of aggregation (eg, median, proportion), and time point for each outcome. Explanation of the clinical relevance of chosen efficacy and harm outcomes is strongly recommended	______15______
Participant timeline	13	Time schedule of enrolment, interventions (including any run-ins and washouts), assessments, and visits for participants. A schematic diagram is highly recommended (see Figure)	______17______

For peer review only - http://bmjopen.bmj.com/site/about/guidelines.xhtml

Sample size	14	Estimated number of participants needed to achieve study objectives and how it was determined, including clinical and statistical assumptions supporting any sample size calculations	______18______
Recruitment	15	Strategies for achieving adequate participant enrolment to reach target sample size	______19______

Methods: Assignment of interventions (for controlled trials)

Allocation:			
Sequence generation	16a	Method of generating the allocation sequence (eg, computer-generated random numbers), and list of any factors for stratification. To reduce predictability of a random sequence, details of any planned restriction (eg, blocking) should be provided in a separate document that is unavailable to those who enrol participants or assign interventions	______19______
Allocation concealment mechanism	16b	Mechanism of implementing the allocation sequence (eg, central telephone; sequentially numbered, opaque, sealed envelopes), describing any steps to conceal the sequence until interventions are assigned	______19______
Implementation	16c	Who will generate the allocation sequence, who will enrol participants, and who will assign participants to interventions	______19______
Blinding (masking)	17a	Who will be blinded after assignment to interventions (eg, trial participants, care providers, outcome assessors, data analysts), and how	______19______
	17b	If blinded, circumstances under which unblinding is permissible, and procedure for revealing a participant's allocated intervention during the trial	______n/a______

Methods: Data collection, management, and analysis

Data collection methods	18a	Plans for assessment and collection of outcome, baseline, and other trial data, including any related processes to promote data quality (eg, duplicate measurements, training of assessors) and a description of study instruments (eg, questionnaires, laboratory tests) along with their reliability and validity, if known. Reference to where data collection forms can be found, if not in the protocol	______20______
	18b	Plans to promote participant retention and complete follow-up, including list of any outcome data to be collected for participants who discontinue or deviate from intervention protocols	______20______

For peer review only - http://bmjopen.bmj.com/site/about/guidelines.xhtml

Data management	19	Plans for data entry, coding, security, and storage, including any related processes to promote data quality (eg, double data entry; range checks for data values). Reference to where details of data management procedures can be found, if not in the protocol	______20______
Statistical methods	20a	Statistical methods for analysing primary and secondary outcomes. Reference to where other details of the statistical analysis plan can be found, if not in the protocol	______20______
	20b	Methods for any additional analyses (eg, subgroup and adjusted analyses)	______21______
	20c	Definition of analysis population relating to protocol non-adherence (eg, as randomised analysis), and any statistical methods to handle missing data (eg, multiple imputation)	______22______
**Methods: Monitoring**			
Data monitoring	21a	Composition of data monitoring committee (DMC); summary of its role and reporting structure; statement of whether it is independent from the sponsor and competing interests; and reference to where further details about its charter can be found, if not in the protocol. Alternatively, an explanation of why a DMC is not needed	______22______
	21b	Description of any interim analyses and stopping guidelines, including who will have access to these interim results and make the final decision to terminate the trial	______22______
Harms	22	Plans for collecting, assessing, reporting, and managing solicited and spontaneously reported adverse events and other unintended effects of trial interventions or trial conduct	______23______
Auditing	23	Frequency and procedures for auditing trial conduct, if any, and whether the process will be independent from investigators and the sponsor	______23______
**Ethics and dissemination**			
Research ethics approval	24	Plans for seeking research ethics committee/institutional review board (REC/IRB) approval	______23______
Protocol amendments	25	Plans for communicating important protocol modifications (eg, changes to eligibility criteria, outcomes, analyses) to relevant parties (eg, investigators, REC/IRBs, trial participants, trial registries, journals, regulators)	______23______

For peer review only - http://bmjopen.bmj.com/site/about/guidelines.xhtml

Consent or assent	26a	Who will obtain informed consent or assent from potential trial participants or authorised surrogates, and how (see Item 32)	______24______
	26b	Additional consent provisions for collection and use of participant data and biological specimens in ancillary studies, if applicable	______24______
Confidentiality	27	How personal information about potential and enrolled participants will be collected, shared, and maintained in order to protect confidentiality before, during, and after the trial	______24______
Declaration of interests	28	Financial and other competing interests for principal investigators for the overall trial and each study site	______30______
Access to data	29	Statement of who will have access to the final trial dataset, and disclosure of contractual agreements that limit such access for investigators	______24______
Ancillary and post-trial care	30	Provisions, if any, for ancillary and post-trial care, and for compensation to those who suffer harm from trial participation	______24______
Dissemination policy	31a	Plans for investigators and sponsor to communicate trial results to participants, healthcare professionals, the public, and other relevant groups (eg, via publication, reporting in results databases, or other data sharing arrangements), including any publication restrictions	______25______
	31b	Authorship eligibility guidelines and any intended use of professional writers	______25______
	31c	Plans, if any, for granting public access to the full protocol, participant-level dataset, and statistical code	______25______
**Appendices**			
Informed consent materials	32	Model consent form and other related documentation given to participants and authorised surrogates	_Appendices.pdf_
Biological specimens	33	Plans for collection, laboratory evaluation, and storage of biological specimens for genetic or molecular analysis in the current trial and for future use in ancillary studies, if applicable	_Appendices.pdf_

*It is strongly recommended that this checklist be read in conjunction with the SPIRIT 2013 Explanation & Elaboration for important clarification on the items. Amendments to the protocol should be tracked and dated. The SPIRIT checklist is copyrighted by the SPIRIT Group under the Creative Commons "Attribution-NonCommercial-NoDerivs 3.0 Unported" license.

For peer review only - http://bmjopen.bmj.com/site/about/guidelines.xhtml
